# Methods to identify and prioritize patient-centered outcomes for use in comparative effectiveness research

**DOI:** 10.1186/s40814-018-0284-6

**Published:** 2018-06-12

**Authors:** Evan Mayo-Wilson, Asieh Golozar, Terrie Cowley, Nicole Fusco, Gillian Gresham, Jennifer Haythornthwaite, Elizabeth Tolbert, Jennifer L. Payne, Lori Rosman, Susan Hutfless, Joseph K. Canner, Kay Dickersin

**Affiliations:** 1 Department of Epidemiology, Johns Hopkins Bloomberg School of Public Health, Baltimore, MD USA; 2 The TMJ Association, Ltd., Milwaukee, WI USA; 3 Center for Mind and Body Research, Johns Hopkins University School of Medicine, Baltimore, MD USA; 4 Johns Hopkins University Peabody Institute, Baltimore, MD USA; 5 Department of Psychiatry, Johns Hopkins University School of Medicine, Baltimore, MD USA; 6 Welch Medical Library, Johns Hopkins University School of Medicine, Baltimore, MD USA; 7 Department of Gastroenterology and Hepatology, Johns Hopkins School of Medicine, Baltimore, MD USA; 8 Department of Surgery, Johns Hopkins University School of Medicine, 600 North Wolfe Street, Baltimore, MD USA

## Abstract

**Background:**

We used various methods for identifying and prioritizing patient-centered outcomes (PCOs) for comparative effectiveness research (CER).

**Methods:**

We considered potential PCOs (“benefits” and “harms”) related to (1) gabapentin for neuropathic pain and (2) quetiapine for bipolar depression. Part 1 (April 2014 to March 2015): we searched for PCO research and core outcome sets (COSs). We conducted electronic searches of bibliographic databases and key websites and examined FDA prescribing information and reports of clinical trials and systematic reviews. We asked patient and clinician co-investigators to identify PCOs. Part 2 (not part of our original study protocol): in 2015, we surveyed members of The TMJ Association, Ltd., a patient group associated with temporomandibular disorders (4130 invitations sent). Participants prioritized (1) the importance of six potential benefits and (2) 21 potential harms selected by the investigators in part 1, using stated preference methods. We calculated descriptive statistics.

**Results:**

In part 1, we identified a COS for pain, the Initiative on Methods, Measurement, and Pain Assessment in Clinical Trials (IMMPACT) recommendations. The COS identified several important benefits, but it lacked specific recommendations about which potential harms to include in CER. We did not identify a COS for bipolar depression. Research reports, prescribing information, and patient co-investigators helped identify but not prioritize outcomes. We abandoned our electronic search for PCO research because we found it would be resource-intensive and yield few relevant reports. In part 2, surveying patients was useful for prioritizing PCOs. Members of The TMJ Association, Ltd., completed the survey (*N* = 746) and successfully prioritized both benefits and harms. Participants did not identify many benefits other than those we identified in part 1; several participants identified additional harms.

**Conclusions:**

These exploratory results could inform future research about identifying and prioritizing PCOs. We found that stakeholder co-investigators and research reports contributed to identifying PCOs; surveying a patient group contributed to prioritizing PCOs. Prioritizing potential harms was particularly challenging because there are many more potential harms than potential benefits. Methods for identifying and prioritizing potential benefits for CER might not be appropriate for harms. Further research is needed to determine the generalizability of these results.

**Electronic supplementary material:**

The online version of this article (10.1186/s40814-018-0284-6) contains supplementary material, which is available to authorized users.

## Background

The role of patients in health research has been expanding since the 1980s, when activists succeeded in prioritizing patient interests for AIDS research [1]. Patients are now involved in generating and synthesizing evidence [2, 3], reviewing research applications [4], regulatory approval [5], and the development of clinical practice guidelines [6]. Although there appears to be broad consensus that both patients and patient-centered outcomes (PCOs) should be included in health research [7], questions remain about the specific methods that should be used to engage patients in the effort. For example, many comparative effectiveness studies include patient investigators. Funders and journals increasingly engage patients in reviewing research proposals and reports. Yet engaging a few patients might be insufficient to ensure that comparative effectiveness research includes the most important PCOs. There is little evidence about how many patients to engage, and there is little evidence comparing the relative advantages of engaging patient investigators and reviewers compared with other methods of patient engagement.

Even when PCOs are appropriately *identified*, it is not clear how the “most important” PCOs should be *prioritized*, either for primary research (e.g., clinical trials) or for systematic reviews [8, 9]. Literature searches for PCO research, relevant clinical trials and systematic reviews, FDA prescribing information, drug compendia, and patient websites are likely to identify many outcomes that have been examined previously. However, methods are needed to prioritize outcomes, that is, to determine which outcomes identified in these sources are most important to patients.

Developing core outcome sets (COSs), which are outcomes to be included in all studies of a condition or intervention [10], is one approach to identifying and prioritizing outcomes for use in comparative effectiveness research (CER). When patients are involved in their development, COSs could improve the patient-centeredness of research. The widespread use of COSs is likely to improve the usefulness of CER by increasing opportunities to combine results across studies of the same health problem using meta-analysis. At this time, however, COSs do not exist for every condition and intervention [11]. Until COSs are available for more problems and populations, investigators need pragmatic strategies for engaging patients in CER [6, 12–14].

## Objective

To explore various methods for identifying and prioritizing PCOs for examination in two case studies.

## Methods

This article is part of a Patient-Centered Outcomes Research Institute (PCORI) funded investigation about whether different sources of information for the same randomized controlled trials (RCTs) would affect the results of systematic reviews of patient-centered outcomes research. Our research (Integrating Multiple Data Sources for Meta-Analysis (MUDS)) included two case studies, [1] gabapentin for adults with neuropathic pain and [2] quetiapine for adults with bipolar depression. The research team included four stakeholder co-investigators (two patients and two practicing clinicians). This article and supplements describe all the eligibility criteria relevant to this part of the study; we have described detailed eligibility criteria for other parts of the study elsewhere [15].

This article focuses on the first aim of MUDS, which was to determine whether eligible clinical trial reports addressed PCOs. For this aim, we first needed to develop a list of PCOs that clinical trials might have assessed for each case.

In part 1 of this article, we describe how we explored a variety of sources and worked with patient and clinician co-investigators to identify PCOs for neuropathic pain and bipolar depression. In part 2 of this article, we describe an exploratory survey of patients in which we used stated preference methods to prioritize PCOs for pain. Part 2 was not part of our original research protocol and involved only one case because we had two years of funding and because conducting two exploratory surveys was not necessary to the overarching goals of our funded project.

### Part 1: identifying patient-centered outcomes

#### Systematic search for previous patient engagement studies

Led by an experienced informationist (LR), we conducted systematic searches of bibliographic databases for previous PCO research in which participants identified or prioritized PCOs for neuropathic pain or bipolar depression (“PCO research”). Because we sought to include all relevant studies, including both qualitative and quantitative research, we did not limit the searches by study design. To identify studies about PCOs related to neuropathic pain, we searched Ovid MEDLINE on September 5, 2014. For PCO research related to bipolar depression, we searched Ovid MEDLINE, Embase, the Cochrane Central Register of Controlled Trials (CENTRAL), PsycINFO (EBSCO), CINAHL (EBSCO), and PubMed (to retrieve records not yet in MEDLINE [Ovid]) on September 30, 2014 (Additional file 1).

#### Core outcome sets

For each condition, we asked co-investigators and colleagues to identify COSs related to neuropathic pain and bipolar disorder. We searched the Core Outcome Measures in Effectiveness Trials (COMET) database [11] in April 2014, and the James Lind Alliance (http://www.jla.nihr.ac.uk) in May 2014, to identify COSs. The James Lind Alliance is a nonprofit organization dedicated to identifying research priorities related to intervention effects.

#### Previous trials and reviews eligible for the MUDS study

Because previous CER includes many outcomes that patients might consider important, we recorded the outcomes included in reports of clinical trials that were eligible for our overall study [15]. Search strategies for eligible trials and results of the searches have been described elsewhere [15–17]. We included both public reports (e.g., journal articles, trial registrations, conference abstracts, FDA medical and statistical reviews) and non-public reports (e.g., protocols, clinical study reports) of clinical trials. We identified 21 trials and 74 reports for gabapentin-neuropathic pain, and we identified 7 trials and 50 reports for quetiapine-bipolar depression [17]. We also examined recent systematic reviews on the topics [18, 19].

#### Food and Drug Administration prescribing information (package inserts)

Because prescribing information might include outcomes that are important to patients, even if patients are unaware of those outcomes, we examined all outcomes in the prescribing information for both case studies.

We examined the Drugs@FDA website in April 2014 using the generic drug names “gabapentin” and “quetiapine” to identify prescribing information (i.e., the “package inserts”). (Since November 2016, Drugs@FDA can be accessed through the website https://www.accessdata.fda.gov/scripts/cder/daf/.) For a given drug, prescribing information described the specific health conditions that the drug might be used to treat, and it included information that might apply to people who use the drug for any condition.

#### DRUGDEX compendium

Like prescribing information, drug compendia might include outcomes that are important to patients. Thus, we examined the compendium “DRUGDEX” (which was available at http://micromedex.com) on January 22, 2015. DRUGDEX includes information about on- and off-label uses of medications; the Centers for Medicare and Medicaid Services (CMS) may use DRUGDEX to make reimbursement decisions for anti-cancer treatments [20, 21]. Like FDA prescribing information, DRUGDEX is organized by intervention and includes both information about specific conditions and information about the use of drugs for any condition.

#### PatientsLikeMe

We examined the website PatientsLikeMe (http://www.patientslikeme.com) on April 29, 2014. Information on PatientsLikeMe is entered by patients. PatientsLikeMe allows users to search for (“filter”) information about potential benefits for both intervention and indication. We searched for information about gabapentin used for treating “neuropathic pain” and quetiapine used for treating “bipolar disorder.” For each indication-intervention pair, patients assess the intervention’s perceived “effectiveness” using a 5-point scale (major, moderate, slight, none, can’t tell). PatientsLikeMe allows users to search for information about potential harms (called “side effects”) only by intervention. For each intervention, patients assess the broad label side effects using a 4-point scale (severe, moderate, mild, none). Although patients can list specific side effects (e.g., dizziness), they cannot rate specific side effects using the scales above. PatientsLikeMe creates summary tables that report the number of patients who say they experienced each specific side effect (e.g., dizziness). Finally, PatientsLikeMe includes patient narratives (unstructured qualitative data), which we did not review because of time constraints.

#### Engaging patient and clinician co-investigators

In addition to using the methods above, we invited patient and clinician investigators to identify PCOs that they considered important.

Aim 1 of MUDS was to determine whether the RCT reports that were eligible for our study addressed PCOs; our goal was to develop two lists of PCOs, one for each case study. Because part 1 was exploratory, and we used a variety of methods and sources to identify PCOs, we identified more PCOs than we could include in our study; thus, we attempted to prioritize the list by engaging stakeholder co-investigators. We asked patient and clinician co-investigators to describe the importance of the outcomes we had identified by assigning each outcome to one of three categories: “Definitely analyze, very important to patients”; “Possibly analyze, sometimes important to patients or important to some patients”; or “Definitely do not analyze, not important to patients” (Additional file 2). These co-investigators were invited to add any outcomes we had not identified using other methods. Our patient co-investigators did not feel confident, however, that their preferences would represent those of other patients who might have different preferences related to their demographic characteristics (e.g., sex, age), clinical characteristics (e.g., comorbid conditions), or personal values.

### Part 2: prioritizing patient-centered outcomes for pain

Our findings in part 1 led us to explore in part 2, the prioritization of outcomes that we had identified for people with pain.

#### Preparing for the patient survey

We conducted a survey asking patients affiliated with a patient group to rank the importance of outcomes related to the treatment of pain. Two of the authors (EMW and SH) selected “outcome domains” [16, 22] related to six potential benefits of treatment and 21 potential harms from all those identified in part 1 to be further prioritized using the part 2 survey (Additional file 3). We recruited survey participants (no identifying information was collected) from the TMJ Association, Ltd., a group founded and run by one of the patient co-investigators (TC). We used SurveyMonkey (LLC, Palo Alto, CA) to conduct the survey. We received an exemption for the survey from the Johns Hopkins Bloomberg School of Public Health Institutional Review Board (IRB no 00006324). We did not conduct a similar study in patients with bipolar depression because we were not working with a group of patients we could survey.

#### Part 2 survey questions: prioritizing six specific benefits and side effects

The survey first asked participants to rank the importance of the six potential benefits of interventions used for pain. In part 1 of our study, we identified a relatively small number of potential beneficial outcome domains (see Additional file 2); we selected the domains related to gabapentin-neuropathic pain that we found most frequently for further comparison. These were pain relief, improvement in your ability to do normal activities, improvement in sleep, changes in the overall quality of your life, changes in mood, and reduction in the need for other pain medication. We also asked survey participants to include in their ranking one item representing all potential harms (side effects). We asked how much each outcome would affect a participant’s decision to use or not to use an intervention. To rank the six specific benefits and side effects, survey participants assigned values from 1 (most affects your decision) to 7 (least affects your decision). We also asked participants to describe in a text box any additional potential benefits they might seek from treatment.

#### Part 2 survey questions: prioritizing 21 specific harms

In part 1, we identified hundreds of terms that were used to describe potential harms of treatment. To limit the number of questions in our survey and to avoid redundant questions, two authors (EMW and SH) grouped terms describing potential harms into 21 items. For example, we grouped terms related to skin into one item “skin problems (e.g., dry skin, acne, rash).” We then reviewed these categorizations with patient and clinician co-investigators and modified the groupings if appropriate.

To minimize the number of items we asked each participant to rank on the survey, we asked each participant, depending on his or her birth month, to rank only seven of the 21 harms. To do this, we made 12 sets of seven harms; each harm appeared with equal frequency across the survey “sets” (i.e., each set comprised seven harms and each harm appeared in four sets). For each set of seven harms, we asked participants to assign values from 1 (the potential harm that would most affect your decision to use an intervention) to 7 (would least affect your decision to use an intervention).

After participants ranked seven harms, the survey questions asked if there were any other potential harms they would want to know about before starting treatment. Participants could identify additional harms in a text box.

We then asked patients to select whether (1) “the likelihood that the medication will reduce your symptoms” (i.e., benefits) or (2) “the likelihood that you will experience side effects” (i.e., harms) was more important in making a decision about treatment.

#### Part 2 survey questions: participant characteristics

The survey concluded with questions about participant characteristics. We asked each participant for his or her year of birth, gender, types of pain experienced currently and in the past, “present pain intensity” (PPI), current and previous pain treatments, duration of pain, and harms they had experienced because of treatment.

#### Recruitment

On June 17, 2015, TC sent an email invitation to 4130 people on the TMJ Association mail list. To be included, people had to be subscribed to the TMJ e-newsletter and must have opened at least one e-newsletter in 2015. TC sent a reminder on July 1, 2015, and a second reminder on July 31, 2015. We considered invitees to have declined participation if we received a declining email or no response. The number of surveyed individuals (sample size) was opportunistic; it was determined by our ability to contact members of this patient group in collaboration with our patient investigators.

#### Statistical analysis

**Fig. 1:**
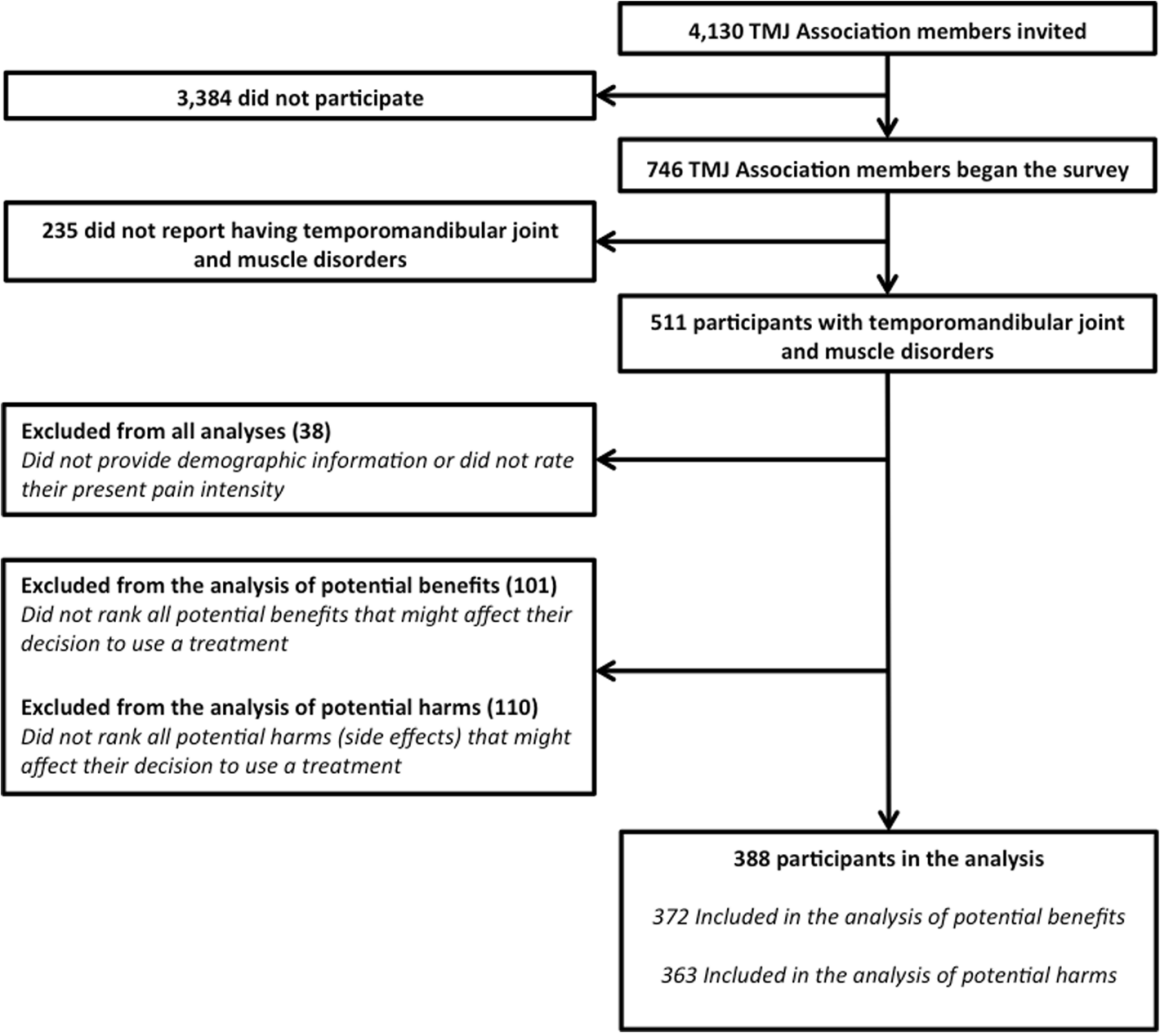
Flow chart for selection of study participants

We present descriptive statistics for part 2. We included survey participants in our analysis if they (1) indicated that their pain condition(s) included “TMJ (temporomandibular joint and muscle disorders),” (2) ranked the importance of the six benefits and one side effects item *or* ranked the importance of seven potential harms; and (3) reported their age, sex, and current PPI (Fig. 1).

We did not include two Initiative on Methods, Measurement, and Pain Assessment in Clinical Trials (IMMPACT) recommendations outcomes in part 2 survey of TMJ Association members: (1) “participant ratings of global improvement and satisfaction with treatment” was considered inappropriate for the survey because it was a composite outcome including multiple outcome domains and (2) “participant disposition” was not considered a potential PCO because it was defined described as “information regarding participant recruitment and progress through the trial, including all information specified in the CONSORT guidelines” [23].

To summarize the results, we first averaged individual patient rankings to derive an overall ranking of potential beneficial outcome domains and the single outcome item side effects from 1 to 7.

To summarize the individual part 2 survey participant rankings of harms, we used best-worst scaling (BWS) to compute a BWS score for each of the seven harms ranked by each survey participant [24]. First, we identified the harm that each participant ranked 1 (“most affects your decision” to use or not use a drug) and the harm that each participant ranked 7 (“least affects your decision” to use or not use a drug). Second, among participants who ranked the same set of seven harms, we subtracted the proportion of participants who ranked each potential harm “1” from the proportion of participants who ranked each harm “7” to obtain a BWS score for each potential harm in the set. Finally, we averaged the BWS scores across the 12 sets to obtain an average BWS score for each of the 21 items, and we ranked the 21 harms according to their BWS scores.

Because each part 2 survey participant ranked only seven of the 21 possible harms, we compared the characteristics of the 12 groups to check our assumption that the 12 groups did not differ and that computing an overall ranking would be acceptable.

We compared the results of the part 2 survey with the results of the methods used in part 1, including the opinions of our patient and stakeholder co-investigators.

All analyses were performed using Stata 14 (College Station, TX: StataCorp LP).

## Results

### Part 1: identifying patient-centered outcomes

#### Systematic search for published patient-centered outcomes research

Using systematic searches to identify published studies that aimed to identify or to prioritize PCOs for neuropathic pain and bipolar depression, we obtained a large number of titles and abstracts for each condition. For example, we retrieved 11,326 titles and abstracts in MEDLINE (Ovid) related to neuropathic pain and we retrieved 8801 titles and abstracts in the PubMed searches related to bipolar depression (Additional file 1).

We reviewed a random sample of titles and abstracts, and we found very few studies about patient preferences. We concluded that we would need to review tens of thousands of titles and abstracts to complete a comprehensive search; given our low success rate, we would probably identify only a few relevant studies. Based on these results, we decided that this was not a feasible approach to identify PCO research for our purposes. Thus, we did not complete a systematic search for published PCO research as part of our study.

#### Core outcome sets

One of our co-investigators (JH) identified a COS for pain, the IMMPACT recommendations [23, 25]. IMMPACT recommendations were developed through meetings with researchers, clinicians, industry, and patients, and through a survey of patients with pain [26]. According to the group’s website (http://www.immpact.org), many participants in IMMPACT meetings have been employed by pharmaceutical manufacturers. Other participants have worked in academia or government. Between zero and three patients have contributed to each of the 20 IMMPACT meetings, representing at most 3/39 (8%) of participants. IMMPACT recommends that six core outcome domains be assessed in studies of chronic pain: pain (includes “pain intensity” and “pain quality”), physical functioning, emotional functioning, participant ratings of global improvement and satisfaction with treatment, symptoms and adverse events, and participant disposition (Table 1).

**Table 1:** Potential benefits of treatment identified in different sources

	Gabapentin for neuropathic pain	Quetiapine for bipolar depression
Previous trials and systematic reviews	(1) Mood (2) Pain intensity (3) Quality of life (4) Satisfaction with treatment (5) Sleep disturbance (6) Use of other pain medications	(1) Anxiety (2) Depression (3) Functioning (4) Quality of life (5) Sleep
FDA prescribing information^a^	“NEURONTIN is indicated for: Postherpetic neuralgia in adults”	“SEROQUEL is indicated as monotherapy for the acute treatment of depressive episodes associated with bipolar disorder.”
Compendia (DRUGDEX)^a^	“Relief of pain associated with postherpetic neuralgia is indicative of a therapeutic response to gabapentin.”	“Regular- and extended-release quetiapine is indicated for the acute treatment of depressive episodes associated with bipolar disorder…”
PatientsLikeMe	“Neuropathic pain”	“Bipolar disorder”
Core outcomes sets (COS)	*Pain* 11-point (0–10) numerical rating scale of pain intensity Usage of rescue analgesics Categorical rating of pain intensity (none, mild, moderate, severe) in circumstances in which numerical ratings may be problematic *Physical functioning (either one of two measures)* Multidimensional Pain Inventory Interference Scale Brief Pain Inventory interference items *Emotional functioning (at least one of two measures)* Beck Depression Inventory Profile of Mood States *Participant ratings of global improvement and satisfaction with treatment* Patient Global Impression of Change *Symptoms and adverse events* Passive capture of spontaneously reported adverse events and symptoms and use of open-ended prompts *Participant disposition* Detailed information regarding participant recruitment and progress through the trial, including all information specified in the CONSORT guidelines [23]	No relevant core outcome set

Outcomes identified through the part 2 survey of patients with pain are included in Additional file 6

^a^We extracted only information related to the use of gabapentin for neuropathic pain and the use of quetiapine for bipolar depression. FDA prescribing information and DRUGDEX also described other uses of these drugs, which were not relevant to our study

We did not identify a COS for bipolar disorder through the COMET database, the James Lind Alliance, by contacting colleagues, or from patient and clinician co-investigators.

#### Previous randomized controlled trials eligible for the MUDS study

Clinical trial sources described potential benefits (mainly symptom reduction) and hundreds of potential harms (adverse events or “side effects”) of gabapentin for neuropathic pain and quetiapine for bipolar depression. Benefits and harms were measured and reported differently. Both benefits and harms were described in public sources and non-public sources; however, non-public sources included many more potential harms than public sources. We found 19 gabapentin-neuropathic pain trials reporting outcomes in our pre-specified time-windows (e.g., 4–12 weeks), and we found 7 quetiapine-bipolar depression trials; two gabapentin trials were less than 4 weeks duration and we did not extract their outcomes following our pre-specified protocol.

Outcome domains examined for potential benefits in each case study are listed in Table 1. Examples from gabapentin-neuropathic pain RCTs are reduced pain intensity (19/19 [100%] trials), reduced sleep disturbance (11/19 [58%]), improved quality of life (7/19 [37%]), and improved mood (4/19 [21%]). Potential benefits in quetiapine-bipolar depression trials were reduced depression (7/7 [100%]), improved functioning (3/7 [43%]), improved quality of life (4/7 [57%]), reduced anxiety (5/7 [71%]), and improved sleep (1/7 [14%]). Examples of outcome domains for potential harms that were assessed systematically (i.e., planned to be recorded for all participants in an RCT) for quetiapine-bipolar depression trials were weight gain (5/7 [71%]) and suicide (6/7 [86%]). Examples of unsystematically assessed harms (i.e., events recorded only when participants reported them to providers) included dizziness, nausea, and headaches.

#### Food and Drug Administration prescribing information (package inserts)

Prescribing information for FDA-approved indications included some outcomes from clinical trials submitted to FDA for those indications, including “pain intensity” for trials of gabapentin and “depression” for trials of quetiapine. There was little information about potential benefits related to outcomes other than the labeled indications, and prescribing information did not include any potential benefits that we did not also locate in publicly available published reports of the clinical trials. For gabapentin, prescribing information did not include any information about potential benefits noted to be relevant to off-label uses included in our study (i.e., gabapentin is approved for postherpetic pain but is not approved for all types of neuropathic pain) [27].

Prescribing information included many potential harms. It was often unclear whether information about harms was applicable to any condition or only specific conditions. Prescribing information included some potential harms that we would not have identified if we had relied only on reports of clinical trials and systematic reviews. For example, prescribing information for quetiapine indicated that it might be associated with increased risk of cataract, information that came from dog studies [28].

#### DRUGDEX compendium

As noted in the “Methods” section, DRUGDEX entries were organized by intervention rather than condition. In entries for gabapentin and quetiapine, we identified information about the outcome domains “pain intensity” and “depression,” respectively. We found little information about other potential benefits of treatment for either on-label or off-label uses of the drugs. We did not identify any potential benefits or harms in DRUGDEX that we did not also identify in clinical trials or prescribing information. Similar to prescribing information, it was not always clear in DRUGDEX whether harms were associated with all conditions for which a drug might be used or only specific conditions.

#### PatientsLikeMe

Using PatientsLikeMe, we did not identify potential benefits related to our case studies other than the patient ratings of “effectiveness” for the conditions “bipolar disorder” and “neuropathic pain.” We were able to identify potential harms and the possible frequency of harms, but because the importance of each harm was not recorded, we did use PatientsLikeMe to prioritize specific harms associated with gabapentin or quetiapine. For example, some rare events, such as serious AEs, may be more important than common events.

#### Engaging patient and clinician investigators

Patient and clinician co-investigators confirmed that the outcome domains we identified using the sources of information described in the “Methods” section were important to them. In addition, they identified other important outcomes: “pain interference” (i.e., the extent to which pain interferes with other activities) for trials of neuropathic pain, “psychiatric hospitalization” for trials of bipolar depression, and “sexual functioning” for both conditions. Patient co-investigators emphasized that they wanted to know about all harms that could be associated with treatment, including those that they would not have identified themselves (e.g., blood glucose).

The two patient co-investigators in our study agreed to rate potential outcome domains by relative importance using a survey we developed for part 1. The two patients categorized many potential beneficial and harmful outcomes as “definitely analyze” (Additional file 4). Patient co-investigators also identified an important methodological problem, which we did not resolve in this project, when they said that our questions about harms were unclear because the relative importance of a given harm is related to its severity and duration.

For our overall MUDS study, we determined that we would not be able to extract data for all of the many possible outcome domains that patients classified as “definitely analyze;” thus, we selected five outcome domains for gabapentin-neuropathic pain and eight outcome domains for quetiapine-bipolar depression. Because our patient co-investigators were concerned about whether they were able to speak for others in prioritizing PCOs, we also explored other methods of prioritization in part 2.

### Part 2: prioritizing patient-centered outcomes for pain

In part 2, we invited 4130 people on the TMJ Association email list to complete a survey; 746 (18%) completed some or all of the survey. From all analyses, we excluded participants who did not provide demographic characteristics or rate their present pain intensity (*N* = 38). From the analysis of potential benefits, we also excluded 101 participants who did not rank all of the six specific benefits and the item “side effects.” From the analysis of potential harms, we excluded 110 participants who did not rank all seven potential harms. Thus, 388/746 (52%) participants were included in the analysis (Fig. 1). Descriptive information about the 388 participants we included is presented in Table 2 and Additional file 5.

**Table 2:** Baseline characteristics of part 2 survey participants included in the final analysis (*N* = 388)

Number of women (percentage)	357	(92%)
Median years of age (IQR)	52	(8)
Median years of age diagnosed with a pain disorder (IQR)^a^	30	(18)
Median PPI (IQR)	4	(3)
Median number of comorbid pain conditions (IQR)	3	(2)
Median number of current pain medications (IQR)	3	(3)
Median number of past pain medications (IQR)	6	(7)

^a^Three hundred sixty-two participants included because 26 participants did not indicate the age they were diagnosed with a pain disorder

*IQR* interquartile range (the difference between the 75th and the 25th percentile), *PPI* present pain intensity

**Fig. 2:**
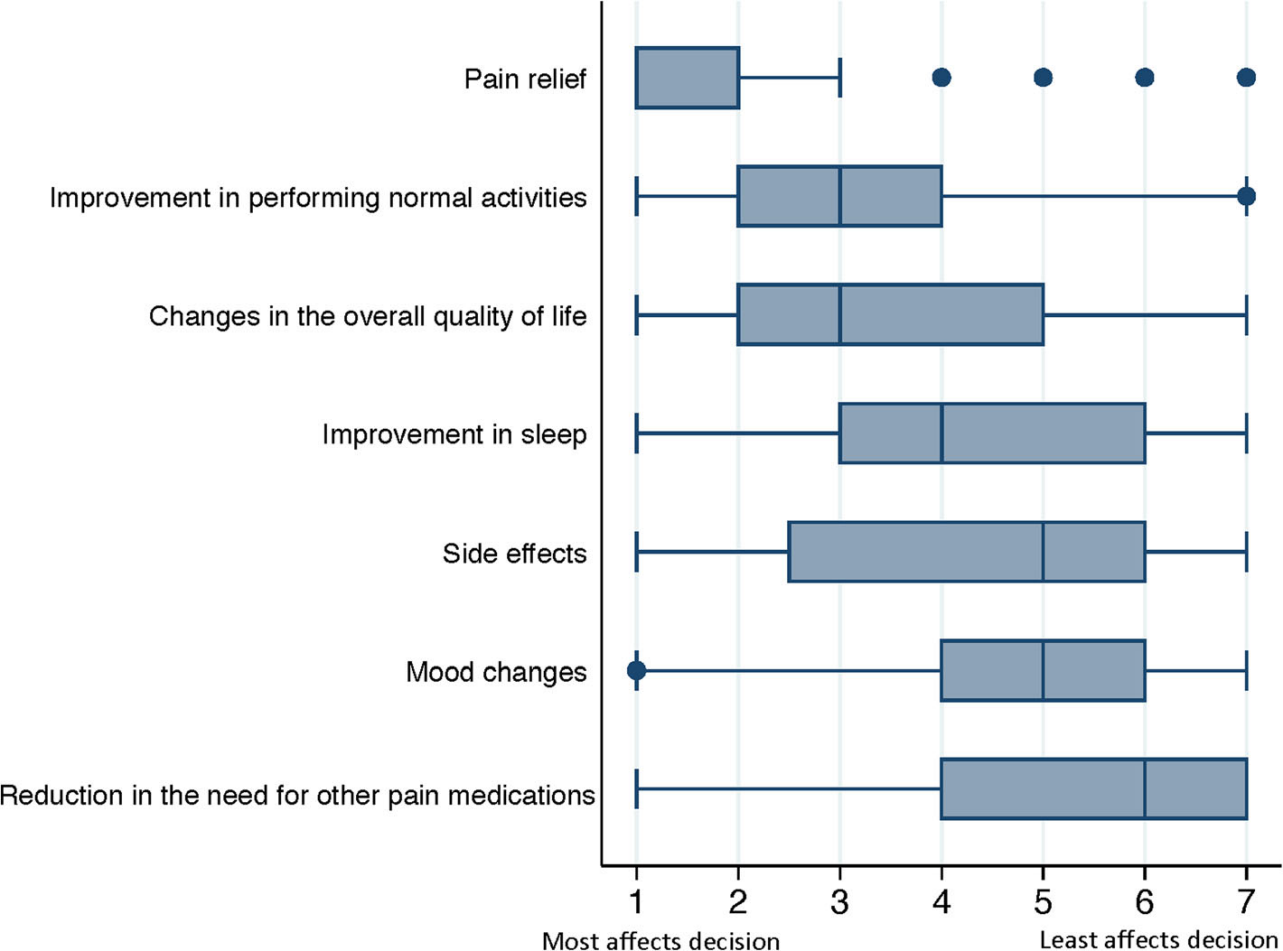
Ranking of potential beneficial outcomes and “side effects” affecting patients’ decisions to use or not to use a treatment. Considering the decision to use a treatment for pain, patients (*N* = 372) ranked the importance of six potential beneficial outcomes and the outcome domain side effects from 1 (“most affects your decision”) to 7 (“least affects your decision”). Each box plot shows the median rank and the interquartile range

Of the potential benefits that participants ranked from 1 to 7, “pain relief” was ranked as the most important (median = 1; IQR = 2); “improvement in your ability to do normal activities” and “changes in the overall quality of your life” were ranked second and third most important, respectively (Fig. 2).

Considering responses by participants who completed the open-ended question about potential benefits, 109/372 (29%) named other potential benefits they would want to know about before starting treatment (Additional file 6). For example, patients identified specific outcomes that would affect their quality of life, “I want the quality of life to improve. This could mean making me feel happier, relieving stress, reducing inflammation, and making it easier for me to have an active life.” Although our question asked about potential benefits, many patients used this section to describe the importance of avoiding specific harms, for example, “to be able to have a longer acting medication without that foggy feeling or feeling as though you could just fall asleep on the spot.”

**Fig. 3:**
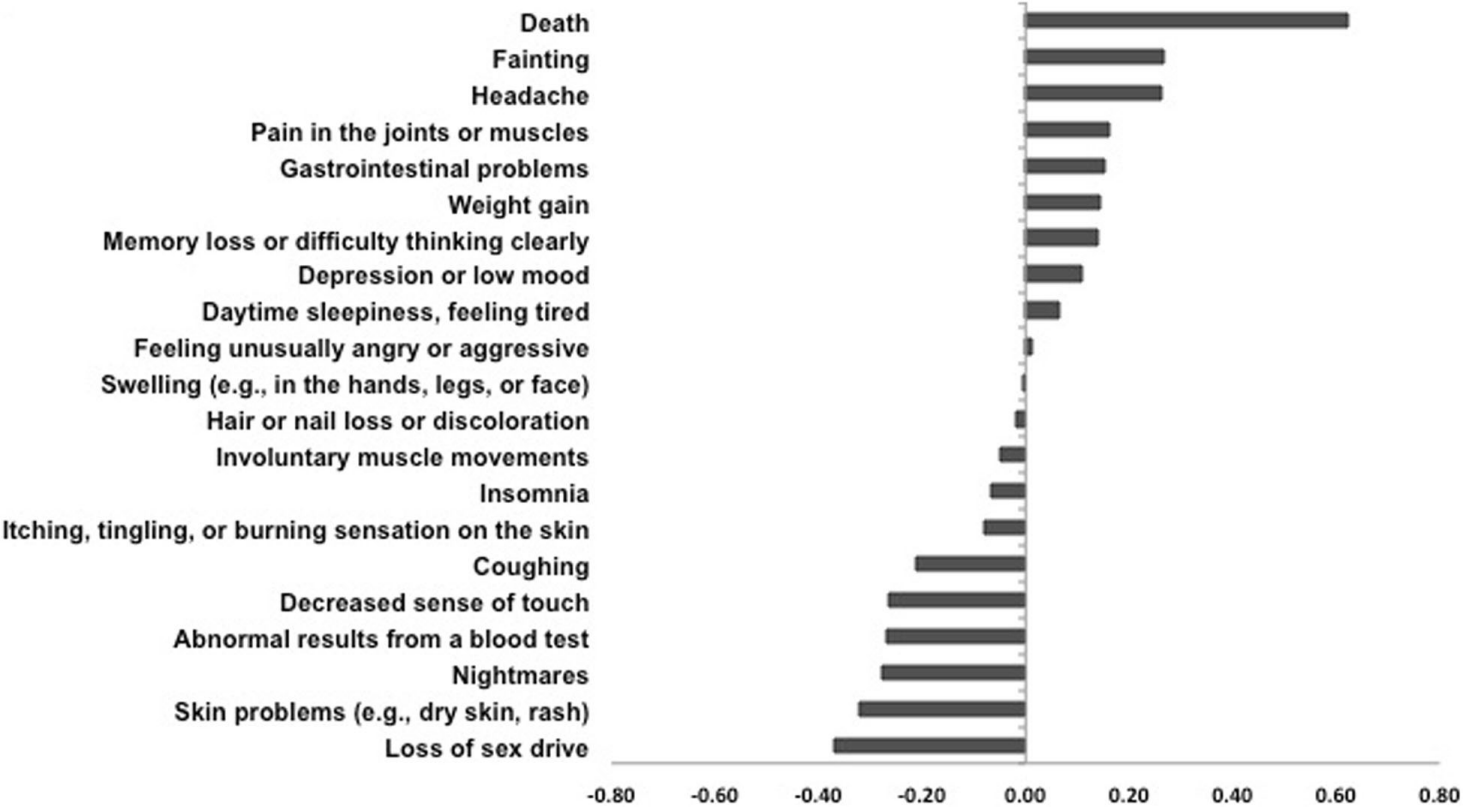
Part 2 survey respondents’ ordering of potential harmful outcome domains that would affect their decision to use a treatment. Twelve groups of survey participants were presented with 7/21 potential harms. Each participant (*N* = 363) ranked the importance of the potential harms in making a decision about whether to use a treatment for pain, and we used BWS to order them. For each harm in the set, we subtracted the proportion of participants who ranked each potential harm “1” from the proportion of participants who ranked each harm “7” to obtain a BWS score. Thus, scores greater than 0 were ranked 1 (“most affects your decision”) more often than they were ranked 7 (“least affects your decision”)

Participants in the 12 groups that ranked subsets of the 21 potential harms were similar in their age, sex, and pain (Additional file 7), suggesting that combining their scores for an overall ranking was appropriate. Of the potential harms that participants ranked from 1 to 7 in one of the 12 groups and we ranked using methods described in the “Methods” section, we found that “death” was ranked as the most important and “fainting” and “headache” followed (Fig. 3).

Considering responses by the participants who completed the open-ended question about potential harms, 245/363 (67%) named other potential harms they would want to know about before starting treatment (Additional file 6). Many of the harms that patients listed in response to this question were included in the lists of harms that were ranked by the other groups (that is, other patients ranked the importance of those harms). In addition, many patients emphasized the importance of long-term harms and interactions with other medications and 38/363 (10%) responded that they wanted to know about all potential harms. For example, “All side effects are too great a risk if they impair my ability to [perform] my job,” and “I’d want to [know about] any and all side effects that would affect my body negatively in ANY way no matter how long after I’ve taken the medication. It needs to be TRANSPARENT….”

We asked all survey participants to compare potential benefit (i.e., “the likelihood that the medication will reduce your symptoms) with potential harm (i.e., the likelihood that you will experience side effects”). Most of the 383 respondents to this question said that symptom reduction was more important when deciding whether to use a treatment (231/383; 60%); a minority said that side effects were more important (106/383; 28%) or that symptom reduction and side effects were equally important (46/383; 12%).

Comparing the (1) COS and (2) the ratings of patient co-investigators (part 1) with (3) results of the patient survey (part 2), we found that all three groups said pain intensity, physical functioning, emotional function, and adverse events were important to people with pain. The COS for pain included little information about specific harms; by contrast, patient and clinician co-investigators, and participants in the patient survey, identified many specific harms. We found that part 2 survey participants named benefits not identified using other sources, such as “able to socialize better,” “added energy,” and “to eventually stop meds––in other words, a medication that will cure!”

## Discussion

### Summary of findings

We found that various methods and sources were useful for identifying PCOs for CER, but the list of PCOs we identified was long. When we attempted to reduce the number of items on the list, we encountered challenges in prioritizing outcomes. In part 1, we identified PCOs associated with gabapentin for neuropathic pain and quetiapine for bipolar depression. Patient co-investigators said that we should include almost all of the outcomes we identified, even those not traditionally viewed as patient-centered (e.g., blood glucose). We considered conducting a systematic search of existing patient engagement research, but we found this was not feasible for our study. Thus, we undertook part 2 to prioritize the PCOs we would examine in MUDS. Predictably, by surveying many patients, we identified a range of views about the relative importance of potential benefits and potential harms, including potential benefits and harms that our patient and stakeholder co-investigators did not identify.

We found that the possible benefits of treatment could be captured in a short list, so it was relatively straightforward for patients to prioritize them. However, our patient co-investigators found it challenging to prioritize potential harms because hundreds of potential harms were associated with each drug. In addition, many patients reported that they considered all potential harms important. We are not aware of any evidence-based methods for prioritizing such a large number of PCOs. Moreover, as patient co-investigators pointed out, harms differ in type (e.g., dizziness, nausea, headache) as well as degree (e.g., mild, severe) and duration (e.g., chronic, acute); considering these differences would lead to an even longer long list.

### Implications for research

Our findings highlight several challenges for researchers. For example, we thought it would be logical to review PCO research before undertaking a new study, yet we found that a systematic search would have required more resources than we could commit to this task. Unless common terms are used to index studies about patient preferences (e.g., in databases such as PubMed), PCO research may remain difficult to locate and use for clinical research. Furthermore, we know that clinical trials and systematic reviews should assess core outcomes where possible, but we found a COS for only one of two conditions: few patients were involved in that COS, and the COS included little information about potential harms. The challenges we faced when identifying and prioritizing harms are especially important because 40% of patients surveyed in part 2 believed potential harms were as important or more important than potential benefits. Given the differences between prioritizing potential benefits and harms, these two types of outcomes might be included in separate COSs.

Using any or all the methods we used to identify PCOs could be impractical for other studies, so it is important to identify which methods are most informative and most efficient. We found our part 2 survey most useful for prioritizing outcomes, but this was possible because one of our patient co-investigators led the patient group we surveyed. Reliable COSs would limit the need for each individual trial or review to identify PCOs [29]. In the future, COSs would be especially valuable if they were to limit potential conflicts of interest (e.g., industry funding), include representative groups of patients, and identify the specific harms that are most important to patients.

### Limitations

This study has several limitations. First, the availability of sources of evidence (e.g., COSs, clinical trials) might differ for other health conditions and interventions. Second, sources other than those we examined might include useful information that we did not assess. For example, we examined the website PatientsLikeMe, which is a for-profit business that receives funding from industry; websites dedicated specifically to neuropathic pain or bipolar disorder might have included more detailed information. Third, because we surveyed members of the TMJ Association in part 2 and because temporomandibular joint and muscle disorders are not neuropathic conditions, patients included in the survey were from a different population than the patients in the clinical trials we included in the MUDS study. Fourth, we compared harms that differ in type; we did not consider the effects of differences in the severity and duration of these harms. Fifth, the outcomes we asked patients to prioritize in part 2 were limited to those selected by the investigators from the list we compiled in part 1; BWS, like other stated preference methods, allowed limited opportunities for patients to add outcomes. Our patient co-investigators and survey participants stated that all potential harms were important to them; if stated preference methods depend on researchers to identify potential PCOs, and if researchers do not ask about all possible outcomes, then stated preference methods could overlook PCOs that are important to patients. Further research is needed to compare BWS with other methods for prioritizing PCOs and to evaluate the generalizability of our results.

## Conclusions

Using several sources of information, we identified many potential benefits and harms of interest to patients taking gabapentin for neuropathic pain and quetiapine for bipolar depression. Including patients as co-investigators was valuable because patients identified important outcomes and provided ongoing feedback about the conduct of the study. While the methods used in part 1 were useful for identifying outcomes, they were less useful for prioritizing outcomes, and patient co-investigators were apprehensive about prioritizing outcomes for a large population of patients. By engaging a patient group through an exploratory survey, we were able to prioritize potential beneficial outcomes for gabapentin-neuropathic pain. Prioritizing potential harms was more complicated. Increasing the availability and use of COSs, preferably those that include meaningful patient engagement, could be an important strategy for improving the patient-centeredness of CER, [30] especially if future COS address the prioritization of potential harms.

## Additional files

Additional file 1: Search strategies. (PDF 2549 kb)

Additional file 2: Patient and clinician co-investigator survey. (PDF 597 kb)

Additional file 3: Part 2 Survey. (PDF 905 kb)

Additional file 4: Patient co-investigator responses. (PDF 2693 kb)

Additional file 5: TMJ survey participant characteristics. (PDF 1546 kb)

Additional file 6: All “other” potential benefits and harms reported by survey participants in open-ended questions. (PDF 3420 kb)

Additional file 7: Baseline characteristics of survey participants included in the final analysis by the reported month of birth. (PDF 1966 kb)

## Abbreviations

BWS: Best-worst scaling
CER: Comparative effectiveness research
CMS: Centers for Medicare and Medicaid Services
COMET: Core Outcome Measures in Effectiveness Trials
COS: Core outcome set
IMMPACT: Initiative on Methods, Measurement, and Pain Assessment in Clinical Trials
MUDS: Multiple Data Sources for Meta-Analysis
PCO: Patient-centered outcome
RCT: Randomized controlled trial
SRDR: Systematic review data repository
