# Supplementary material for: Methods to identify and prioritize patient-centered outcomes for use in comparative effectiveness research
Source: Pilot Feasibility Stud. 2018 Jun 12;4:95. doi: 10.1186/s40814-018-0284-6 (PMC6047482; doi:10.1186/s40814-018-0284-6)
Supplement: Supplementary file 1 — Search strategies. (PDF 2549 kb) [file 40814_2018_284_MOESM1_ESM.pdf]

S1: Search strategies

S1a: Gabapentin treatment for neuropathic pain

Strategy used to search Medline (OVID) for patient important outcomes used in randomized clinical trials (11,326 hits)
Search conducted September 5, 2014 by Lori Rosman

1. exp Pain Measurement/ or exp Pain/ or pain.tw.
2. exp Paresthesia/ or exp Somatosensory Disorders/ or exp Neuralgia/ or exp Polyradiculoneuropathy/ or exp Paraneoplastic Polyneuropathy/ or *Peripheral Nervous System Diseases/dt
3. (paresthesia or paraesthesia or paresthesias or paraesthesias or neuropathic pain or painful neuropathy or chronic neuropathic pain or neuropathic chronic pain).tw.
4. 1 or 2 or 3
5. exp Amputation/ or exp Amputation Stumps/ or exp Amputation, Traumatic/ or exp Central Nervous System Diseases/ or exp Diabetes Mellitus/ or exp Diabetes Mellitus, Type 2/ or exp Diabetes Mellitus, Type 1/ or exp Diabetes Complications/ or exp Guillain-Barre Syndrome/ or exp HIV Seropositivity/ or exp HIV Infections/ or exp Acquired Immunodeficiency Syndrome/ or exp Multiple Sclerosis/ or exp Neoplasms/ or exp Neoplasm Recurrence, Local/ or exp Neoplasm Metastasis/ or exp Neoplasm Invasiveness/ or exp Drug Resistance, Neoplasm/ or exp Nervous System Neoplasms/ or exp Causalgia/ or exp Complex Regional Pain Syndromes/ or exp Reflex Sympathetic Dystrophy/ or exp Phantom Limb/ or exp Trauma, Nervous System/ or exp Spinal Cord Injuries/ or exp Thoracic Injuries/ or exp Stroke/ or exp Trigeminal Neuralgia/ or exp Carpal Tunnel Syndrome/
6. (amputation or amputations or "traumatic amputation" or "traumatic amputations" or traumatic or trauma or diabetes or "diabetes mellitus" or GBS or "demyelinating polyneuropathy" or "Acute Autoimmune Neuropathy" or "acute autoimmune neuropathies" or "acute inflammatory polyneuropathy" or "acute inflammatory polyneuropathies" or "guillain barre syndrome" or "guillaine barre syndrome" or "guillainbarre syndrome" or "guillain-barre syndrome" or "guillaine-barre syndrome" or "acute inflammatory demyelinating polyneuropathy" or HIV or AIDS or "human immunodeficiency virus" or "multiple sclerosis" or tumor or tumour or cancer or malignancy or carcinoma or neoplasm or neuralgia or "phantom limb pain" or "complex regional pain syndrome" or CRPS or causalgia or "spinal cord injury" or stroke or paralysis or "reflex sympathetic dystrophy" or "trigeminal neuralgia" or "tic douloureux" or "carpal tunnel syndrome").tw.
7. 5 or 6
8. 4 and 7
9. exp Diabetic Neuropathies/ or exp Herpes Zoster/co or exp Neuralgia, Postherpetic/

10. ("diabetes complication" or "diabetes complications" or "diabetic complication" or "diabetic complications" or "painful diabetic neuropathy" or "diabetic neuralgia" or PDN or "diabetic neuropathies" or "diabetic polyneuropathy" or "diabetic polyneuropathies" or "diabetic autonomic neuropathy" or "diabetic mononeuropathy" or "diabetic mononeuropathies" or "painful diabetic neuropathies" or "diabetic amyotrophy" or herpes or shingles or zoster or HSV or "diabetic neuropathic pain" or DPN or PHN or "neuropathic cancer pain" or "neuropathic pain syndrome" or "neuropathic pain syndromes" or "chemotherapy-induced neuropathic pain" or "chemotherapy-induced peripheral neuropathy" or "chemotherapy induced neuropathic pain" or CINP).tw.
11. 9 or 10
12. 8 or 11
13. exp Amines/ or exp Cyclohexanecarboxylic Acids/ or gamma-Aminobutyric Acid/ or exp GABA Agonists/ or exp GABA Agents/ or exp Anticonvulsants/ or exp Anticonvulsants/ or exp Epilepsy/de, dt, th
14. (anticonvulsant or "anticonvulsants anti-convulsant" or "anti-convulsants" or "antiepileptic drug" or "antiepileptic drugs" or "anti-epileptic drug" or "anti-epileptic drugs" or "antiseizure drug" or "antiseizure drugs" or "anti-seizure agent" or "antiepileptic agent" or "antiepileptic agents" or "anti-epileptic agent" or "anti-epileptic agents" or gabapentin or Neurontin).tw.
15. 13 or 14
16. 12 and 15
17. Consumer organizations/
18. 12 and 17
19. exp Qualitative Research/ or Nursing Methodology Research/ or Delphi Technique/ or exp Focus Groups/ or exp Interviews as Topic/ or Narration/ or Autobiography as Topic/ or Consensus/ or exp Congresses as Topic/ or self disclosure/ or disclosure/ or truth disclosure/ or exp Consumer Participation/
20. (deliberative forum* or autoethnograph* or ethnograph* or phenomenol* or autobiograph* or "open ended" or grounded theor* or ((content or discourse or framework) and analys*) or delphi or mixed method* or consensus or focus group* or theme or themes or thematic or structured interview* or unstructured interview* or needs assess*).tw.
21. 19 or 20
22. 12 and 21
23. Patient Outcome Assessment/ or health status indicators/ or exp Personal Satisfaction/ or self report/ or Self-Assessment/ or "Outcome and Process Assessment (Health Care)"/ or "Outcome Assessment (Health Care)"/ or patient advocacy/
24. (PROMS or CORE outcome* or "living with").tw.
25. ((active or routine* or regular*) adj2 (feedback or measurement* or monitor*)).tw.
26. (outcome* adj3 (feedback or manag* or monitor*)).tw.
27. 23 or 24 or 25 or 26

28. 12 and 27

29. (interview* or qualitative* or survey or surveys or surveyed).tw.

30. Self concept/

31. ((patient* or self or client* or subject* or participant* or lived or personal or consumer* or service user* or family or carer or carers or caregiver* or parent* or father* or mother* or spouse* or husband* or wife or wives* or child* or sibling* or brother* or sister* or relative*) adj5 (report* or relate* or view* or expectation* or perception* or perspective* or experience* or described or narrative or narration or outcome* or measure* or assess* or monitor* or symptom* or domain* or burden* or impact* or effect*)).tw.

32. 30 or 31

33. 12 and 29 and 32

34. 18 or 22 or 28 or 33

S1b: Quetiapine treatment for bipolar depression

Strategy used to search Ovid-Medline for patient important outcomes used in randomized clinical trials (7,428 hits)

Searches conducted September 30, 2014 by Lori Rosman

1. exp Bipolar Disorder/ or Mood Disorders/ or (Bipolar or "bi polar" or hypomani* or mania* or manic* or mixed episode* or RCBD or Cyclophren* or cyclothymi* or mixed depression* or "maniodepressive" or "mano depressive").tw. or ((rapid or ultradian) adj5 cycl*).tw. or ((affective* or mood) adj (disorder* or disturbance* or dysfunction* or illness* or swing*)).tw.
2. Consumer organizations/
3. ("national alliance on mental illness" or "depression and bipolar support alliance" or "the balanced mind foundation" or "international bipolar foundation" or "international society for affective disorders" or "rethink.org" or "International Society for Bipolar Disorders").mp. [mp=title, abstract, original title, name of substance word, subject heading word, keyword heading word, protocol supplementary concept word, rare disease supplementary concept word, unique identifier]
4. 1 and 2
5. exp Qualitative Research/ or Nursing Methodology Research/ or Delphi Technique/ or exp Focus Groups/ or exp Interviews as Topic/ or Narration/ or Autobiography as Topic/ or Consensus/ or exp Congresses as Topic/ or self disclosure/ or disclosure/ or truth disclosure/ or exp Consumer Participation/
6. (deliberative forum* or autoethnograph* or ethnograph* or phenomenol* or autobiograph* or "open ended" or grounded theor* or ((content or discourse or framework) and analys*) or delphi or mixed method* or consensus or focus group* or theme or themes or thematic or structured interview* or unstructured interview* or needs assess*).tw.
7. 5 or 6
8. 1 and 7
9. Patient Outcome Assessment/ or health status indicators/ or exp Personal Satisfaction/ or self report/ or Self-Assessment/ or "Outcome and Process Assessment (Health Care)"/ or "Outcome Assessment (Health Care)"/ or patient advocacy/
10. (PROMS or CORE outcome* or "living with bipolar").tw.
11. ((active or routine* or regular*) adj2 (feedback or measurement* or monitor*)).tw.
12. (outcome* adj3 (feedback or manag* or monitor*)).tw.
13. 9 or 10 or 11 or 12
14. 1 and 13
15. (interview* or qualitative* or survey or surveys or surveyed).tw.
16. Self concept/

17. ((patient* or self or client* or subject* or participant* or lived or personal or consumer* or service user* or family or carer or carers or caregiver* or parent* or father* or mother* or spouse* or husband* or wife or wives* or child* or sibling* or brother* or sister* or relative*) adj5 (report* or relate* or view* or expectation* or perception* or perspective* or experience* or described or narrative or narration or outcome* or measure* or assess* or monitor* or symptom* or domain* or burden* or impact* or effect*)).tw.

18. 16 or 17

19. 1 and 15 and 18

20. 3 or 4 or 8 or 14 or 19

Search of Embase.com (15,545 hits)

Bipolar Concept

#1 'bipolar disorder'/exp OR 'mania'/de OR 'mood disorder'/de

#2 bipolar:ab,ti OR 'bi polar':ab,ti OR hypomani*:ab,ti OR mania*:ab,ti OR manic*:ab,ti OR rcbd:ab,ti OR cyclophren*:ab,ti OR cyclothymi*:ab,ti OR 'maniodepressive':ab,ti OR 'mano depressive':ab,ti OR (mixed NEAR/1 episode*):ab,ti

#3 ((rapid OR ultradian) NEAR/5 cycl*):ab,ti

#4 ((affective* OR mood) NEAR/1 (disorder* OR disturbance* OR dysfunction* OR illness* OR swing*)):ab,ti

#5 #1 OR #2 OR #3 OR #4

6. 'consumer'/exp

7. ("national alliance on mental illness" OR "depression and bipolar support alliance" OR "the balanced mind foundation" OR "international bipolar foundation" OR "international society for affective disorders" OR "rethink.org" or "International Society for Bipolar Disorders")

8. 'qualitative research'/exp or 'nursing methodology research'/exp or 'Delphi study'/exp or 'content analysis'/exp or 'discourse analysis'/exp or 'thematic analysis'/exp or 'semi structured interview'/exp or 'structured interview'/exp or 'telephone interview'/exp or 'unstructured interview'/exp or 'conversation'/exp or 'narrative'/exp or 'consensus'/exp or 'consensus development'/exp or 'self disclosure'/exp or 'ethnography'/exp or 'open ended questionnaire'/exp or 'grounded theory'/exp or 'qualitative analysis'/exp

9. ((deliberative NEXT/1 forum*) or autoethnograph* or ethnograph* or phenomenol* or autobiograph* or "open ended" or "grounded theory" or (content NEXT/1 analys*) or (discourse NEXT/1 analys*) or (framework NEXT/1 analys*) or delphi or (mixed NEXT/1 method*) or consensus or (focus NEXT/1 group*) or theme or themes or thematic or (structured NEXT/1 interview*) or (unstructured NEXT/1 interview*) or (needs NEXT/1 assess*)):ti,ab

10. 'outcome assessment'/exp or 'health status indicator'/de or 'satisfaction'/de or 'patient satisfaction'/exp or 'self report'/exp or 'self evaluation'/exp or 'outcomes research'/exp or 'patient advocacy'/exp or 'consumer advocacy'/exp

11. (PROMS or (CORE NEAR/1 outcome*) or "living with bipolar"):ti,ab

12. ((active or routine* or regular*) NEAR/2 (feedback or measurement* or monitor*)):ti,ab

13. (outcome* NEAR/3 (feedback or manag* or monitor*)):ti,ab

14. (interview* or qualitative* or survey or surveys or surveyed):ti,ab

15. 'self concept'/exp

16. ((patient* OR self OR client* OR subject* OR participant* OR lived OR personal OR consumer* OR "service user" OR "service users" or family or carer or carers or caregiver* or parent* or father* or mother* or spouse* or husband* or wife or wives* or child* or sibling* or brother* or sister* or relative*) **NEAR/5** (report* or relate* or view* or

expectation* or perception* or perspective* or experience* or described or narrative or narration or outcome* or measure* or assess* or monitor* or symptom* or domain* or burden* or impact* or effect*)):ti,ab

17. 6 or 7 or 8 or 9 or 10 or 11 or 12 or 13
18. 14 and (15 or 16)
19. 17 or 18
20. 5 and 19

Search of CENTRAL (1,345 hits)

#1 MeSH descriptor: [Bipolar Disorder] explode all trees

#2 MeSH descriptor: [Mood Disorders] explode all trees

#3 (Bipolar or "bi polar")

#4 ((rapid or ultradian) near/5 cycl*)

#5 (cyclothymic* or hypomani* or mania* or manic* or (mixed next episode*) or RCBD)

#6 ((affective* or mood) near/1 (disorder* or disturbance* or dysfunction* or illness* or swing*))

#7 {or #1-#6}

#8 MeSH descriptor: [Consumer Organizations] this term only

#9 ("national alliance on mental illness" or "depression and bipolar support alliance" or "the balanced mind foundation" or "international bipolar foundation" or "international society for affective disorders" or "rethink.org" or "International Society for Bipolar Disorders")

#10 #7 and #8

#11 MeSH descriptor: [Nursing Methodology Research] this term only

#12 MeSH descriptor: [Delphi Technique] this term only

#13 MeSH descriptor: [Focus Groups] explode all trees

#14 MeSH descriptor: [Interviews as Topic] explode all trees

#15 MeSH descriptor: [Narration] this term only

#16 MeSH descriptor: [Autobiography as Topic] this term only

#17 MeSH descriptor: [Consensus] this term only

#18 MeSH descriptor: [Congresses as Topic] explode all trees

#19 MeSH descriptor: [Self Disclosure] this term only

#20 MeSH descriptor: [Disclosure] this term only

#21 MeSH descriptor: [Truth Disclosure] this term only

#22 MeSH descriptor: [Consumer Participation] explode all trees

#23 ((deliberative next forum*) or autoethnograph* or ethnograph* or phenomenol* or autobiograph* or "open ended" or (grounded next theor*) or ((content or discourse or framework) and analys*) or delphi or (mixed next method*) or consensus or (focus next group*) or theme or themes or thematic or (structured next interview*) or (unstructured next interview*) or (needs next assess*))

#24 {or #11-#23}

#25 #7 and #24

#26 MeSH descriptor: [Patient Outcome Assessment] this term only

#27 MeSH descriptor: [Health Status Indicators] this term only

#28 MeSH descriptor: [Personal Satisfaction] explode all trees

#29 MeSH descriptor: [Self Report] this term only

#30 MeSH descriptor: [Self-Assessment] this term only

#31 MeSH descriptor: [Outcome and Process Assessment (Health Care)] this term only

#32 MeSH descriptor: [Outcome Assessment (Health Care)] this term only

#33 MeSH descriptor: [Patient Advocacy] this term only

#34 (PROMS or (CORE next outcome*) or "living with bipolar")

#35 ((active or routine* or regular*) near/2 (feedback or measurement* or monitor*))

#36 (outcome* near/3 (feedback or manag* or monitor*))

#37 {or #26-#36}

#38 #7 and #37

#39 (interview* or qualitative* or survey or surveys or surveyed)

#40 MeSH descriptor: [Self Concept] this term only

#41 ((patient* or self or client* or subject* or participant* or lived or personal or consumer* or (service next user*) or family or carer or carers or caregiver* or parent* or father* or mother* or spouse* or husband* or wife or wives* or child* or sibling* or brother* or sister* or relative*) near/5 (report* or relate* or view* or expectation* or perception* or perspective* or experience* or described or narrative or narration or outcome* or measure* or assess* or monitor* or symptom* or domain* or burden* or impact* or effect*))

#42 #40 or #41

#43 #7 and #39 and #42

#44 #9 or #10 or #25 or #38 or #43

Search of PSYCINFO (EBSCO) (8,382 hits)

#1 DE "Affective Disorders" OR DE "Bipolar Disorder" OR DE "Cyclothymic Personality" OR DE "Mania" OR DE "Hypomania"

#2 TI (Bipolar OR "bi polar" OR hypomani* OR mania* OR manic* OR (mixed N1 episode*) OR RCBD OR Cyclophren* OR cyclothymi* OR (mixed N1 depression*) OR "maniodepressive" OR "mano depressive") OR AB (Bipolar OR "bi polar" OR hypomani* OR mania* OR manic* OR (mixed N1 episode*) OR RCBD OR Cyclophren* OR cyclothymi* OR (mixed N1 depression*) OR "maniodepressive" OR "mano depressive") OR TM (Bipolar OR "bi polar" OR hypomani* OR mania* OR manic* OR (mixed N1 episode*) OR RCBD OR Cyclophren* OR cyclothymi* OR (mixed N1 depression*) OR "maniodepressive" OR "mano depressive")

#3 TI ((rapid OR ultradian) N5 cycl*) OR AB ((rapid OR ultradian) N5 cycl*) OR TM ((rapid OR ultradian) AND cycl*)

#4 TI ((affective* OR mood) N1 (disorder* OR disturbance* OR dysfunction* OR illness* OR swing*)) OR AB ((affective* OR mood) N1 (disorder* OR disturbance* OR dysfunction* OR illness* OR swing*)) OR TM ((affective* OR mood) N1 (disorder* OR disturbance* OR dysfunction* OR illness* OR swing*))

#5 S1 OR S2 OR S3 OR S4

#6 DE "Organizations"

#7 ("national alliance on mental illness" or "depression and bipolar support alliance" or "the balanced mind foundation" or "international bipolar foundation" or "international society for affective disorders" or "rethink.org" or "International Society for Bipolar Disorders")

#8 S5 AND S6

#9 DE "Qualitative Research" OR DE "Interviewing" OR DE "Narratives" OR DE "Narrative Therapy" OR DE "Autobiography" OR DE "Self Disclosure" OR DE "Client Participation" OR DE "Ethnography" OR DE "Phenomenology" OR DE "Grounded Theory" OR DE "Content Analysis" OR DE "Discourse Analysis" OR DE structured clinical interview OR DE "Needs Assessment" OR DE "Interviews" OR DE "Intake Interview" OR DE "Interview Schedules" OR DE "Job Applicant Interviews" OR DE "Psychodiagnostic Interview")

#10 TI ((deliberative N1 forum*) OR autoethnograph* OR ethnograph* OR phenomenol* OR autobiograph* OR "open ended" OR (grounded N1 theor*) OR ((content OR discourse OR framework) AND analys*) OR delphi OR (mixed N1 method*) OR consensus OR (focus N1 group*) OR theme OR themes OR thematic OR (structured N1 interview*) OR (unstructured N1 interview*) OR (needs N1 assess*)) OR AB ((deliberative N1 forum*) OR autoethnograph* OR ethnograph* OR phenomenol* OR autobiograph* OR "open ended" OR (grounded N1 theor*) OR ((content OR discourse OR framework) AND analys*) OR delphi OR (mixed N1 method*) OR consensus OR (focus N1 group*) OR theme OR themes OR thematic OR (structured N1 interview*) OR (unstructured N1 interview*) OR (needs N1 assess*))

#11 #9 OR #10

#12 #5 AND #11

#13 DE "Consumer Satisfaction" OR DE "Client Satisfaction" OR DE "Self Report" OR DE "Self Evaluation" OR DE "Advocacy" OR DE "Client Attitudes" OR DE "Consumer Attitudes"

#14 TI (PROMS OR (CORE N1 Outcome*) OR "living with bipolar") OR AB (PROMS OR (CORE N1 Outcome*) OR "living with bipolar")

#15 TI ((active OR routine* OR regular*) N2 (feedback OR measurement* OR monitor*)) OR AB ((active OR routine* OR regular*) N2 (feedback OR measurement* OR monitor*))

#16 TI (outcome* N3 (feedback OR manag* OR monitor*)) OR AB (outcome* N3 (feedback OR manag* OR monitor*))

#17 #13 OR #14 OR #15 OR #16

#18 #5 AND #17

#19 TI (interview* or qualitative* or survey or surveys or surveyed) OR AB (interview* or qualitative* or survey or surveys or surveyed)

#20 DE "Self Concept" OR DE "Academic Self Concept" OR DE "Self Confidence" OR DE "Self Esteem" OR DE "Self Perception"

#21 TI (patient* or self or client* or subject* or participant* or lived or personal or consumer* or (service N1 user*) or family or carer or carers or caregiver* or parent* or father* or mother* or spouse* or husband* or wife or wives* or child* or sibling* or brother* or sister* or relative*) N5 (report* or relate* or view* or expectation* or perception* or perspective* or experience* or described or narrative or narration or outcome* or measure* or assess* or monitor* or symptom* or domain* or burden* or impact* or effect*)) OR AB (patient* or self or client* or subject* or participant* or lived or personal or consumer* or (service N1 user*) or family or carer or carers or caregiver* or parent* or father* or mother* or spouse* or husband* or wife or wives* or child* or sibling* or brother* or sister* or relative*) N5 (report* or relate* or view* or expectation* or perception* or perspective* or experience* or described or narrative or narration or outcome* or measure* or assess* or monitor* or symptom* or domain* or burden* or impact* or effect*))

#22 #20 OR #21

#23 #19 AND #22 AND #5

#24 #7 OR #8 OR #12 OR #18 OR #23

Search of CINAHL (EBSCO) (3,383 hits)

#1 (MH "Bipolar Disorder+") OR (MH "Affective Disorders") OR (MH "Affective Disorders, Psychotic")

#2 TI (Bipolar OR "bi polar" OR hypomani* OR mania* OR manic* OR mixed episode* OR RCBD OR Cyclophren* OR cyclothymi* OR mixed depression* OR "maniodepressive" OR "mano depressive") OR AB (Bipolar OR "bi polar" OR hypomani* OR mania* OR manic* OR mixed episode* OR RCBD OR Cyclophren* OR cyclothymi* OR mixed depression* OR "maniodepressive" OR "mano depressive")

#3 TI ((rapid OR ultradian) N5 cycl*) OR AB ((rapid OR ultradian) N5 cycl*)

#4 TI ((affective* OR mood) N1 (disorder* OR disturbance* OR dysfunction* OR illness* OR swing*)) OR AB ((affective* OR mood) N1 (disorder* OR disturbance* OR dysfunction* OR illness* OR swing*))

#5 S1 OR S2 OR S3 OR S4

#6 (MH "Organizations+")

#7 ("national alliance on mental illness" or "depression and bipolar support alliance" or "the balanced mind foundation" or "international bipolar foundation" or "international society for affective disorders" or "rethink.org" or "International Society for Bipolar Disorders")

#8 S5 AND S6

#9 MH "Qualitative Studies+" OR MH "Delphi Technique" OR MH "Focus Groups" OR MH "Interviews+" OR MH "Narratives" OR MH "Autobiographies" OR MH "Consensual Validity" OR MH "Congresses and Conferences" OR MH "Self Disclosure" OR MH "Truth Disclosure" OR MH "Consumer Participation" OR MH "Ethnographic Research" OR MH "Phenomenology" OR MH "Phenomenological Research" OR MH "Open-Ended Questionnaires" OR MH "Grounded Theory" OR MH "Content Analysis" OR MH "Discourse Analysis" OR MH "Conceptual Framework" OR MH "Delphi Technique" OR MH "Thematic Analysis" OR MH "Structured Interview" OR MH "Semi-Structured Interview" OR MH "Unstructured Interview" OR MH "Needs Assessment"

#10 TI ((deliberative N1 forum*) OR autoethnograph* OR ethnograph* OR phenomenol* OR autobiograph* OR "open ended" OR (grounded N1 theor*) OR ((content OR discourse OR framework) AND analys*) OR delphi OR (mixed N1 method*) OR consensus OR (focus N1 group*) OR theme OR themes OR thematic OR (structured N1 interview*) OR (unstructured N1 interview*) OR (needs N1 assess*)) OR AB ((deliberative N1 forum*) OR autoethnograph* OR ethnograph* OR phenomenol* OR autobiograph* OR "open ended" OR (grounded N1 theor*) OR ((content OR discourse OR framework) AND analys*) OR delphi OR (mixed N1 method*) OR consensus OR (focus N1 group*) OR theme OR themes OR thematic OR (structured N1 interview*) OR (unstructured N1 interview*) OR (needs N1 assess*))

#11 #9 OR #10

#12 #5 AND #11

#13 (MH "Outcome Assessment") OR (MH "Health Status Indicators") OR (MH "Personal Satisfaction") OR (MH "Self Report") OR (MH "Self Assessment") OR (MH "Process Assessment (Health Care)") OR (MH "Patient Advocacy")

#14 TI (PROMS OR (CORE N1 Outcome*) OR "living with bipolar") OR AB (PROMS OR (CORE N1 Outcome*) OR "living with bipolar")

#15 TI ((active OR routine* OR regular*) N2 (feedback OR measurement* OR monitor*)) OR AB ((active OR routine* OR regular*) N2 (feedback OR measurement* OR monitor*))

#16 TI (outcome* N3 (feedback OR manag* OR monitor*)) OR AB (outcome* N3 (feedback OR manag* OR monitor*))

#17 #13 OR #14 OR #15 OR #16

#18 #5 AND #17

#19 TI (interview* or qualitative* or survey or surveys or surveyed) OR AB (interview* or qualitative* or survey or surveys or surveyed)

#20 (MH "Self Concept+")

#21 TI (patient* or self or client* or subject* or participant* or lived or personal or consumer* or (service N1 user*) or family or carer or carers or caregiver* or parent* or father* or mother* or spouse* or husband* or wife or wives* or child* or sibling* or brother* or sister* or relative*) N5 (report* or relate* or view* or expectation* or perception* or perspective* or experience* or described or narrative or narration or outcome* or measure* or assess* or monitor* or symptom* or domain* or burden* or impact* or effect*)) OR AB (patient* or self or client* or subject* or participant* or lived or personal or consumer* or (service N1 user*) or family or carer or carers or caregiver* or parent* or father* or mother* or spouse* or husband* or wife or wives* or child* or sibling* or brother* or sister* or relative*) N5 (report* or relate* or view* or expectation* or perception* or perspective* or experience* or described or narrative or narration or outcome* or measure* or assess* or monitor* or symptom* or domain* or burden* or impact* or effect*))

#22 #20 OR #21

#23 #19 AND #22 AND #5

#24 #7 OR #8 OR #12 OR #18 OR #23

Search of PubMed (excluding Medline records to retrieve records not currently in Medline (OVID) (1,373 hits)

1. (Bipolar[tw] OR "bi polar"[tw]) NOT Medline[sb]
2. ((rapid[tw] OR ultradian[tw]) AND cycl*[tw]) NOT Medline[sb]
3. (hypomani*[tw] OR mania*[tw] OR manic*[tw] OR mixed episode*[tw] OR RCBD[tw] OR Cyclophren*[tw] OR cyclothymi*[tw] OR mixed depression*[tw] OR "maniodepressive"[tw] OR "mano depressive"[tw]) NOT Medline[sb]
4. ((affective*[tw] OR mood[tw]) AND (disorder*[tw] OR disturbance*[tw] OR dysfunction*[tw] OR illness*[tw] OR swing*[tw])) NOT Medline[sb]
5. #1 OR #2 OR #3 OR #4
6. ("national alliance on mental illness" or "depression and bipolar support alliance" or "the balanced mind foundation" or "international bipolar foundation" or "international society for affective disorders" or "rethink.org" or "International Society for Bipolar Disorders") NOT Medline[sb]
7. (deliberative forum*[tw] OR autoethnograph*[tw] OR ethnograph*[tw] OR phenomenol*[tw] OR autobiograph*[tw] OR "open ended"[tw] OR grounded theor*[tw] OR ((content[tw] OR discourse[tw] OR framework[tw]) AND analys*[tw]) OR delphi[tw] OR mixed method*[tw] OR consensus[tw] OR focus group*[tw] OR theme[tw] OR themes[tw] OR thematic[tw] OR structured interview*[tw] OR unstructured interview*[tw] OR needs assess*[tw]) NOT Medline[sb]
8. (PROMS[tw] OR CORE outcome*[tw] OR "living with bipolar"[tw]) NOT Medline[sb]
9. ((active[tw] OR routine*[tw] OR regular*[tw]) AND (feedback[tw] OR measurement*[tw] OR monitor*[tw])) NOT Medline[sb]
10. (outcome*[tw] AND (feedback[tw] OR manag*[tw] OR monitor*[tw])) NOT Medline[sb]
11. #8 OR #9 OR #10
12. #5 AND #11
12. (interview*[tw] OR qualitative*[tw] OR survey[tw] OR surveys[tw] OR surveyed[tw]) NOT Medline[sb]
13. ((patient*[tw] OR self[tw] OR client*[tw] OR subject*[tw] OR participant*[tw] OR lived[tw] OR personal[tw] OR consumer*[tw] OR service user*[tw] OR family[tw] OR carer[tw] OR carers[tw] OR caregiver*[tw] OR parent*[tw] OR father*[tw] OR mother*[tw] OR spouse*[tw] OR husband*[tw] OR wife[tw] OR wives*[tw] OR child*[tw] OR sibling*[tw] OR brother*[tw] OR sister*[tw] OR relative*[tw]) AND (report*[tw] OR relate*[tw] OR view*[tw] OR expectation*[tw] OR perception*[tw] OR perspective*[tw] OR experience*[tw] OR described[tw] OR narrative[tw] OR narration[tw] OR outcome*[tw] OR measure*[tw] OR assess*[tw] OR monitor*[tw] OR symptom*[tw] OR domain*[tw] OR burden*[tw] OR impact*[tw] OR effect*[tw])) NOT Medline[sb]
14. #12 AND #13
15. #5 AND #14
16. #6 OR #12 OR #15

S2: Patient and clinician co-investigator survey

MUDS Team,
In anticipation of our investigators meeting, we would like to get a better idea of what the team thinks are the most important outcomes for gabapentin and quetiapine.

As a starting point, we have created a list of outcomes that we think may be important to patients related to these medications.

Please let us know how important you think these outcomes are and add in new patient-centered outcomes at the bottom.

Thanks,
Evan and the MUDS team

1. Please rate the importance of analyzing each outcome in the gabapentin analyses, when data is available, during the MUDS study.

	Definitely analyze, very important to patients	Possibly analyze, sometimes important to patients or important to some patients	Definitely do NOT analyze, not important to patients	Do not understand or know what this is
Number of responders (participants reporting clinically important reduction in daily pain intensity)	◯	◯	◯	◯
Change in pain intensity (mean difference from baseline)	◯	◯	◯	◯
Serious adverse events (number of each event)	◯	◯	◯	◯
Number of participants with self-reported improvement	◯	◯	◯	◯
Number of participants with clinician-reported improvement	◯	◯	◯	◯
Depression (self-reported)	◯	◯	◯	◯
Anxiety (self-reported)	◯	◯	◯	◯
Pain affect (Short Form-McGill Pain Questionnaire)	◯	◯	◯	◯
Sleep interferences (daily rating)	◯	◯	◯	◯
Health-related quality of life	◯	◯	◯	◯
Discontinuation for any reason (number of participants who discontinued the drug)	◯	◯	◯	◯
Participants experiencing one or more side effects (number of participants)	◯	◯	◯	◯
Discontinuation because of side effects (number of participants)	◯	◯	◯	◯
Harm:benefit analysis	◯	◯	◯	◯
Cognitive dysfunction	◯	◯	◯	◯
Memory impairment	◯	◯	◯	◯
Fatigue	◯	◯	◯	◯
Vertigo	◯	◯	◯	◯
Dizziness	◯	◯	◯	◯
Nausea	◯	◯	◯	◯
Constipation	◯	◯	◯	◯
Vomiting	◯	◯	◯	◯
	◯	◯	◯	◯

Pain interference	◯	◯	◯	◯
Physical activity	◯	◯	◯	◯
Sleep	◯	◯	◯	◯
Emotional functioning/mood	◯	◯	◯	◯
Sleep interference scores/sleep difficulties	◯	◯	◯	◯
Quality of life daily pain score measured on an 11-point Likert scale	◯	◯	◯	◯
Pain severity score	◯	◯	◯	◯
Mean total SF-MPQ pain Scores	◯	◯	◯	◯
Dizziness	◯	◯	◯	◯
Somnolence	◯	◯	◯	◯
Confusion	◯	◯	◯	◯

List important outcomes that are important to patients missing from the above list. Only list the items that you feel should DEFINITELY be analyzed.

2. Please rate the importance of analyzing each outcome in the quetiapine analyses, when data is available, during the MUDS study.

	Definitely analyze, very important to patients	Possibly analyze, sometimes important to patients or important to some patients	Definitely do NOT analyze, not important to patients	Do not understand or know what this is
Number of responders (participants reporting clinically important reduction in depression rating)	◯	◯	◯	◯
Change in depression (mean difference from baseline)	◯	◯	◯	◯
Discontinuation for any reason (number of participants who discontinued the drug)	◯	◯	◯	◯
Serious adverse events (number of each event)	◯	◯	◯	◯
Number of remitters (participants scoring below the cut-off for a clinical episode)	◯	◯	◯	◯
Functioning (mean score on the Global Assessment of Functioning scale)	◯	◯	◯	◯
Health-related quality of life	◯	◯	◯	◯
Change in anxiety (mean difference from baseline on a validated scale)	◯	◯	◯	◯
Hospitalization (number of participants hospitalized)	◯	◯	◯	◯
Suicide	◯	◯	◯	◯
Participants experiencing one or more side effects (number of participants)	◯	◯	◯	◯
Discontinuation because of side effects (number of participants)	◯	◯	◯	◯
Specific side effects (number of each side effect organized using standard classifications)	◯	◯	◯	◯
Change in fasting glucose level	◯	◯	◯	◯
Change in triglycerides	◯	◯	◯	◯
Diabetes	◯	◯	◯	◯
Change in weight	◯	◯	◯	◯
Cardiovascular effects (change in QTc interval duration, incidence of orthostatic hypotension)	◯	◯	◯	◯
Extrapyramidal symptoms (tardive dyskinesia, dystonia, akathisia)	◯	◯	◯	◯
Participants experiencing any extrapyramidal symptoms	◯	◯	◯	◯
Mean score for measures of extrapyramidal symptoms (e.g. Abnormal Involuntary Movement Scale, Condensed User's Scale, Simpson-Angus Scale, Barnes Akathesia Rating Scale, or similar scale)	◯	◯	◯	◯
Mean change in serum prolactin levels	◯	◯	◯	◯
Hematologic effects (incidence of absolute neutrophil count (ANC) < 100/ml (count data/rate)	◯	◯	◯	◯
Weight gain	◯	◯	◯	◯
Symptoms related to daytime drowsiness	◯	◯	◯	◯
Loss of energy	◯	◯	◯	◯
Inability to concentrate	◯	◯	◯	◯

List important outcomes that are important to patients missing from the above list. Only list the items that you feel should DEFINITELY be analyzed.

3. If you are aware of any publications that have identified outcomes that are important to patients with pain, bipolar disorder, gabapentin or quetiapine, please paste the citations below.

***4. Please enter your initials below so we know who has completed the survey.**

If you have questions about the survey contact Susie at shutfle1@jhmi.edu

Thanks!
Evan and the MUDS team

S3: Part 2 Survey

Page 1

Thank you for your interest in participating in this survey.

By entering information into this survey, you are providing your consent for investigators at Johns Hopkins to use the information for research. The survey does NOT ask for your name or for any personally identifiable information, and the researchers will not attempt to identify you.

*1. You must be 18 years or older to participate in this survey.

Are you 18 years or older?

People with pain have many different treatment options. Different treatments have different benefits and side effects that might affect your willingness to choose a drug or continue taking a drug. Benefits are the ways that a drug might make you feel better or improve your life. Side effects are undesirable or harmful effects of a drug.

We want to know which benefits and side effects matter the most to you.

To help us understand your point of view, we will ask about which treatments you have used and some other background information about you.

1. You will see questions based on the month that you were born in.

In which month were you born?

Benefits January

1. You might like to know about the possibility of certain benefits and side effects because they affect your decision about which treatment to take.

Please rank the importance of the following as they relate to your decision to use or not use a treatment. Put the options in order from 1 to 7 such that:

1 MOST affects your decision
7 LEAST affects your decision

You can use the drop-down boxes to select your choices or drag the rows into your preferred order.

☐	Pain relief (reduced intensity or severity)
☐	Improvement in your ability to do normal activities (social activities, work, school, family life)
☐	Improvement in sleep
☐	Changes in the overall quality of your life
☐	Changes in mood (for example, feeling less anxious or depressed)
☐	Reductions in your need for other pain medication
☐	Side effects

2. Are there other ways you might want a medication for pain to improve your health or your life (that is, other benefits you're seeking from treatment)?

○ Yes

○ No

If yes, what are these benefits?

Benefits February

1. You might like to know about the possibility of certain benefits and side effects because they affect your decision about which treatment to take.

Please rank the importance of the following as they relate to your decision to use or not use a treatment. Put the options in order from 1 to 7 such that:

1 MOST affects your decision
7 LEAST affects your decision

You can use the drop-down boxes to select your choices or drag the rows into your preferred order.

☐	Pain relief (reduced intensity or severity)
☐	Improvement in your ability to do normal activities (social activities, work, school, family life)
☐	Improvement in sleep
☐	Changes in the overall quality of your life
☐	Changes in mood (for example, feeling less anxious or depressed)
☐	Reductions in your need for other pain medication
☐	Side effects

2. Are there other ways you might want a medication for pain to improve your health or your life (that is, other benefits you're seeking from treatment)?

○ Yes

○ No

If yes, what are these benefits?

Benefits March

1. You might like to know about the possibility of certain benefits and side effects because they affect your decision about which treatment to take.

Please rank the importance of the following as they relate to your decision to use or not use a treatment. Put the options in order from 1 to 7 such that:

1 MOST affects your decision
7 LEAST affects your decision

You can use the drop-down boxes to select your choices or drag the rows into your preferred order.

☐	Pain relief (reduced intensity or severity)
☐	Improvement in your ability to do normal activities (social activities, work, school, family life)
☐	Improvement in sleep
☐	Changes in the overall quality of your life
☐	Changes in mood (for example, feeling less anxious or depressed)
☐	Reductions in your need for other pain medication
☐	Side effects

2. Are there other ways you might want a medication for pain to improve your health or your life (that is, other benefits you're seeking from treatment)?

- ○ Yes
- ○ No

If yes, what are these benefits?

Benefits April

1. You might like to know about the possibility of certain benefits and side effects because they affect your decision about which treatment to take.

Please rank the importance of the following as they relate to your decision to use or not use a treatment. Put the options in order from 1 to 7 such that:

1 MOST affects your decision
7 LEAST affects your decision

You can use the drop-down boxes to select your choices or drag the rows into your preferred order.

[ ]	Pain relief (reduced intensity or severity)
[ ]	Improvement in your ability to do normal activities (social activities, work, school, family life)
[ ]	Improvement in sleep
[ ]	Changes in the overall quality of your life
[ ]	Changes in mood (for example, feeling less anxious or depressed)
[ ]	Reductions in your need for other pain medication
[ ]	Side effects

2. Are there other ways you might want a medication for pain to improve your health or your life (that is, other benefits you're seeking from treatment)?

○ Yes

○ No

If yes, what are these benefits?

Benefits May

1. You might like to know about the possibility of certain benefits and side effects because they affect your decision about which treatment to take.

Please rank the importance of the following as they relate to your decision to use or not use a treatment. Put the options in order from 1 to 7 such that:

1 MOST affects your decision
7 LEAST affects your decision

You can use the drop-down boxes to select your choices or drag the rows into your preferred order.

☐	Pain relief (reduced intensity or severity)
☐	Improvement in your ability to do normal activities (social activities, work, school, family life)
☐	Improvement in sleep
☐	Changes in the overall quality of your life
☐	Changes in mood (for example, feeling less anxious or depressed)
☐	Reductions in your need for other pain medication
☐	Side effects

2. Are there other ways you might want a medication for pain to improve your health or your life (that is, other benefits you're seeking from treatment)?

- ◯ Yes
- ◯ No

If yes, what are these benefits?

Benefits June

1. You might like to know about the possibility of certain benefits and side effects because they affect your decision about which treatment to take.

Please rank the importance of the following as they relate to your decision to use or not use a treatment. Put the options in order from 1 to 7 such that:

1 MOST affects your decision
7 LEAST affects your decision

You can use the drop-down boxes to select your choices or drag the rows into your preferred order.

☐	Pain relief (reduced intensity or severity)
☐	Improvement in your ability to do normal activities (social activities, work, school, family life)
☐	Improvement in sleep
☐	Changes in the overall quality of your life
☐	Changes in mood (for example, feeling less anxious or depressed)
☐	Reductions in your need for other pain medication
☐	Side effects

2. Are there other ways you might want a medication for pain to improve your health or your life (that is, other benefits you're seeking from treatment)?

○ Yes

○ No

If yes, what are these benefits?

Benefits July

1. You might like to know about the possibility of certain benefits and side effects because they affect your decision about which treatment to take.

Please rank the importance of the following as they relate to your decision to use or not use a treatment. Put the options in order from 1 to 7 such that:

1 MOST affects your decision
7 LEAST affects your decision

You can use the drop-down boxes to select your choices or drag the rows into your preferred order.

☐	Pain relief (reduced intensity or severity)
☐	Improvement in your ability to do normal activities (social activities, work, school, family life)
☐	Improvement in sleep
☐	Changes in the overall quality of your life
☐	Changes in mood (for example, feeling less anxious or depressed)
☐	Reductions in your need for other pain medication
☐	Side effects

2. Are there other ways you might want a medication for pain to improve your health or your life (that is, other benefits you're seeking from treatment)?

○ Yes

○ No

If yes, what are these benefits?

Benefits August

1. You might like to know about the possibility of certain benefits and side effects because they affect your decision about which treatment to take.

Please rank the importance of the following as they relate to your decision to use or not use a treatment. Put the options in order from 1 to 7 such that:

1 MOST affects your decision
7 LEAST affects your decision

You can use the drop-down boxes to select your choices or drag the rows into your preferred order.

☐	Pain relief (reduced intensity or severity)
☐	Improvement in your ability to do normal activities (social activities, work, school, family life)
☐	Improvement in sleep
☐	Changes in the overall quality of your life
☐	Changes in mood (for example, feeling less anxious or depressed)
☐	Reductions in your need for other pain medication
☐	Side effects

2. Are there other ways you might want a medication for pain to improve your health or your life (that is, other benefits you're seeking from treatment)?

◯ Yes

◯ No

If yes, what are these benefits?

Benefits September

1. You might like to know about the possibility of certain benefits and side effects because they affect your decision about which treatment to take.

Please rank the importance of the following as they relate to your decision to use or not use a treatment. Put the options in order from 1 to 7 such that:

1 MOST affects your decision
7 LEAST affects your decision

You can use the drop-down boxes to select your choices or drag the rows into your preferred order.

☐	Pain relief (reduced intensity or severity)
☐	Improvement in your ability to do normal activities (social activities, work, school, family life)
☐	Improvement in sleep
☐	Changes in the overall quality of your life
☐	Changes in mood (for example, feeling less anxious or depressed)
☐	Reductions in your need for other pain medication
☐	Side effects

2. Are there other ways you might want a medication for pain to improve your health or your life (that is, other benefits you're seeking from treatment)?

- ◯ Yes
- ◯ No

If yes, what are these benefits?

Benefits October

1. You might like to know about the possibility of certain benefits and side effects because they affect your decision about which treatment to take.

Please rank the importance of the following as they relate to your decision to use or not use a treatment. Put the options in order from 1 to 7 such that:

1 MOST affects your decision
7 LEAST affects your decision

You can use the drop-down boxes to select your choices or drag the rows into your preferred order.

☐	Pain relief (reduced intensity or severity)
☐	Improvement in your ability to do normal activities (social activities, work, school, family life)
☐	Improvement in sleep
☐	Changes in the overall quality of your life
☐	Changes in mood (for example, feeling less anxious or depressed)
☐	Reductions in your need for other pain medication
☐	Side effects

2. Are there other ways you might want a medication for pain to improve your health or your life (that is, other benefits you're seeking from treatment)?

○ Yes

○ No

If yes, what are these benefits?

Benefits November

1. You might like to know about the possibility of certain benefits and side effects because they affect your decision about which treatment to take.

Please rank the importance of the following as they relate to your decision to use or not use a treatment. Put the options in order from 1 to 7 such that:

1 MOST affects your decision
7 LEAST affects your decision

You can use the drop-down boxes to select your choices or drag the rows into your preferred order.

☐	Pain relief (reduced intensity or severity)
☐	Improvement in your ability to do normal activities (social activities, work, school, family life)
☐	Improvement in sleep
☐	Changes in the overall quality of your life
☐	Changes in mood (for example, feeling less anxious or depressed)
☐	Reductions in your need for other pain medication
☐	Side effects

2. Are there other ways you might want a medication for pain to improve your health or your life (that is, other benefits you're seeking from treatment)?

- ◯ Yes
- ◯ No

If yes, what are these benefits?

Benefits December

1. You might like to know about the possibility of certain benefits and side effects because they affect your decision about which treatment to take.

Please rank the importance of the following as they relate to your decision to use or not use a treatment. Put the options in order from 1 to 7 such that:

1 MOST affects your decision
7 LEAST affects your decision

You can use the drop-down boxes to select your choices or drag the rows into your preferred order.

☐	Pain relief (reduced intensity or severity)
☐	Improvement in your ability to do normal activities (social activities, work, school, family life)
☐	Improvement in sleep
☐	Changes in the overall quality of your life
☐	Changes in mood (for example, feeling less anxious or depressed)
☐	Reductions in your need for other pain medication
☐	Side effects

2. Are there other ways you might want a medication for pain to improve your health or your life (that is, other benefits you're seeking from treatment)?

- ○ Yes
- ○ No

If yes, what are these benefits?

Safety January

1. Specific side effects might affect your decision about which drug to take.

This survey asks you to rank a few of the many possible side effects that might be important to you. If the list below does not include side effects that you consider most important, you can enter them in the next question.

For these seven items, please rank the importance of the following side effects as they would relate to your decision to use or not use a drug.

Put the options in order from 1 to 7 such that:

1 MOST affects your decision
7 LEAST affects your decision

(Use the drop-down boxes to select your choices or drag the rows into your preferred order.)

☐	Nightmares
☐	Loss of sex drive
☐	Fainting, difficulty balancing, feeling unsteady
☐	Coughing
☐	Abnormal results from a blood test
☐	Decreased sense of touch
☐	Depression or low mood

2. Are there other potential side effects you want to know about before starting a medication?

- ◯ Yes
- ◯ No

If yes, what are the other possible side effects?

3. When you decide if you want to take a new medication, which is more important to you, (i) the likelihood that the medication will reduce your symptoms or (ii) the likelihood that you will experience side effects?

- ◯ The likelihood of feeling better is most important
- ◯ The likelihood of side effects is most important
- ◯ Feeling better and side effects are equally important

Other (please specify)

Safety February

1. Specific side effects might affect your decision about which drug to take.

This survey asks you to rank a few of the many possible side effects that might be important to you. If the list below does not include side effects that you consider most important, you can enter them in the next question.

For these seven items, please rank the importance of the following side effects as they would relate to your decision to use or not use a drug.

Put the options in order from 1 to 7 such that:

1 MOST affects your decision
7 LEAST affects your decision

(Use the drop-down boxes to select your choices or drag the rows into your preferred order.)

☐	Memory loss or difficulty thinking clearly
☐	Daytime sleepiness, feeling tired
☐	Involuntary muscle movements (e.g., twitching, trembling, rigid muscles, muscle spasms)
☐	Feeling unusually angry or aggressive
☐	Weight gain
☐	Hair or nail loss or discoloration
☐	Gastrointestinal problems (diarrhea, constipation, pain, bloating, indigestion, nausea, vomiting)

2. Are there other potential side effects you want to know about before starting a medication?

- Yes
- No

If yes, what are the other possible side effects?

3. When you decide if you want to take a new medication, which is more important to you, (i) the likelihood that the medication will reduce your symptoms or (ii) the likelihood that you will experience side effects?

- The likelihood of feeling better is most important
- The likelihood of side effects is most important
- Feeling better and side effects are equally important

Other (please specify)

Safety March

1. Specific side effects might affect your decision about which drug to take.

This survey asks you to rank a few of the many possible side effects that might be important to you. If the list below does not include side effects that you consider most important, you can enter them in the next question.

For these seven items, please rank the importance of the following side effects as they would relate to your decision to use or not use a drug.

Put the options in order from 1 to 7 such that:

1 MOST affects your decision
7 LEAST affects your decision

(Use the drop-down boxes to select your choices or drag the rows into your preferred order.)

[ ]	Death
[ ]	Insomnia (problems getting to sleep or staying asleep)
[ ]	Skin problems (e.g., dry skin, acne, rash)
[ ]	Headache
[ ]	Pain in the joints or muscles
[ ]	Itching, tingling, or burning sensation on the skin
[ ]	Swelling (e.g., in the hands, legs, or face)

2. Are there other potential side effects you want to know about before starting a medication?

- ◯ Yes
- ◯ No

If yes, what are the other possible side effects?

3. When you decide if you want to take a new medication, which is more important to you, (i) the likelihood that the medication will reduce your symptoms or (ii) the likelihood that you will experience side effects?

- ◯ The likelihood of feeling better is most important
- ◯ The likelihood of side effects is most important
- ◯ Feeling better and side effects are equally important

Other (please specify)

Safety April

1. Specific side effects might affect your decision about which drug to take.

This survey asks you to rank a few of the many possible side effects that might be important to you. If the list below does not include side effects that you consider most important, you can enter them in the next question.

For these seven items, please rank the importance of the following side effects as they would relate to your decision to use or not use a drug.

Put the options in order from 1 to 7 such that:

1 MOST affects your decision
7 LEAST affects your decision

(Use the drop-down boxes to select your choices or drag the rows into your preferred order.)

☐	Feeling unusually angry or aggressive
☐	Daytime sleepiness, feeling tired
☐	Coughing
☐	Depression or low mood
☐	Gastrointestinal problems (diarrhea, constipation, pain, bloating, indigestion, nausea, vomiting)
☐	Decreased sense of touch
☐	Hair or nail loss or discoloration

2. Are there other potential side effects you want to know about before starting a medication?

- ◯ Yes
- ◯ No

If yes, what are the other possible side effects?

3. When you decide if you want to take a new medication, which is more important to you, (i) the likelihood that the medication will reduce your symptoms or (ii) the likelihood that you will experience side effects?

- ◯ The likelihood of feeling better is most important
- ◯ The likelihood of side effects is most important
- ◯ Feeling better and side effects are equally important

Other (please specify)

Safety May

1. Specific side effects might affect your decision about which drug to take.

This survey asks you to rank a few of the many possible side effects that might be important to you. If the list below does not include side effects that you consider most important, you can enter them in the next question.

For these seven items, please rank the importance of the following side effects as they would relate to your decision to use or not use a drug.

Put the options in order from 1 to 7 such that:

1 MOST affects your decision
7 LEAST affects your decision

(Use the drop-down boxes to select your choices or drag the rows into your preferred order.)

☐	Decreased sense of touch
☐	Itching, tingling, or burning sensation on the skin
☐	Pain in the joints or muscles
☐	Death
☐	Depression or low mood
☐	Coughing
☐	Daytime sleepiness, feeling tired

2. Are there other potential side effects you want to know about before starting a medication?

- ◯ Yes
- ◯ No

If yes, what are the other possible side effects?

3. When you decide if you want to take a new medication, which is more important to you, (i) the likelihood that the medication will reduce your symptoms or (ii) the likelihood that you will experience side effects?

- ◯ The likelihood of feeling better is most important
- ◯ The likelihood of side effects is most important
- ◯ Feeling better and side effects are equally important

Other (please specify)

Safety June

1. Specific side effects might affect your decision about which drug to take.

This survey asks you to rank a few of the many possible side effects that might be important to you. If the list below does not include side effects that you consider most important, you can enter them in the next question.

For these seven items, please rank the importance of the following side effects as they would relate to your decision to use or not use a drug.

Put the options in order from 1 to 7 such that:

1 MOST affects your decision
7 LEAST affects your decision

(Use the drop-down boxes to select your choices or drag the rows into your preferred order.)

☐	Nightmares
☐	Memory loss or difficulty thinking clearly
☐	Fainting, difficulty balancing, feeling unsteady
☐	Weight gain
☐	Loss of sex drive
☐	Abnormal results from a blood test
☐	Involuntary muscle movements (e.g., twitching, trembling, rigid muscles, muscle spasms)

2. Are there other potential side effects you want to know about before starting a medication?

- Yes
- No

If yes, what are the other possible side effects?

3. When you decide if you want to take a new medication, which is more important to you, (i) the likelihood that the medication will reduce your symptoms or (ii) the likelihood that you will experience side effects?

- The likelihood of feeling better is most important
- The likelihood of side effects is most important
- Feeling better and side effects are equally important

Other (please specify)

Safety July

1. Specific side effects might affect your decision about which drug to take.

This survey asks you to rank a few of the many possible side effects that might be important to you. If the list below does not include side effects that you consider most important, you can enter them in the next question.

For these seven items, please rank the importance of the following side effects as they would relate to your decision to use or not use a drug.

Put the options in order from 1 to 7 such that:

1 MOST affects your decision
7 LEAST affects your decision

(Use the drop-down boxes to select your choices or drag the rows into your preferred order.)

☐	Insomnia (problems getting to sleep or staying asleep)
☐	Fainting, difficulty balancing, feeling unsteady
☐	Memory loss or difficulty thinking clearly
☐	Skin problems (e.g., dry skin, acne, rash)
☐	Swelling (e.g., in the hands, legs, or face)
☐	Headache
☐	Nightmares

2. Are there other potential side effects you want to know about before starting a medication?

- ◯ Yes
- ◯ No

If yes, what are the other possible side effects?

3. When you decide if you want to take a new medication, which is more important to you, (i) the likelihood that the medication will reduce your symptoms or (ii) the likelihood that you will experience side effects?

- ◯ The likelihood of feeling better is most important
- ◯ The likelihood of side effects is most important
- ◯ Feeling better and side effects are equally important

Other (please specify)

Safety August

1. Specific side effects might affect your decision about which drug to take.

This survey asks you to rank a few of the many possible side effects that might be important to you. If the list below does not include side effects that you consider most important, you can enter them in the next question.

For these seven items, please rank the importance of the following side effects as they would relate to your decision to use or not use a drug.

Put the options in order from 1 to 7 such that:

1 MOST affects your decision
7 LEAST affects your decision

(Use the drop-down boxes to select your choices or drag the rows into your preferred order.)

☐	Abnormal results from a blood test
☐	Death
☐	Depression or low mood
☐	Feeling unusually angry or aggressive
☐	Fainting, difficulty balancing, feeling unsteady
☐	Insomnia (problems getting to sleep or staying asleep)
☐	Memory loss or difficulty thinking clearly

2. Are there other potential side effects you want to know about before starting a medication?

- Yes
- No

If yes, what are the other possible side effects?

3. When you decide if you want to take a new medication, which is more important to you, (i) the likelihood that the medication will reduce your symptoms or (ii) the likelihood that you will experience side effects?

- The likelihood of feeling better is most important
- The likelihood of side effects is most important
- Feeling better and side effects are equally important

Other (please specify)

Safety September

1. Specific side effects might affect your decision about which drug to take.

This survey asks you to rank a few of the many possible side effects that might be important to you. If the list below does not include side effects that you consider most important, you can enter them in the next question.

For these seven items, please rank the importance of the following side effects as they would relate to your decision to use or not use a drug.

Put the options in order from 1 to 7 such that:

1 MOST affects your decision
7 LEAST affects your decision

(Use the drop-down boxes to select your choices or drag the rows into your preferred order.)

☐	Itching, tingling, or burning sensation on the skin
☐	Coughing
☐	Swelling (e.g., in the hands, legs, or face)
☐	Daytime sleepiness, feeling tired
☐	Loss of sex drive
☐	Weight gain
☐	Gastrointestinal problems (diarrhea, constipation, pain, bloating, indigestion, nausea, vomiting)

2. Are there other potential side effects you want to know about before starting a medication?

- ◯ Yes
- ◯ No

If yes, what are the other possible side effects?

3. When you decide if you want to take a new medication, which is more important to you, (i) the likelihood that the medication will reduce your symptoms or (ii) the likelihood that you will experience side effects?

- ◯ The likelihood of feeling better is most important
- ◯ The likelihood of side effects is most important
- ◯ Feeling better and side effects are equally important

Other (please specify)

Safety October

1. Specific side effects might affect your decision about which drug to take.

This survey asks you to rank a few of the many possible side effects that might be important to you. If the list below does not include side effects that you consider most important, you can enter them in the next question.

For these seven items, please rank the importance of the following side effects as they would relate to your decision to use or not use a drug.

Put the options in order from 1 to 7 such that:

1 MOST affects your decision
7 LEAST affects your decision

(Use the drop-down boxes to select your choices or drag the rows into your preferred order.)

☐	Headache
☐	Involuntary muscle movements (e.g., twitching, trembling, rigid muscles, muscle spasms)
☐	Nightmares
☐	Decreased sense of touch
☐	Hair or nail loss or discoloration
☐	Pain in the joints or muscles
☐	Skin problems (e.g., dry skin, acne, rash)

2. Are there other potential side effects you want to know about before starting a medication?

- ◯ Yes
- ◯ No

If yes, what are the other possible side effects?

3. When you decide if you want to take a new medication, which is more important to you, (i) the likelihood that the medication will reduce your symptoms or (ii) the likelihood that you will experience side effects?

- ◯ The likelihood of feeling better is most important
- ◯ The likelihood of side effects is most important
- ◯ Feeling better and side effects are equally important

Other (please specify)

Safety November

1. Specific side effects might affect your decision about which drug to take.

This survey asks you to rank a few of the many possible side effects that might be important to you. If the list below does not include side effects that you consider most important, you can enter them in the next question.

For these seven items, please rank the importance of the following side effects as they would relate to your decision to use or not use a drug.

Put the options in order from 1 to 7 such that:

1 MOST affects your decision
7 LEAST affects your decision

(Use the drop-down boxes to select your choices or drag the rows into your preferred order.)

☐	Headache
☐	Pain in the joints or muscles
☐	Weight gain
☐	Insomnia (problems getting to sleep or staying asleep)
☐	Gastrointestinal problems (diarrhea, constipation, pain, bloating, indigestion, nausea, vomiting)
☐	Skin problems (e.g., dry skin, acne, rash)
☐	Involuntary muscle movements (e.g., twitching, trembling, rigid muscles, muscle spasms)

2. Are there other potential side effects you want to know about before starting a medication?

- Yes
- No

If yes, what are the other possible side effects?

3. When you decide if you want to take a new medication, which is more important to you, (i) the likelihood that the medication will reduce your symptoms or (ii) the likelihood that you will experience side effects?

- The likelihood of feeling better is most important
- The likelihood of side effects is most important
- Feeling better and side effects are equally important

Other (please specify)

Safety December

1. Specific side effects might affect your decision about which drug to take.

This survey asks you to rank a few of the many possible side effects that might be important to you. If the list below does not include side effects that you consider most important, you can enter them in the next question.

For these seven items, please rank the importance of the following side effects as they would relate to your decision to use or not use a drug.

Put the options in order from 1 to 7 such that:

1 MOST affects your decision
7 LEAST affects your decision

(Use the drop-down boxes to select your choices or drag the rows into your preferred order.)

☐	Death
☐	Itching, tingling, or burning sensation on the skin
☐	Feeling unusually angry or aggressive
☐	Hair or nail loss or discoloration
☐	Swelling (e.g., in the hands, legs, or face)
☐	Loss of sex drive
☐	Abnormal results from a blood test

2. Are there other potential side effects you want to know about before starting a medication?

- ◯ Yes
- ◯ No

If yes, what are the other possible side effects?

3. When you decide if you want to take a new medication, which is more important to you, (i) the likelihood that the medication will reduce your symptoms or (ii) the likelihood that you will experience side effects?

- ◯ The likelihood of feeling better is most important
- ◯ The likelihood of side effects is most important
- ◯ Feeling better and side effects are equally important

Other (please specify)

Demographic Questions

1. In which year were you born?

2. What is your sex?

- Male
- Female
- Other (please specify)

3. Do you have pain related to any of these conditions?

- TMJ (temporomandibular joint and muscle disorders)
- Back pain
- Cancer
- Carpal tunnel syndrome
- Chronic pain after a surgery
- Chronic pelvic pain
- Complex regional pain syndrome
- Diabetes mellitus
- Fibromyalgia
- Guillain-Barré syndrome
- Lyme Disease
- Migraine
- Osteoarthritis
- Phantom limb pain
- Restless leg syndrome
- Shingles (postherpetic neuralgia)
- Stroke
- Trigeminal neuralgia
- None of the above

Other (please specify)

4. Using the image below, how would you rate your pain on a 0-10 scale at the present time, right now, where 0 is 'no pain' and 10 is 'worst possible pain'?

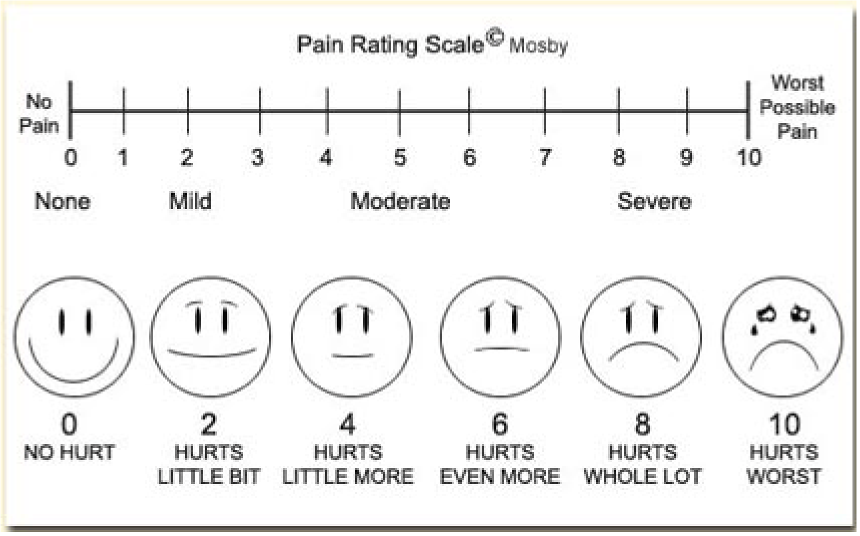
Pain Rating Scale© Mosby
No Pain
Worst Possible Pain
0 1 2 3 4 5 6 7 8 9 10
None
Mild
Moderate
Severe
0
NO HURT
2
HURTS LITTLE BIT
4
HURTS LITTLE MORE
6
HURTS EVEN MORE
8
HURTS WHOLE LOT
10
HURTS WORST

5. What treatments have you taken for pain?

	Taken in the PAST (more than 4 weeks ago)	CURRENTLY taking (within the last 4 weeks)
Ibuprofen (Motrin, Advil)	☐	☐
Aspirin (Bayer, Bufferin, Excedrin)	☐	☐
Naproxen (Aleve)	☐	☐
Acetaminophen (Paracetamol, Tylenol, Panadol)	☐	☐
Oxycodone (Oxycontin, Roxicodone, Oxecta)	☐	☐
Oxycodone with acetaminophen (Percocet)	☐	☐
Hydrocodone	☐	☐
Hydrocodone with acetaminophen (Vicodin, Lortab)	☐	☐
Tramadol (Ultram, ConZip, Ryzolt)	☐	☐
Cyclobenzaprine (Flexeril)	☐	☐
Carisoprodol (Soma)	☐	☐
Gabapentin (Neurontin)	☐	☐
Ketorolac (Toradol)	☐	☐
Diazepam (Valium)	☐	☐
Alprazolam (Xanax)	☐	☐
Clonazepam (Klonopin)	☐	☐
Acupuncture	☐	☐
Splints	☐	☐
Occlusal (Bite) Adjustment (orthodontics, bridges or crowns, teeth grinding)	☐	☐
Injections of corticosteroids	☐	☐
Injections of Botox	☐	☐
Surgery	☐	☐
TMJ Implants	☐	☐
Massage	☐	☐
None of the above	☐	☐

Other (please specify treatment and if taken in past or currently taking)

6. At what age were you diagnosed with a pain disorder?

Other (please specify)

7. Have you had any of the following side effects from a drug, device, or other treatment for your pain?

	Experienced in the PAST (more than 4 weeks ago)	CURRENTLY experiencing (within the last 4 weeks)
Fainting, difficulty balancing, feeling unsteady	☐	☐
Hair or nail loss or discoloration	☐	☐
Depression or low mood	☐	☐
Insomnia (problems getting to sleep or staying asleep)	☐	☐
Loss of sex drive	☐	☐
Itching, tingling, or burning sensation on the skin	☐	☐
Feeling nervous, anxious or on edge	☐	☐
Headache	☐	☐
Decreased sense of touch	☐	☐
Weight gain	☐	☐
Daytime sleepiness, feeling tired	☐	☐
Skin problems (e.g., dry skin, acne, rash)	☐	☐
Involuntary muscle movements (e.g., twitching, trembling, rigid muscles, muscle spasms)	☐	☐
Feeling unusually angry or aggressive	☐	☐
Memory loss or difficulty thinking clearly	☐	☐
Abnormal results from a blood test	☐	☐
Nightmares	☐	☐
Gastrointestinal problems (diarrhea, constipation, pain, bloating, indigestion, nausea, vomiting)	☐	☐
Swelling (e.g., in the hands, legs, or face)	☐	☐
Coughing	☐	☐
Pain in the joints or muscles	☐	☐
Never had a side effect from a drug, device, or other treatment for your pain	☐	☐

Other (please specify the side effect and if you experienced in the past or are currently experiencing)

8. If you have any comments about the questions, the format of the questions or suggestions for future surveys please write them here.

If you have questions about the survey contact the survey moderator at shutfle1@jhmi.edu

Thanks!
The MUDS team

S4: Patient co-investigator responses

Gabapentin outcomes		
**Definitely analyze, very important to patients**	**Possibly analyze, sometimes important to patients or important to some patients**	**Do not understand or know what this is**
• Anxiety (self-reported)<br>• Change in pain intensity (mean difference from baseline)<br>• Cognitive dysfunction<br>• Confusion<br>• Constipation<br>• Depression (self-reported)<br>• Discontinuation for any reason (number of participants who discontinued the drug)<br>• Discontinuation because of side effects (number of participants)<br>• Dizziness<br>• Emotional functioning/mood<br>• Fatigue<br>• Harm: benefit analysis<br>• Health-related quality of life<br>• Memory impairment<br>• Number of participants with self-reported improvement<br>• Pain affect (Short Form-McGill Pain Questionnaire)<br>• Participants experiencing one or more side effects (number of participants)<br>• Nausea<br>• Pain interference<br>• Physical activity<br>• Quality of life (daily pain score measured on an 11-point Likert scale)<br>• Serious adverse events (number of each event)<br>• Sexual dysfunction (patient write in)<br>• Somnolence<br>• Sleep	• Mean total SF-MPQ pain Scores<br>• Number of participants with clinician-reported improvement<br>• Number of responders (participants reporting clinically important reduction in daily pain intensity)<br>• Pain severity score	

Gabapentin outcomes		
**Definitely analyze, very important to patients**	**Possibly analyze, sometimes important to patients or important to some patients**	**Do not understand or know what this is**
• Sleep interferences (daily rating)<br>• Sleep interference scores/sleep difficulties<br>• Vertigo<br>• Vomiting		

Quetiapine outcomes		
**Definitely analyze, very important to patients**	**Possibly analyze, sometimes important to patients or important to some patients**	**Do not understand or know what this is**
• Change in anxiety (mean difference from baseline on a validated scale)<br>• Change in depression (mean difference from baseline)<br>• Cardiovascular effects (change in QTc interval duration, incidence of orthostatic hypotension)<br>• Change in weight<br>• Diabetes<br>• Discontinuation for any reason (number of participants who discontinued the drug)<br>• Discontinuation because of side effects (number of participants)<br>• Extrapyramidal symptoms (tardive dyskinesia, dystonia, akathisia)<br>• Functioning (mean score on the Global Assessment of Functioning scale)<br>• Health-related quality of life<br>• Hospitalization (number of participants hospitalized)<br>• Inability to concentrate<br>• Loss of energy<br>• Number of responders (participants reporting	• Number of remitters (participants scoring below the cut-off for a clinical episode)	• Change in fasting glucose level<br>• Change in triglycerides<br>• Hematologic effects (incidence of absolute neutrophil count (ANC) < 100/ml (count data/rate)<br>• Mean change in serum prolactin levels<br>• Mean score for measures of extrapyramidal symptoms (e.g. Abnormal Involuntary Movement Scale, Condensed User's Scale, Simpson-Angus Scale, Barnes Akathesia Rating Scale, or similar scale)

Quetiapine outcomes		
**Definitely analyze, very important to patients**	**Possibly analyze, sometimes important to patients or important to some patients**	**Do not understand or know what this is**
clinically important reduction in depression rating)<br>• Participants experiencing any extrapyramidal symptoms<br>• Participants experiencing one or more side effects (number of participants)<br>• Serious adverse events (number of each event)<br>• Specific side effects (number of each side effect organized using standard classifications)<br>• Sexual side-effects (patient write-in)<br>• Suicide<br>• Symptoms related to daytime drowsiness<br>• Weight gain		

S5: TMJ survey participant characteristics

S5a: Current and past treatments taken for pain (N=388)

	Current[1]		Past[2]	
	N	(%)	N	(%)
Ibuprofen (Motrin, Advil)	190	(48.97)	196	(50.52)
Acetaminophen (Paracetamol, Tylenol, Panadol)	118	(30.41)	160	(41.24)
Massage	104	(26.8)	193	(49.74)
Occlusal (Bite) Adjustment	101	(26.03)	159	(40.98)
Splints	100	(25.77)	148	(38.14)
Naproxen (Aleve)	90	(23.2)	153	(39.43)
Cyclobenzaprine (Flexeril)	67	(17.27)	132	(34.02)
Aspirin (Bayer, Bufferin, Excedrin)	60	(15.46)	120	(30.93)
Hydrocodone with acetaminophen (Vicodin, Lortab)	44	(11.34)	123	(31.7)
Gabapentin (Neurontin)	43	(11.08)	103	(26.55)
Tramadol (Ultram, ConZip, Ryzolt)	35	(9.02)	111	(28.61)
Diazepam (Valium)	32	(8.25)	76	(19.59)
Clonazepam (Klonopin)	31	(7.99)	52	(13.4)
Injections of corticosteroids	28	(7.22)	120	(30.93)
Oxycodone (Oxycontin, Roxicodone, Oxecta)	25	(6.44)	73	(18.81)
Acupuncture	25	(6.44)	123	(31.7)
Oxycodone with acetaminophen (Percocet)	25	(6.44)	102	(26.29)
Alprazolam (Xanax)	24	(6.19)	65	(16.75)
Hydrocodone	23	(5.93)	87	(22.42)
Carisoprodol (Soma)	14	(3.61)	44	(11.34)
Surgery	14	(3.61)	115	(29.64)
TMJ implant	14	(3.61)	56	(14.43)
Injections of Botox	13	(3.35)	58	(14.95)
Ketorolac (Toradol)	7	(1.8)	55	(14.18)
None	7	(1.8)	2	(0.52)

[1]Current: Treatments used within the last four weeks before completing the survey
[2]Past: Treatments used more than four weeks prior to the survey

S5b: Reported side effects from treatments taken for pain (N=388)

	Current[1] N	(%)	Past[2] N	(%)
Daytime sleepiness, feeling tired	120	(30.93)	132	(34.02)
Gastrointestinal problems (diarrhea, constipation, pain, bloating, indigestion, nausea, vomiting)	106	(27.32)	168	(43.3)
Headache	103	(26.55)	162	(41.75)
Pain in the joints or muscles	101	(26.03)	59	(15.21)
Insomnia (problems getting to sleep or staying asleep)	96	(24.74)	93	(23.97)
Memory loss or difficulty thinking clearly	90	(23.2)	131	(33.76)
Depression or low mood	77	(19.85)	118	(30.41)
Loss of sex drive	71	(18.3)	97	(25)
Involuntary muscle movements (e.g., twitching, trembling, rigid muscles, muscle spasms)	65	(16.75)	83	(21.39)
Feeling nervous, anxious or on edge	65	(16.75)	117	(30.15)
Weight gain	64	(16.49)	116	(29.9)
Fainting, difficulty balancing, feeling unsteady	60	(15.46)	109	(28.09)
Itching, tingling, or burning sensation on the skin	51	(13.14)	93	(23.97)
Skin problems (e.g., dry skin, acne, rash)	35	(9.02)	59	(15.21)
Swelling (e.g., in the hands, legs, or face)	34	(8.76)	133	(34.28)
Hair or nail loss or discoloration	30	(7.73)	41	(10.57)
Nightmares	28	(7.22)	70	(18.04)
Feeling unusually angry or aggressive	26	(6.7)	75	(19.33)
Never had a side effect from a drug, device, or other treatment for your pain	24	(6.19)	26	(6.7)
Decreased sense of touch	19	(4.9)	32	(8.25)
Coughing	14	(3.61)	32	(8.25)
Abnormal results from a blood test	13	(3.35)	43	(11.08)

[1]Current: Side effects experienced within the last four weeks before completing the survey

[2]Past: Side effects experienced more than four weeks prior to the survey

S6: All "other" potential benefits and harms reported by survey participants in open-ended questions

Note: In S6a and S6b, we corrected obvious spelling and capitalization errors. We also capitalized the first word of each line. We did not edit responses for word choice or grammar.

S6a: Responses to the question "Are there other ways you might want a medication for pain to improve your health or your life (that is, other benefits you're seeking from treatment)?"

A medication that eliminates or even reduces chronic pain without horrible side effects -- and that a doctor isn't afraid to prescribe!
A pain medication that didn't require me to take another medication for constipation. It is difficult to have a decent quality of life if you are tied to a bathroom and cant leave the house!
A reduction in pain that allows me to continue to do the normal activities of my day.
Ability to concentrate while taking; less ups and downs in terms of the medication wearing off.
Ability to eat. Improve sex life.
Ability to enjoy a meal without jaw muscle spasms or pain
Ability to take medication during the day that doesn't affect sleepiness, lethargy, or my ability to do my job, so that I may take something that will help when my TMJ has an extreme jump in pain levels.
Able to socialize better because not in so much pain.
Added energy. I noticed while on my pain medication regimen, when I started taking Adipex to lose weight it did not interfere with my pain meds and gave me some energy even though I was still hurting a good bit (normal for my pain regimen)
Antidepressant. Anxiety
Anything that doesn't make it worse
As many options as possible in case a med doesn't work
Be more effective without danger to body work for a longer period of time so I don't have to remember to take it three times a day take less medications
Be part of a long term treatment plan to NOT be dependent or need medication permanently or long-term – to be part of a treatment program with proven efficacy combining other modalities such as physical therapy, perhaps mindfulness training and other self-help pain management techniques etc. Pain medication that is used ONLY on a short-term, as needed basis, rather than daily/permanently.
Benefits to my general health state and related medical conditions
Better function
Better pain control - It's horrible having stabbing pains in your face and neck and constant muscle spasms
By not providing a financial burden! Cost is almost highest priority for me in deciding whether or not I can take a med
Chronic migraines fibro IBS chronic muscle tension and more, id just like to have a life migraines are being treated but I think some of the underline cause is TMJ. Some is genetic.
Cost, ease of getting, reduced social stigma
Decrease the impulse or instinct to constantly clench my jaw
Enable the ability to exercise and spend higher quality of time with family
Energy to do what is needed to get things done
Financial responsibility for treatment
For the medicine to cure or fix or minimize the condition itself
Freedom to eat what I want
Help relax the muscles so face is less twisted
I have already had surgery, but am in pain every day. All prescription medication causes constipation, which is a MAJOR problem for me. I live on medication to fight the constipation, and had to have many surgeries because of this.
I have severe muscle spasms now and need relief, tried many but after a while they don't help. After many years of TMJ, I am now also diagnosed with Oral Mandibular Dystonia and was told it could be related to years of dentures or Medications used.

I like one that would stop the nerve tingling and burning sensation and also for it to control swelling
I sleep fine... But when I wake up I am sore or my jaw and neck feel tired
I want a pain medication that does not cause stomach/intestinal discomfort and can be used frequently without risk. NSAIDS all carry the risk of ulcers or bleeding.
I want the quality of life to improve. This could mean making me feel happier, relieving stress, reducing inflammation, and making it easier for me to have an active life.
I want to be able to chew my food. I am having to blend EVERYTHING because it is hard to chew. It is also hard to talk on the phone, talk generally and swallow.
I would like a medication that I would at least be able to work a job to afford to live a little life outside of work.
I would like medication in the more general area o TMJ as it relates to and now causes severe burning facial pain as well as jaw pain. IT IS HARD TO SEPARATE IF THE PAIN IS FROM MUSCLE, JOINT DEGENERATION, i.e. bone one bone, and wearing away of joint, AND NERVE PAIN. My pain is so much more than I can explain. Trying a sphenopalatine ganglion block soon, and have Botox for migraines now. Also, physical therapists are not really trained in this area and can cause more hard than good! I have a tried every non- narcotic pain reliever AND anti-depressants, etc. AND narcotics. Only on meloxicam and 3500 mg of Tylenol a day and it is not enough
I would like to not feel depressed due to pain every day.
I'd like to be able to relieve pain without fearing that taking medication over lengthy periods will damage other aspects of my health.
I'm sure there are other ways, but at the moment I can't think of anything other than what was said in question 1
Ideally I don't want to end the pain, but solve the problem causing the pain
If medication can relieve pain, it may make it easier to eat, to talk, to laugh. All of these things will improve daily quality of life which can improve mood and satisfaction with life circumstances.
Improved jaw movement, Improved jaw usage
Improvement in hearing loss.
Improvement in sleep.
Improving overall health and say to day ability
Increased range of motion of the jaw
It can relieve pain in other areas aside from TMJ and soddi patients are able to take them.
It would be nice if there was a cure instead of treating whatever the problem is.
Joint pain
Less dependence on the drug; less interactions with other drugs.
Less fatigue
Less pain in my jaw would allow me to eat more types of foods. I haven't been able to eat steak in 7years for example.
Less swelling
Long term affects
Make it easier to talk or make facial expressions, to help work and social life.
Medication isn't a cure it's a Band-Aid
Medication that will help more than one health challenge at a time. Example: TMJ, Fibromyalgia & muscular / skeletal challenges. Help for mental confusion due to drugs (Clearness of mind).
More normal function of my TMJ. Less difficulty eating, less spasm of joint, improved sensation to mouth area (post op nerve damage).
More positive outlook for the future would be direct benefit of successful pain management.
More sleep since I can not take a RX sleep med because of side effects. Also I would want more pain relief
My ability to eat normally again
Nerve pain
NEW PAIN MEDS THAT ACTUALLY WORK AROUND THE CLOCK FOR THOSE OF US WHO HAVE SEVERE PAIN...I BANDAID CANNOT FIX AN UNDERDEVELOPED JAW. BESIDES SLEEPING SEDATIVES THERE ARE NO MEDS FOR SEVERE TMD PEOPLE THAT DECREASE PAIN SO ONE HAVE SOME QUALITY OF LIFE. THE DOCTORS NEED TO GIVE PAIN MEDS & MEDS THAT INSURANCE COVERS SO PARENTS ARE NOT OUT $60,000 AND THINGS GET WORSE AS NO ONE IN SMALLER

AREAS WORK WITH JAW ISSUES AS IT IS NOT COVERED BY MEDICAL OR DENTAL UNLESS CAUSED BY AN AUTO ACCIDENT. WILLING TO TRY ANYTHING.
Not add additional side affects
Not affect my mental focus and give me my energy back
Not feeling medicated and dependent upon taking pain medication to life my life
Not make me gain weight
Not make me nauseous or cause damage to my GI tract
Not sure
Not to become dependent
Overall feeling better
Pain and inflammation are the worst.
Pain free with no side effects
Pain management clinic
Pain reduction without affecting my level of energy/concentration.
Pain relief without harming my weak heart
Preferably the less harmful medications, the better. I don't want it messing up my body in exchange for less jaw pain.
Quality of life
Reduce grinding and discomfort from misalignment
Reduce muscle tightness and inflammation, on the one side of my face that hurts
Reduce pain induced stress
Reduced frequency; 1-2x/week vs. 4-5x/week
Reduced need for more invasive procedures (e.g. surgery, injections)
Reduction of pain with less side effects. Less fatigue and gastro intestinal problems. The many other treatments available non invasive but not covered under most insurance plans. Too many patients are very restricted in coverage to get any relief with too high a copay eligibility to receive any benefit at all.
Relief from the extreme muscle tightness in my mouth, neck and shoulder area.
Restore energy and ambition I have lost through years of dealing with chronic pain with its resulting lowering the overall quality of my life
Similar to ability to do "normal activities"...having improved function of the body part which pain impairs.
Smaller doses that would last longer
Take my migraines away
Take the TMJ pain away so I can chew food instead of always looking for soft food. Also take the pain away so I don't eat for comfort and gain weight. Take the pain away so I feel like talking to people, since it hurts to talk.
That I have control, depending on level of pain of how much medication I need. That the script is written in such a way I can take what I need----less or more (I understand there has to be some limitations) but it is always individual. My decision of how much medication I take also depends on what I'm doing at the time i.e. just resting at home or if I have to drive.
The long acting OxyContin and Dilaudid and another time gabapentin, put immediate rapid weight gain larger than 10 pounds per month. The Dr. did not seem to care that the medication he gave me was a rapid fat factory of 70+ pounds in 7 months! 7 months and I have to carry around the equivalent to 70 lb. bags of dog food! (could not eat a lot due to closed jawjoint etc. So it was definitely the medication that caused this.) which caused other severe health problems due to this. When I was put on normal hydrocodone, the weight gain stopped immediately. The Doctors just have to listen to their patients! My husband is a physician and the doctors refused to listen to him! Those doctors not listening to the problems the medication cause was unethical!
The medications that currently work for me now also help reduce my migraines.
TMJ relief
To be able to feel & think like a normal person again. To not wake up all hours of the night with pain.
To be able to fly in a airplane. Do be able to do activities.
To be able to have a longer acting medication without that foggy feeling or feeling as though you could just fall asleep

on the spot
To be able to open my mouth wider
To be able to wake up in the morning and not have chronic jaw pain and migraines. I also want to have my life back somewhat as possible without pain.
To be out of pain is my one goal
To eventually stop meds - in other words, a medication that will cure!
To free up brain capacity that was being used by being in pain such that my attention span might be improved and I could be more 'present' as opposed to common side effects of pain medication that leave you feeling sort of zombie-like
To have one day of no pain when I wake up!
To not feel drugged or confused.
To relieve the systemic issues (i.e., GI, Dry Eyes, Fevers...
To treat only flare ups when they become severe, but don't have to take medication constantly.
Use of antipsychotic tranquilizers for pain relief from reflex sympathetic dystrophy order. Causing clenching of the teeth uncontrolled pain
Want to be alert and not impaired on pain meds.
With decreased pain, improvement in muscle skeletal pain and spasm decrease on collateral areas related to the TMJ.

S6b: Responses to the question "Are there other potential side effects you want to know about before starting a medication?"

1.might the medication cause cancer. 2. Might the medication cause synergism? I am taking medication for Hypertension, and Diabetes.
1. Is the medication addictive? 2. More serious side effects e.g. liver damage etc.
1) Digestive issues such as stomach pain, diarrhea, constipation, etc. I often have. 2) Nightmares, hallucinations (I had with codeine)
Absolutely. The worst problem is nausea from pain medications
Addiction
Addiction
addictive qualities
affect on other conditions
Affects on thyroid function. Weakness. Muscle fatigue.
all of them!
All of them!
All side effects
All side effects!
All.
Any
Any adverse affects on my well being.
Any alteration in mental mood, clarity, cloudy thinking (the last question made it hard to indicate how important this is because the other listed side effects were so severe and frankly unusual sounding)
any and all
any and all side effects
Any and all side effects to include "rare" side effects. I am allergic to many medications and many rare side effects pertain to my situation.
ANY and ALL....I found it difficult to rate them in the question above because all of the side effects would be very unpleasant
Any effect on mood, increase in anxiety or depression.
Any interactions with other medications
Any interference with my other medications
Any long term side affects that would affect my future health
Any of them.
Any of them.
Any other side effects
Any possible side effects
Any, and all, that exist
Any!
Anything and everything that might affect your health.
Anything else that might affect my quality of like or could be life-threatening.
Anything! Stupid question...vertigo...rapid heart beat, etc.
Appetite stimulant, grogginess or mental acuity
Blurred vision, altered perception, how it effects the heart and other organs, interactions with other drugs and herbs
Bone loss
Breathing , mood, and alertness
Can I absorb it after my gastric bypass

Can you become addicted to the medication
Cause cancer
Causes cancer
Causes other medical problems
Cognitive changes, perception, and anything that might be undesirably permanent.
Cognitive issues
Cognitive-memory
Constipation and if/how it interacts with other medications I'm on.
Constipation or diarrhea
Constipation/diarrhoea; headaches or migraines; interaction with other medication.
Contraindications with my other medications.
Could the medication be addictive? Might the medication cause anxiety, tension, increased blood pressure?
Damage to heart, lungs, kidneys or bone loss, etc.
Damage to my internal organs with long term use
Damage to organs
Damage to organs (e.g., liver, kidney)
Danger to body organs such as kidneys of heart
Dependency on the new medication. Is it worse than the old medications.
Depression, anxiety, sleepiness, interactions
Difficulty breathing, severe allergic reaction, cancer potential
Difficulty in the respiratory, or cardiac systems. Also contraindications with other medications. Also additive effects with other medications
Digestive system side effects
Dizziness and/ or nausea, risk of stomach bleeding or ulcers, risk of liver or kidney damage, racing heart
Dizziness, getting other diseases, death
Does it damage internal organs. Does it affect other prescription drugs that I take.
Does the drug cause damage to the liver or kidneys. Can the drug cause seizures.
Don't know until I see them
Drowsiness
Drowsiness
Drowsiness, Depression
Drowsiness, seizures, depression.
Drug interactions
Effect on blood pressure and pancreas
Effect on diet (increased/decreased) weight, digestion (gastric reflux, constipation, respiratory system, drowsiness, behavioral changes, addiction possibilities, interaction with other drugs, light headedness/fainting
Effect on internal organs e.g. the liver
Effect on organ functions. Costs. Addictions. Effectiveness.
Effects a medication may have on the liver or kidneys with long term use.
Effects of digestive system, gastrointestinal effects; constipation, diarrhea, circulatory system; blood pressure, clotting, endocrine system; effects toward hormones. Whether pain medication will effect your body creating situations that leads to disease.
Effects on heart, breathing, etc.
Everything about how this solves the problem vs just masking the pain. I'm not in favor of medication unless it heals.
Exacerbating my cardiomyopathy
Excessive weight gain

Feeling of being dizzy or sleepy. Upset stomach and vomiting.
G.I. Effects
Gastro intestinal
Gastrointestinal problems, edema, potential life threats, vision disturbances
Gastrointestinal side effects
Gastrointestinal, interaction with other medications
GI Bleed, cancer, paralysis, stroke, kidney failure.
GI symptoms
Grinding or clenching teeth and muscle tightness
Habit forming. Affect thinking
Habit-forming? Kidney/liver toxicity?
Hallucination, staying awake
Hard to consider taking drugs with many or any adverse side affects. Would likely skip.
Headaches and nausea and weight gain
Headaches, memory loss, sensitivity to sunlight, itching, water retention, seizures, difficulty breathing
Heart palpitations
Higher chance of heart attack or stroke?   Bad withdrawal. Affect asthma?
How affects blood pressure, weight gain. I like to know all side effects and how likely it is to see these side effects .
How it affects my mind. Foggy brain.
How it effects eye sight. Although not a side effect, is it approved for this medical problem or is in trial or helped with some but not approved.
How it effects my day to day functionality (does it turn me into a zombie?) - and interactions with other medications.
How it effects the digestive system.
how it reacts with other drugs
How would it affect my mood/depression/anxiety.
I do my best to do without pain pills. All side effects are too great a risk if they impair my ability to preform my job
I have difficulty focusing on things. I don’t know if that is the medication or fibromyalgia causing that. I also have ringing in my ears and chronic sinus infections. Also, I have no energy. I don't know what is causing these symptoms.
I have IBS and numerous other pain issues. Is this medicine compatible with what I currently use?
I like to know about all possibilities just in case
I like to know all side effects. I hate meds that make me feel like a zombie.
I want a baby and I can't get pregnant with that meds (Lyrica and Dilaudid)
I want to know about all side effects.
I want to know if the medication has any potential internal side effects (kidney/liver function, GERD, ulcers, etc.), I also would want to know if there is potential for joint deterioration over time of use, especially if it's an injectable medication directly to the joint. I want to know if my joint, which is where my pain is, is going to hold up to the medication, or if in the long run, the joint is going to weaken because of the medication. For that matter, I'd want to know if any of my other bones or joints would weaken because of a medication. I would also want to know if it would change things like my sugar levels, iron levels etc. over a long term period. I use these medications long term, so I am always looking at long term side effects, and rarely care about the short term side effects. (Except maybe death, as my ranking shows...)
I want to know of all potential side effects (so just would read patient leaflet included with drug)
I want to know them all...
I would like a list of all possible side effects.
I would like to know about any possible side effects - providers have skipped over that in the past.
I would want to know all potential side effects

I would want to know if a medication would affect my ability to sleep, as TMJ make sleep difficult. I would not want a medication that made me feel foggy or unable to do normal activities. I would not want a medication that upset my stomach or increased my blood sugar.
I'd want to any and all side effects that would affect my body negatively in ANY way no matter how long after I've taken the medication. It needs to be TRANSPARENT of what I'm allowing in order to have less pain.
I'm allergic to over 50 medications, so it's hard list for me. I'd have to literally compare all medications to be able to say.
If it can be taken during pregnancy.
If it can effect sleep
If it effects blood pressure.
If it is addictive.
If it might kill me would probably be #1
If there are any concerns relative to long term use such as damage to other organ sites or heart failure.
Increased chance of blood clots
Increased depression, affects on heart and brain
Increased heart rate
Increased tolerance and long term effects
Insomnia, tremors or racing heart
Insomnia
Interaction with other drugs
Interactions between all of my medications.
Interactions with medications I take on a daily basis.
Interactions with other medications
Interactions with other medications   Can I drive?
Interactions with other meds
Interactions with other meds and clinical trails outcomes relating to side effects and long term negative effects of the drugs.
Interactions with other RX meds I am taking for other illnesses/conditions.
Interactions with other medications
Intestinal problems
Is it addictive
Is the medicine addicting? Does it affect blood pressure?
Is there a possibility of becoming dependent
Issues with long term use.
Itching/rash or anxiety.
Joint damage
Lethargy, respiratory
Libido, nighttime sleepiness, weight gain, skin discoloration, interaction with other medications
Liver and kidney function changes; noticeable weight gain from medication.
Liver damage, stomach problems, loss of appetite
Liver kidney toxicity, chances of heart attack stroke
Liver or kidney toxicity
Liver, kidneys,
Long term affects rashes ability to function in daily routine does it make you too tired
Long term affects especially to liver, kidney and heart function.
Long term effects
Long term effects

Long term effects
Long term effects, carcinogenic in particular, bone loss
long term health effects
Long term relief
Long term side affects or how the medication impacts my internal organs.
Long term side effects, other effects to the body like IBS.
Long term use risks
Long term effects
Loss of ability to focus.
Loss of appetite, depressed mood, fatigue, affecting sleep (either sleeping too much or insomnia)
Loud, hard sneezing.
Lowered immunity, stomach upset, stroke and seizure increase,
Migraines
Memory loss and lack of focus
Mental fog, cognitive effects, confusion Constipation Poor judgment
Mental ones - Can it cause depression and/or suicidal thoughts? Will it increase my anxiety and give me panic attacks? Can it cause a dissociative mental state?
Migraines, seizures, cancer, bone loss
Muscle weakness loss of co ordination balance dizziness. The other serious side effects like passing out. Any severe brain issues like loss of memory or confusion.
Nausea
Nausea
Nausea, constipation, weight gain, loss of appetite, impact on libido
Nausea, heartburn, cancer
Nausea, potential effect on other bodily functions such as digestion, bad breath
Nausea, safe for breastfeeding/pregnancy
Nausea/vomiting
Nausea
Neuromuscular
Not being able to function to do normal things-- groggy
Pain meds have messed up my bowels for the rest of my life. They have caused other problems with my stomach like an ulcer and chronic constipation. Also, with TMJ there are times eating is a major problem which can lead to different vitamin deficiencies and I have found some medications have made this worse or it has caused other problems because one of the few foods I could eat/drink I was not supposed to. It also has been a problem with low energy or being too tired to go outside or do much of anything. That also makes vitamin deficiencies. Many pain meds aren't made for people that can not eat "normal" diet. Constipation is made worse because we can't chew many of the good veggies we need, pain meds also make it worse.
Permanent damage to my body
Possible cardiovascular, genitourinary, hepatic/renal or respiratory side-effects.
Potential for bone fracture or osteoporosis. The hobbies that make my life worth living include rock climbing, extreme cycling endurance events, and skiing, so the chance of a season ending fracture is high up on my list.
Potential long term damage
Raising cholesterol and triglyceride, sex drive, sleepiness
Rapid Weight Gain!!!! That you can eat 3 yogurts per day and still gain a pound!
Reproductive consequences
Respiratory issues i.e., Increase asthmatic problems. Sunlight sensitivity resulting rashes, eye sensitivity Migraine triggers

Risk of dependence
Risk of intestinal bleeding. I was prescribed Cymbalta when they wouldn't give me anything else, which led to more Advil & Aleve which caused a huge upper GI bleed in which I almost died (hemoglobin was down to 6!!)
Seizure possibilities
Seizures
Seizures, tiredness, mood swings.
Sexual dysfunction
Sexual side effects Addictive potential
Sleepiness
Sleepiness, lack of cognitive awareness
Stomach issues
Stomach upset, constipation, diarrhea, memory issues, concentration loss
Stomach/intestinal bleeding risk, ulcer risk. Interactions with other drugs I am taking. Interactions with alcohol.
Stomachaches
Sweating
Swelling, headaches
Tendon issues; joint issues - not just TMJ; carcinogenic issues
Unwanted gastrointestinal effects
Upset stomach, nausea, confusion, unable to concentrate
Vertigo, blurry vision, upset stomach
Vertigo, skin peeling, hyper mania
Vision and bladder control
Weight gain
Weight gain
Weight gain
Weight gain
Weight gain
Weight gain or loss
Weight gain or loss.
Weight gain or weight loss, vision problems, stomach issues
Weight gain or weight loss??
Weight gain, addiction
Weight gain, drowsiness.
Weight gain, nausea, difficulty sleeping, exhaustion
Weight gain, need for additional medications to counter side effects of new drug, loss of memory or cognitive function
Weight gain.
Weight gain.
Weight gain...
Weight gain/loss
Weight gain/loss
Weight loss or gain, heart rate changes,
What organs or interactions with prescriptions it might have on these issues
What all side effects are and what scale will they affect me
What are long term impacts such as stomach issues
What are the most common side effects, especially in women. How medications combined with thyroid replacement medication. I have hypothyroidism, extremely common condition. In many instances I have seen warnings along the

lines of use caution when combining with thyroid replacement, but generally practitioners/pharmacists were not aware of any specific details.
Whether it might affect my thyroid negatively, or make my thyroid medication less effective.
Will it give me dry mouth.
Would want to know all common side effects.
Yes, I'm the type of person who reads any and all side effects on the drug listing from the pharmacy and also do research on line

S7: Baseline characteristics of survey participants included in the final analysis by the reported month of birth (N=385)[1]

S7a: January to June

	January (N=23)		February (N=25)		March (N=25)		April (N=36)		May (N=27)		June (N=31)	
**Median years of age (IQR)**	42	(36,58)	47	(36,54)	55	(45,61)	53	(47,59)	52	(46,62)	50	(38,57)
**Number of women (%)**	22	(95.7)	24	(96.0)	23	(92)	34	(94.4)	25	(92.6)	28	(90.0)
**Median age diagnosed with a pain disorder (IQR)[2]**	26	(19, 37)	29	(20,36)	32	(22,36)	35	(24,45)	29	(19,34)	27	(18,36)
**Median present pain intensity (IQR)**	4	(2, 5)	5	(2, 7)	5	(2, 8)	4	(3, 7)	6	(3, 7)	5	(3, 6)
**Median number of comorbid pain conditions (IQR)**	2	(2, 4)	3	(2, 4)	2	(1, 3)	3	(1, 4)	3	(2, 4)	3	(2, 4)
**Median number of current pain medications (IQR)**	3	(1, 5)	2	(1, 5)	3	(2, 4)	3	(1, 4)	3	(2, 4)	4	(2, 4)
**Median number of past pain medications (IQR)**	8	(3, 12)	6	(3, 10)	6	(3, 13)	8	(4, 12)	6	(3, 12)	6	(3, 11)

S7b: July to December

	July (N=35)		August (N=33)		September (N=40)		October (N=39)		November (N=30)		December (N=41)	
**Median years of age (IQR)**	53	(46,60)	53	(47,62)	50	(39,59)	57	(46,64)	48	(40,57)	54	(44,62)
**Number of women (%)**	32	(91.4)	27	(81.8)	37	(92.5)	36	(92.3)	28	(93.3)	38	(92.7)
**Median age diagnosed with a pain disorder (IQR)[2]**	34	(24,45)	32	(20,42)	26	(21,35)	31	(21,37)	30	(23,40)	30	(22,35)
**Median present pain intensity (IQR)**	4	(3, 6)	5	(3, 7)	4	(2, 7)	5	(3, 7)	4	(2, 5)	4	(3, 7)
**Median number of comorbid pain conditions (IQR)**	2	(1, 3)	3	(2, 5)	2	(1, 5)	3	(2, 4)	2	(1, 3)	2	(2, 4)
**Median number of current pain medications (IQR)**	3	(1, 4)	3	(2, 4)	3	(1, 4)	4	(2, 5)	3	(1, 4)	2	(1, 5)
**Median number of past pain medications (IQR)**	5	(2, 7)	7	(3, 9)	5	(3, 9)	4	(2, 11)	6.5	(3, 10)	5	(3, 8)

Legend: IQR = inter-quartile range

[1] Characteristics are included for 385 participants. Three participants indicated that they preferred not to give their months of birth.

[2] 26 participants did not indicate the age they were diagnosed with a pain disorder: four were born in Feb, three in March, five in April, one in May, one in June, two in July, four in September, one in October, one in November and four in December
