# Supplementary material for: Methods to identify and prioritize patient-centered outcomes for use in comparative effectiveness research
Source: Pilot Feasibility Stud. 2018 Jun 12;4:95. doi: 10.1186/s40814-018-0284-6 (PMC6047482; doi:10.1186/s40814-018-0284-6)
Supplement: Supplementary file 2 — Patient and clinician co-investigator survey. (PDF 597 kb) [file 40814_2018_284_MOESM2_ESM.pdf]

Additional file 2: Patient and clinician co-investigator survey

MUDS Team,
In anticipation of our investigators meeting, we would like to get a better idea of what the team thinks are the most important outcomes for gabapentin and quetiapine.

As a starting point, we have created a list of outcomes that we think may be important to patients related to these medications.

Please let us know how important you think these outcomes are and add in new patient-centered outcomes at the bottom.

Thanks,
Evan and the MUDS team

1. Please rate the importance of analyzing each outcome in the gabapentin analyses, when data is available, during the MUDS study.

	Definitely analyze, very important to patients	Possibly analyze, sometimes important to patients or important to some patients	Definitely do NOT analyze, not important to patients	Do not understand or know what this is
Number of responders (participants reporting clinically important reduction in daily pain intensity)	○	○	○	○
Change in pain intensity (mean difference from baseline)	○	○	○	○
Serious adverse events (number of each event)	○	○	○	○
Number of participants with self-reported improvement	○	○	○	○
Number of participants with clinician-reported improvement	○	○	○	○
Depression (self-reported)	○	○	○	○
Anxiety (self-reported)	○	○	○	○
Pain affect (Short Form-McGill Pain Questionnaire)	○	○	○	○
Sleep interferences (daily rating)	○	○	○	○
Health-related quality of life	○	○	○	○
Discontinuation for any reason (number of participants who discontinued the drug)	○	○	○	○
Participants experiencing one or more side effects (number of participants)	○	○	○	○
Discontinuation because of side effects (number of participants)	○	○	○	○
Harm:benefit analysis	○	○	○	○
Cognitive dysfunction	○	○	○	○
Memory impairment	○	○	○	○
Fatigue	○	○	○	○
Vertigo	○	○	○	○
Dizziness	○	○	○	○
Nausea	○	○	○	○
Constipation	○	○	○	○
Vomiting	○	○	○	○
	○	○	○	○

Supporting Information: Identifying patient-centered outcomes

Pain interference				
Physical activity				
Sleep				
Emotional functioning/mood				
Sleep interference scores/sleep difficulties				
Quality of life daily pain score measured on an 11-point Likert scale				
Pain severity score				
Mean total SF-MPQ pain Scores				
Dizziness				
Somnolence				
Confusion				

List important outcomes that are important to patients missing from the above list. Only list the items that you feel should DEFINITELY be analyzed.

2. Please rate the importance of analyzing each outcome in the quetiapine analyses, when data is available, during the MUDS study.

	Definitely analyze, very important to patients	Possibly analyze, sometimes important to patients or important to some patients	Definitely do NOT analyze, not important to patients	Do not understand or know what this is
Number of responders (participants reporting clinically important reduction in depression rating)				
Change in depression (mean difference from baseline)				
Discontinuation for any reason (number of participants who discontinued the drug)				
Serious adverse events (number of each event)				
Number of remitters (participants scoring below the cut-off for a clinical episode)				
Functioning (mean score on the Global Assessment of Functioning scale)				
Health-related quality of life				
Change in anxiety (mean difference from baseline on a validated scale)				
Hospitalization (number of participants hospitalized)				
Suicide				
Participants experiencing one or more side effects (number of participants)				
Discontinuation because of side effects (number of participants)				
Specific side effects (number of each side effect organized using standard classifications)				
Change in fasting glucose level				
Change in triglycerides				
Diabetes				
Change in weight				
Cardiovascular effects (change in QTc interval duration, incidence of orthostatic hypotension)				
Extrapyramidal symptoms (tardive dyskinesia, dystonia, akathisia)				
Participants experiencing any extrapyramidal symptoms				
Mean score for measures of extrapyramidal symptoms (e.g. Abnormal Involuntary Movement Scale, Condensed User's Scale, Simpson-Angus Scale, Barnes Akathesia Rating Scale, or similar scale)				
Mean change in serum prolactin levels				
Hematologic effects (incidence of absolute neutrophil count (ANC) < 100/ml (count data/rate)				
Weight gain				
Symptoms related to daytime drowsiness				
Loss of energy				
Inability to concentrate				

List important outcomes that are important to patients missing from the above list. Only list the items that you feel should DEFINITELY be analyzed.

3. If you are aware of any publications that have identified outcomes that are important to patients with pain, bipolar disorder, gabapentin or quetiapine, please paste the citations below.

***4. Please enter your initials below so we know who has completed the survey.**

If you have questions about the survey contact Susie at shutfle1@jhmi.edu

Thanks!
Evan and the MUDS team
