# Supplementary material for: Methods to identify and prioritize patient-centered outcomes for use in comparative effectiveness research
Source: Pilot Feasibility Stud. 2018 Jun 12;4:95. doi: 10.1186/s40814-018-0284-6 (PMC6047482; doi:10.1186/s40814-018-0284-6)
Supplement: Supplementary file 3 — Part 2 Survey. (PDF 905 kb) [file 40814_2018_284_MOESM3_ESM.pdf]

Additional file 3: Part 2 Survey

MUDS Pain Survey IRB #6324 Dickersin v2/March 24, 2015

Page 1

Thank you for your interest in participating in this survey.

By entering information into this survey, you are providing your consent for investigators at Johns Hopkins to use the information for research. The survey does NOT ask for your name or for any personally identifiable information, and the researchers will not attempt to identify you.

*1. You must be 18 years or older to participate in this survey.

Are you 18 years or older?

People with pain have many different treatment options. Different treatments have different benefits and side effects that might affect your willingness to choose a drug or continue taking a drug. Benefits are the ways that a drug might make you feel better or improve your life. Side effects are undesirable or harmful effects of a drug.

We want to know which benefits and side effects matter the most to you.

To help us understand your point of view, we will ask about which treatments you have used and some other background information about you.

1. You will see questions based on the month that you were born in.

In which month were you born?

Benefits January

1. You might like to know about the possibility of certain benefits and side effects because they affect your decision about which treatment to take.

Please rank the importance of the following as they relate to your decision to use or not use a treatment. Put the options in order from 1 to 7 such that:

1 MOST affects your decision
7 LEAST affects your decision

You can use the drop-down boxes to select your choices or drag the rows into your preferred order.

[ ]	Pain relief (reduced intensity or severity)
[ ]	Improvement in your ability to do normal activities (social activities, work, school, family life)
[ ]	Improvement in sleep
[ ]	Changes in the overall quality of your life
[ ]	Changes in mood (for example, feeling less anxious or depressed)
[ ]	Reductions in your need for other pain medication
[ ]	Side effects

2. Are there other ways you might want a medication for pain to improve your health or your life (that is, other benefits you're seeking from treatment)?

- ○ Yes
- ○ No

If yes, what are these benefits?

[ ]

Benefits February

1. You might like to know about the possibility of certain benefits and side effects because they affect your decision about which treatment to take.

Please rank the importance of the following as they relate to your decision to use or not use a treatment. Put the options in order from 1 to 7 such that:

1 MOST affects your decision
7 LEAST affects your decision

You can use the drop-down boxes to select your choices or drag the rows into your preferred order.

- [ ] Pain relief (reduced intensity or severity)
- [ ] Improvement in your ability to do normal activities (social activities, work, school, family life)
- [ ] Improvement in sleep
- [ ] Changes in the overall quality of your life
- [ ] Changes in mood (for example, feeling less anxious or depressed)
- [ ] Reductions in your need for other pain medication
- [ ] Side effects

2. Are there other ways you might want a medication for pain to improve your health or your life (that is, other benefits you're seeking from treatment)?

- ○ Yes
- ○ No

If yes, what are these benefits?

Benefits March

1. You might like to know about the possibility of certain benefits and side effects because they affect your decision about which treatment to take.

Please rank the importance of the following as they relate to your decision to use or not use a treatment. Put the options in order from 1 to 7 such that:

1 MOST affects your decision
7 LEAST affects your decision

You can use the drop-down boxes to select your choices or drag the rows into your preferred order.

[ ]	Pain relief (reduced intensity or severity)
[ ]	Improvement in your ability to do normal activities (social activities, work, school, family life)
[ ]	Improvement in sleep
[ ]	Changes in the overall quality of your life
[ ]	Changes in mood (for example, feeling less anxious or depressed)
[ ]	Reductions in your need for other pain medication
[ ]	Side effects

2. Are there other ways you might want a medication for pain to improve your health or your life (that is, other benefits you're seeking from treatment)?

- ○ Yes
- ○ No

If yes, what are these benefits?

[ ]

Benefits April

1. You might like to know about the possibility of certain benefits and side effects because they affect your decision about which treatment to take.

Please rank the importance of the following as they relate to your decision to use or not use a treatment. Put the options in order from 1 to 7 such that:

1 MOST affects your decision
7 LEAST affects your decision

You can use the drop-down boxes to select your choices or drag the rows into your preferred order.

- Pain relief (reduced intensity or severity)
- Improvement in your ability to do normal activities (social activities, work, school, family life)
- Improvement in sleep
- Changes in the overall quality of your life
- Changes in mood (for example, feeling less anxious or depressed)
- Reductions in your need for other pain medication
- Side effects

2. Are there other ways you might want a medication for pain to improve your health or your life (that is, other benefits you're seeking from treatment)?

- Yes
- No

If yes, what are these benefits?

Benefits May

1. You might like to know about the possibility of certain benefits and side effects because they affect your decision about which treatment to take.

Please rank the importance of the following as they relate to your decision to use or not use a treatment. Put the options in order from 1 to 7 such that:

1 MOST affects your decision
7 LEAST affects your decision

You can use the drop-down boxes to select your choices or drag the rows into your preferred order.

▾	Pain relief (reduced intensity or severity)
▾	Improvement in your ability to do normal activities (social activities, work, school, family life)
▾	Improvement in sleep
▾	Changes in the overall quality of your life
▾	Changes in mood (for example, feeling less anxious or depressed)
▾	Reductions in your need for other pain medication
▾	Side effects

2. Are there other ways you might want a medication for pain to improve your health or your life (that is, other benefits you're seeking from treatment)?

- ○ Yes
- ○ No

If yes, what are these benefits?

Benefits June

1. You might like to know about the possibility of certain benefits and side effects because they affect your decision about which treatment to take.

Please rank the importance of the following as they relate to your decision to use or not use a treatment. Put the options in order from 1 to 7 such that:

1 MOST affects your decision
7 LEAST affects your decision

You can use the drop-down boxes to select your choices or drag the rows into your preferred order.

[ ]	Pain relief (reduced intensity or severity)
[ ]	Improvement in your ability to do normal activities (social activities, work, school, family life)
[ ]	Improvement in sleep
[ ]	Changes in the overall quality of your life
[ ]	Changes in mood (for example, feeling less anxious or depressed)
[ ]	Reductions in your need for other pain medication
[ ]	Side effects

2. Are there other ways you might want a medication for pain to improve your health or your life (that is, other benefits you're seeking from treatment)?

- ○ Yes
- ○ No

If yes, what are these benefits?

Benefits July

1. You might like to know about the possibility of certain benefits and side effects because they affect your decision about which treatment to take.

Please rank the importance of the following as they relate to your decision to use or not use a treatment. Put the options in order from 1 to 7 such that:

1 MOST affects your decision
7 LEAST affects your decision

You can use the drop-down boxes to select your choices or drag the rows into your preferred order.

- Pain relief (reduced intensity or severity)
- Improvement in your ability to do normal activities (social activities, work, school, family life)
- Improvement in sleep
- Changes in the overall quality of your life
- Changes in mood (for example, feeling less anxious or depressed)
- Reductions in your need for other pain medication
- Side effects

2. Are there other ways you might want a medication for pain to improve your health or your life (that is, other benefits you're seeking from treatment)?

- Yes
- No

If yes, what are these benefits?

Benefits August

1. You might like to know about the possibility of certain benefits and side effects because they affect your decision about which treatment to take.

Please rank the importance of the following as they relate to your decision to use or not use a treatment. Put the options in order from 1 to 7 such that:

1 MOST affects your decision
7 LEAST affects your decision

You can use the drop-down boxes to select your choices or drag the rows into your preferred order.

- Pain relief (reduced intensity or severity)
- Improvement in your ability to do normal activities (social activities, work, school, family life)
- Improvement in sleep
- Changes in the overall quality of your life
- Changes in mood (for example, feeling less anxious or depressed)
- Reductions in your need for other pain medication
- Side effects

2. Are there other ways you might want a medication for pain to improve your health or your life (that is, other benefits you're seeking from treatment)?

- Yes
- No

If yes, what are these benefits?

Benefits September

1. You might like to know about the possibility of certain benefits and side effects because they affect your decision about which treatment to take.

Please rank the importance of the following as they relate to your decision to use or not use a treatment. Put the options in order from 1 to 7 such that:

1 MOST affects your decision
7 LEAST affects your decision

You can use the drop-down boxes to select your choices or drag the rows into your preferred order.

[ ]	Pain relief (reduced intensity or severity)
[ ]	Improvement in your ability to do normal activities (social activities, work, school, family life)
[ ]	Improvement in sleep
[ ]	Changes in the overall quality of your life
[ ]	Changes in mood (for example, feeling less anxious or depressed)
[ ]	Reductions in your need for other pain medication
[ ]	Side effects

2. Are there other ways you might want a medication for pain to improve your health or your life (that is, other benefits you're seeking from treatment)?

- ○ Yes
- ○ No

If yes, what are these benefits?

[ ]

Benefits October

1. You might like to know about the possibility of certain benefits and side effects because they affect your decision about which treatment to take.

Please rank the importance of the following as they relate to your decision to use or not use a treatment. Put the options in order from 1 to 7 such that:

1 MOST affects your decision
7 LEAST affects your decision

You can use the drop-down boxes to select your choices or drag the rows into your preferred order.

☐	Pain relief (reduced intensity or severity)
☐	Improvement in your ability to do normal activities (social activities, work, school, family life)
☐	Improvement in sleep
☐	Changes in the overall quality of your life
☐	Changes in mood (for example, feeling less anxious or depressed)
☐	Reductions in your need for other pain medication
☐	Side effects

2. Are there other ways you might want a medication for pain to improve your health or your life (that is, other benefits you're seeking from treatment)?

- ○ Yes
- ○ No

If yes, what are these benefits?

Benefits November

1. You might like to know about the possibility of certain benefits and side effects because they affect your decision about which treatment to take.

Please rank the importance of the following as they relate to your decision to use or not use a treatment. Put the options in order from 1 to 7 such that:

1 MOST affects your decision
7 LEAST affects your decision

You can use the drop-down boxes to select your choices or drag the rows into your preferred order.

▾	Pain relief (reduced intensity or severity)
▾	Improvement in your ability to do normal activities (social activities, work, school, family life)
▾	Improvement in sleep
▾	Changes in the overall quality of your life
▾	Changes in mood (for example, feeling less anxious or depressed)
▾	Reductions in your need for other pain medication
▾	Side effects

2. Are there other ways you might want a medication for pain to improve your health or your life (that is, other benefits you're seeking from treatment)?

- ○ Yes
- ○ No

If yes, what are these benefits?

Benefits December

1. You might like to know about the possibility of certain benefits and side effects because they affect your decision about which treatment to take.

Please rank the importance of the following as they relate to your decision to use or not use a treatment. Put the options in order from 1 to 7 such that:

1 MOST affects your decision
7 LEAST affects your decision

You can use the drop-down boxes to select your choices or drag the rows into your preferred order.

☐	Pain relief (reduced intensity or severity)
☐	Improvement in your ability to do normal activities (social activities, work, school, family life)
☐	Improvement in sleep
☐	Changes in the overall quality of your life
☐	Changes in mood (for example, feeling less anxious or depressed)
☐	Reductions in your need for other pain medication
☐	Side effects

2. Are there other ways you might want a medication for pain to improve your health or your life (that is, other benefits you're seeking from treatment)?

- ○ Yes
- ○ No

If yes, what are these benefits?

Safety January

1. Specific side effects might affect your decision about which drug to take.

This survey asks you to rank a few of the many possible side effects that might be important to you. If the list below does not include side effects that you consider most important, you can enter them in the next question.

For these seven items, please rank the importance of the following side effects as they would relate to your decision to use or not use a drug.

Put the options in order from 1 to 7 such that:

1 MOST affects your decision
7 LEAST affects your decision

(Use the drop-down boxes to select your choices or drag the rows into your preferred order.)

- [ ] Nightmares
- [ ] Loss of sex drive
- [ ] Fainting, difficulty balancing, feeling unsteady
- [ ] Coughing
- [ ] Abnormal results from a blood test
- [ ] Decreased sense of touch
- [ ] Depression or low mood

2. Are there other potential side effects you want to know about before starting a medication?

- Yes
- No

If yes, what are the other possible side effects?

3. When you decide if you want to take a new medication, which is more important to you, (i) the likelihood that the medication will reduce your symptoms or (ii) the likelihood that you will experience side effects?

- The likelihood of feeling better is most important
- The likelihood of side effects is most important
- Feeling better and side effects are equally important

Other (please specify)

Safety February

1. Specific side effects might affect your decision about which drug to take.

This survey asks you to rank a few of the many possible side effects that might be important to you. If the list below does not include side effects that you consider most important, you can enter them in the next question.

For these seven items, please rank the importance of the following side effects as they would relate to your decision to use or not use a drug.

Put the options in order from 1 to 7 such that:

1 MOST affects your decision
7 LEAST affects your decision

(Use the drop-down boxes to select your choices or drag the rows into your preferred order.)

▼	Memory loss or difficulty thinking clearly
▼	Daytime sleepiness, feeling tired
▼	Involuntary muscle movements (e.g., twitching, trembling, rigid muscles, muscle spasms)
▼	Feeling unusually angry or aggressive
▼	Weight gain
▼	Hair or nail loss or discoloration
▼	Gastrointestinal problems (diarrhea, constipation, pain, bloating, indigestion, nausea, vomiting)

2. Are there other potential side effects you want to know about before starting a medication?

- Yes
- No

If yes, what are the other possible side effects?

3. When you decide if you want to take a new medication, which is more important to you, (i) the likelihood that the medication will reduce your symptoms or (ii) the likelihood that you will experience side effects?

- The likelihood of feeling better is most important
- The likelihood of side effects is most important
- Feeling better and side effects are equally important

Other (please specify)

Safety March

1. Specific side effects might affect your decision about which drug to take.

This survey asks you to rank a few of the many possible side effects that might be important to you. If the list below does not include side effects that you consider most important, you can enter them in the next question.

For these seven items, please rank the importance of the following side effects as they would relate to your decision to use or not use a drug.

Put the options in order from 1 to 7 such that:

1 MOST affects your decision
7 LEAST affects your decision

(Use the drop-down boxes to select your choices or drag the rows into your preferred order.)

[▾]	Death
[▾]	Insomnia (problems getting to sleep or staying asleep)
[▾]	Skin problems (e.g., dry skin, acne, rash)
[▾]	Headache
[▾]	Pain in the joints or muscles
[▾]	Itching, tingling, or burning sensation on the skin
[▾]	Swelling (e.g., in the hands, legs, or face)

2. Are there other potential side effects you want to know about before starting a medication?

- Yes
- No

If yes, what are the other possible side effects?

3. When you decide if you want to take a new medication, which is more important to you, (i) the likelihood that the medication will reduce your symptoms or (ii) the likelihood that you will experience side effects?

- The likelihood of feeling better is most important
- The likelihood of side effects is most important
- Feeling better and side effects are equally important

Other (please specify)

Safety April

1. Specific side effects might affect your decision about which drug to take.

This survey asks you to rank a few of the many possible side effects that might be important to you. If the list below does not include side effects that you consider most important, you can enter them in the next question.

For these seven items, please rank the importance of the following side effects as they would relate to your decision to use or not use a drug.

Put the options in order from 1 to 7 such that:

1 MOST affects your decision
7 LEAST affects your decision

(Use the drop-down boxes to select your choices or drag the rows into your preferred order.)

- Feeling unusually angry or aggressive
- Daytime sleepiness, feeling tired
- Coughing
- Depression or low mood
- Gastrointestinal problems (diarrhea, constipation, pain, bloating, indigestion, nausea, vomiting)
- Decreased sense of touch
- Hair or nail loss or discoloration

2. Are there other potential side effects you want to know about before starting a medication?

- ○ Yes
- ○ No

If yes, what are the other possible side effects?

3. When you decide if you want to take a new medication, which is more important to you, (i) the likelihood that the medication will reduce your symptoms or (ii) the likelihood that you will experience side effects?

- ○ The likelihood of feeling better is most important
- ○ The likelihood of side effects is most important
- ○ Feeling better and side effects are equally important

Other (please specify)

Safety May

1. Specific side effects might affect your decision about which drug to take.

This survey asks you to rank a few of the many possible side effects that might be important to you. If the list below does not include side effects that you consider most important, you can enter them in the next question.

For these seven items, please rank the importance of the following side effects as they would relate to your decision to use or not use a drug.

Put the options in order from 1 to 7 such that:

1 MOST affects your decision
7 LEAST affects your decision

(Use the drop-down boxes to select your choices or drag the rows into your preferred order.)

- [ ] Decreased sense of touch
- [ ] Itching, tingling, or burning sensation on the skin
- [ ] Pain in the joints or muscles
- [ ] Death
- [ ] Depression or low mood
- [ ] Coughing
- [ ] Daytime sleepiness, feeling tired

2. Are there other potential side effects you want to know about before starting a medication?

- Yes
- No

If yes, what are the other possible side effects?

3. When you decide if you want to take a new medication, which is more important to you, (i) the likelihood that the medication will reduce your symptoms or (ii) the likelihood that you will experience side effects?

- The likelihood of feeling better is most important
- The likelihood of side effects is most important
- Feeling better and side effects are equally important

Other (please specify)

Safety June

1. Specific side effects might affect your decision about which drug to take.

This survey asks you to rank a few of the many possible side effects that might be important to you. If the list below does not include side effects that you consider most important, you can enter them in the next question.

For these seven items, please rank the importance of the following side effects as they would relate to your decision to use or not use a drug.

Put the options in order from 1 to 7 such that:

1 MOST affects your decision
7 LEAST affects your decision

(Use the drop-down boxes to select your choices or drag the rows into your preferred order.)

- Nightmares
- Memory loss or difficulty thinking clearly
- Fainting, difficulty balancing, feeling unsteady
- Weight gain
- Loss of sex drive
- Abnormal results from a blood test
- Involuntary muscle movements (e.g., twitching, trembling, rigid muscles, muscle spasms)

2. Are there other potential side effects you want to know about before starting a medication?

- Yes
- No

If yes, what are the other possible side effects?

3. When you decide if you want to take a new medication, which is more important to you, (i) the likelihood that the medication will reduce your symptoms or (ii) the likelihood that you will experience side effects?

- The likelihood of feeling better is most important
- The likelihood of side effects is most important
- Feeling better and side effects are equally important

Other (please specify)

Safety July

1. Specific side effects might affect your decision about which drug to take.

This survey asks you to rank a few of the many possible side effects that might be important to you. If the list below does not include side effects that you consider most important, you can enter them in the next question.

For these seven items, please rank the importance of the following side effects as they would relate to your decision to use or not use a drug.

Put the options in order from 1 to 7 such that:

1 MOST affects your decision
7 LEAST affects your decision

(Use the drop-down boxes to select your choices or drag the rows into your preferred order.)

- [ ] Insomnia (problems getting to sleep or staying asleep)
- [ ] Fainting, difficulty balancing, feeling unsteady
- [ ] Memory loss or difficulty thinking clearly
- [ ] Skin problems (e.g., dry skin, acne, rash)
- [ ] Swelling (e.g., in the hands, legs, or face)
- [ ] Headache
- [ ] Nightmares

2. Are there other potential side effects you want to know about before starting a medication?

- Yes
- No

If yes, what are the other possible side effects?

3. When you decide if you want to take a new medication, which is more important to you, (i) the likelihood that the medication will reduce your symptoms or (ii) the likelihood that you will experience side effects?

- The likelihood of feeling better is most important
- The likelihood of side effects is most important
- Feeling better and side effects are equally important

Other (please specify)

Safety August

1. Specific side effects might affect your decision about which drug to take.

This survey asks you to rank a few of the many possible side effects that might be important to you. If the list below does not include side effects that you consider most important, you can enter them in the next question.

For these seven items, please rank the importance of the following side effects as they would relate to your decision to use or not use a drug.

Put the options in order from 1 to 7 such that:

1 MOST affects your decision
7 LEAST affects your decision

(Use the drop-down boxes to select your choices or drag the rows into your preferred order.)

- [ ] Abnormal results from a blood test
- [ ] Death
- [ ] Depression or low mood
- [ ] Feeling unusually angry or aggressive
- [ ] Fainting, difficulty balancing, feeling unsteady
- [ ] Insomnia (problems getting to sleep or staying asleep)
- [ ] Memory loss or difficulty thinking clearly

2. Are there other potential side effects you want to know about before starting a medication?

- Yes
- No

If yes, what are the other possible side effects?

3. When you decide if you want to take a new medication, which is more important to you, (i) the likelihood that the medication will reduce your symptoms or (ii) the likelihood that you will experience side effects?

- The likelihood of feeling better is most important
- The likelihood of side effects is most important
- Feeling better and side effects are equally important

Other (please specify)

Safety September

1. Specific side effects might affect your decision about which drug to take.

This survey asks you to rank a few of the many possible side effects that might be important to you. If the list below does not include side effects that you consider most important, you can enter them in the next question.

For these seven items, please rank the importance of the following side effects as they would relate to your decision to use or not use a drug.

Put the options in order from 1 to 7 such that:

1 MOST affects your decision
7 LEAST affects your decision

(Use the drop-down boxes to select your choices or drag the rows into your preferred order.)

☐	Itching, tingling, or burning sensation on the skin
☐	Coughing
☐	Swelling (e.g., in the hands, legs, or face)
☐	Daytime sleepiness, feeling tired
☐	Loss of sex drive
☐	Weight gain
☐	Gastrointestinal problems (diarrhea, constipation, pain, bloating, indigestion, nausea, vomiting)

2. Are there other potential side effects you want to know about before starting a medication?

- Yes
- No

If yes, what are the other possible side effects?

3. When you decide if you want to take a new medication, which is more important to you, (i) the likelihood that the medication will reduce your symptoms or (ii) the likelihood that you will experience side effects?

- The likelihood of feeling better is most important
- The likelihood of side effects is most important
- Feeling better and side effects are equally important

Other (please specify)

Safety October

1. Specific side effects might affect your decision about which drug to take.

This survey asks you to rank a few of the many possible side effects that might be important to you. If the list below does not include side effects that you consider most important, you can enter them in the next question.

For these seven items, please rank the importance of the following side effects as they would relate to your decision to use or not use a drug.

Put the options in order from 1 to 7 such that:

1 MOST affects your decision
7 LEAST affects your decision

(Use the drop-down boxes to select your choices or drag the rows into your preferred order.)

- [ ] Headache
- [ ] Involuntary muscle movements (e.g., twitching, trembling, rigid muscles, muscle spasms)
- [ ] Nightmares
- [ ] Decreased sense of touch
- [ ] Hair or nail loss or discoloration
- [ ] Pain in the joints or muscles
- [ ] Skin problems (e.g., dry skin, acne, rash)

2. Are there other potential side effects you want to know about before starting a medication?

- ○ Yes
- ○ No

If yes, what are the other possible side effects?

3. When you decide if you want to take a new medication, which is more important to you, (i) the likelihood that the medication will reduce your symptoms or (ii) the likelihood that you will experience side effects?

- ○ The likelihood of feeling better is most important
- ○ The likelihood of side effects is most important
- ○ Feeling better and side effects are equally important

Other (please specify)

Safety November

1. Specific side effects might affect your decision about which drug to take.

This survey asks you to rank a few of the many possible side effects that might be important to you. If the list below does not include side effects that you consider most important, you can enter them in the next question.

For these seven items, please rank the importance of the following side effects as they would relate to your decision to use or not use a drug.

Put the options in order from 1 to 7 such that:

1 MOST affects your decision
7 LEAST affects your decision

(Use the drop-down boxes to select your choices or drag the rows into your preferred order.)

☐	Headache
☐	Pain in the joints or muscles
☐	Weight gain
☐	Insomnia (problems getting to sleep or staying asleep)
☐	Gastrointestinal problems (diarrhea, constipation, pain, bloating, indigestion, nausea, vomiting)
☐	Skin problems (e.g., dry skin, acne, rash)
☐	Involuntary muscle movements (e.g., twitching, trembling, rigid muscles, muscle spasms)

2. Are there other potential side effects you want to know about before starting a medication?

- Yes
- No

If yes, what are the other possible side effects?

3. When you decide if you want to take a new medication, which is more important to you, (i) the likelihood that the medication will reduce your symptoms or (ii) the likelihood that you will experience side effects?

- The likelihood of feeling better is most important
- The likelihood of side effects is most important
- Feeling better and side effects are equally important

Other (please specify)

Safety December

1. Specific side effects might affect your decision about which drug to take.

This survey asks you to rank a few of the many possible side effects that might be important to you. If the list below does not include side effects that you consider most important, you can enter them in the next question.

For these seven items, please rank the importance of the following side effects as they would relate to your decision to use or not use a drug.

Put the options in order from 1 to 7 such that:

1 MOST affects your decision
7 LEAST affects your decision

(Use the drop-down boxes to select your choices or drag the rows into your preferred order.)

- [ ] Death
- [ ] Itching, tingling, or burning sensation on the skin
- [ ] Feeling unusually angry or aggressive
- [ ] Hair or nail loss or discoloration
- [ ] Swelling (e.g., in the hands, legs, or face)
- [ ] Loss of sex drive
- [ ] Abnormal results from a blood test

2. Are there other potential side effects you want to know about before starting a medication?

- Yes
- No

If yes, what are the other possible side effects?

3. When you decide if you want to take a new medication, which is more important to you, (i) the likelihood that the medication will reduce your symptoms or (ii) the likelihood that you will experience side effects?

- The likelihood of feeling better is most important
- The likelihood of side effects is most important
- Feeling better and side effects are equally important

Other (please specify)

Demographic Questions

1. In which year were you born?

2. What is your sex?

- Male
- Female
- Other (please specify)

3. Do you have pain related to any of these conditions?

- TMJ (temporomandibular joint and muscle disorders)
- Back pain
- Cancer
- Carpal tunnel syndrome
- Chronic pain after a surgery
- Chronic pelvic pain
- Complex regional pain syndrome
- Diabetes mellitus
- Fibromyalgia
- Guillain-Barré syndrome
- Lyme Disease
- Migraine
- Osteoarthritis
- Phantom limb pain
- Restless leg syndrome
- Shingles (postherpetic neuralgia)
- Stroke
- Trigeminal neuralgia
- None of the above

Other (please specify)

4. Using the image below, how would you rate your pain on a 0-10 scale at the present time, right now, where 0 is 'no pain' and 10 is 'worst possible pain'?

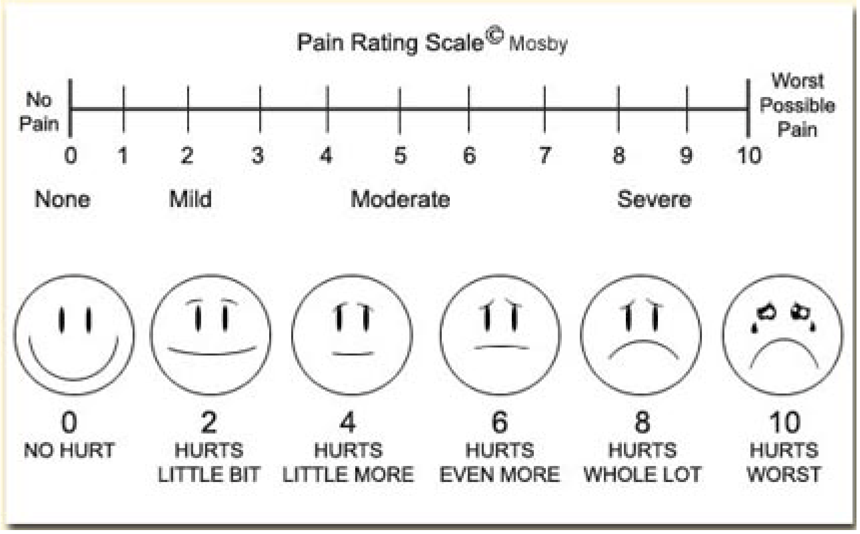
Pain Rating Scale© Mosby
No Pain
Worst Possible Pain
0 1 2 3 4 5 6 7 8 9 10
None
Mild
Moderate
Severe
0
NO HURT
2
HURTS LITTLE BIT
4
HURTS LITTLE MORE
6
HURTS EVEN MORE
8
HURTS WHOLE LOT
10
HURTS WORST

5. What treatments have you taken for pain?

	Taken in the PAST (more than 4 weeks ago)	CURRENTLY taking (within the last 4 weeks)
Ibuprofen (Motrin, Advil)	☐	☐
Aspirin (Bayer, Bufferin, Excedrin)	☐	☐
Naproxen (Aleve)	☐	☐
Acetaminophen (Paracetamol, Tylenol, Panadol)	☐	☐
Oxycodone (Oxycontin, Roxicodone, Oxecta)	☐	☐
Oxycodone with acetaminophen (Percocet)	☐	☐
Hydrocodone	☐	☐
Hydrocodone with acetaminophen (Vicodin, Lortab)	☐	☐
Tramadol (Ultram, ConZip, Ryzolt)	☐	☐
Cyclobenzaprine (Flexeril)	☐	☐
Carisoprodol (Soma)	☐	☐
Gabapentin (Neurontin)	☐	☐
Ketorolac (Toradol)	☐	☐
Diazepam (Valium)	☐	☐
Alprazolam (Xanax)	☐	☐
Clonazepam (Klonopin)	☐	☐
Acupuncture	☐	☐
Splints	☐	☐
Occlusal (Bite) Adjustment (orthodontics, bridges or crowns, teeth grinding)	☐	☐
Injections of corticosteroids	☐	☐
Injections of Botox	☐	☐
Surgery	☐	☐
TMJ Implants	☐	☐
Massage	☐	☐
None of the above	☐	☐

Other (please specify treatment and if taken in past or currently taking)

6. At what age were you diagnosed with a pain disorder?

Other (please specify)

7. Have you had any of the following side effects from a drug, device, or other treatment for your pain?

	Experienced in the PAST (more than 4 weeks ago)	CURRENTLY experiencing (within the last 4 weeks)
Fainting, difficulty balancing, feeling unsteady	☐	☐
Hair or nail loss or discoloration	☐	☐
Depression or low mood	☐	☐
Insomnia (problems getting to sleep or staying asleep)	☐	☐
Loss of sex drive	☐	☐
Itching, tingling, or burning sensation on the skin	☐	☐
Feeling nervous, anxious or on edge	☐	☐
Headache	☐	☐
Decreased sense of touch	☐	☐
Weight gain	☐	☐
Daytime sleepiness, feeling tired	☐	☐
Skin problems (e.g., dry skin, acne, rash)	☐	☐
Involuntary muscle movements (e.g., twitching, trembling, rigid muscles, muscle spasms)	☐	☐
Feeling unusually angry or aggressive	☐	☐
Memory loss or difficulty thinking clearly	☐	☐
Abnormal results from a blood test	☐	☐
Nightmares	☐	☐
Gastrointestinal problems (diarrhea, constipation, pain, bloating, indigestion, nausea, vomiting)	☐	☐
Swelling (e.g., in the hands, legs, or face)	☐	☐
Coughing	☐	☐
Pain in the joints or muscles	☐	☐
Never had a side effect from a drug, device, or other treatment for your pain	☐	☐

Other (please specify the side effect and if you experienced in the past or are currently experiencing)

8. If you have any comments about the questions, the format of the questions or suggestions for future surveys please write them here.

If you have questions about the survey contact the survey moderator at shutfle1@jhmi.edu

Thanks!
The MUDS team
