# Supplementary material for: Methods to identify and prioritize patient-centered outcomes for use in comparative effectiveness research
Source: Pilot Feasibility Stud. 2018 Jun 12;4:95. doi: 10.1186/s40814-018-0284-6 (PMC6047482; doi:10.1186/s40814-018-0284-6)
Supplement: Supplementary file 4 — Patient co-investigator responses. (PDF 2693 kb) [file 40814_2018_284_MOESM4_ESM.pdf]

Additional file 4: Patient co-investigator responses

Gabapentin outcomes		
**Definitely analyze, very important to patients**	**Possibly analyze, sometimes important to patients or important to some patients**	**Do not understand or know what this is**
• Anxiety (self-reported)<br>• Change in pain intensity (mean difference from baseline)<br>• Cognitive dysfunction<br>• Confusion<br>• Constipation<br>• Depression (self-reported)<br>• Discontinuation for any reason (number of participants who discontinued the drug)<br>• Discontinuation because of side effects (number of participants)<br>• Dizziness<br>• Emotional functioning/mood<br>• Fatigue<br>• Harm: benefit analysis<br>• Health-related quality of life<br>• Memory impairment<br>• Number of participants with self-reported improvement<br>• Pain affect (Short Form-McGill Pain Questionnaire)<br>• Participants experiencing one or more side effects (number of participants)<br>• Nausea<br>• Pain interference<br>• Physical activity<br>• Quality of life (daily pain score measured on an 11-point Likert scale)<br>• Serious adverse events (number of each event)<br>• Sexual dysfunction (patient write in)<br>• Somnolence<br>• Sleep	• Mean total SF-MPQ pain Scores<br>• Number of participants with clinician-reported improvement<br>• Number of responders (participants reporting clinically important reduction in daily pain intensity)<br>• Pain severity score	

Additional file 4: Patient co-investigator responses

Gabapentin outcomes		
**Definitely analyze, very important to patients**	**Possibly analyze, sometimes important to patients or important to some patients**	**Do not understand or know what this is**
• Anxiety (self-reported)<br>• Change in pain intensity (mean difference from baseline)<br>• Cognitive dysfunction<br>• Confusion<br>• Constipation<br>• Depression (self-reported)<br>• Discontinuation for any reason (number of participants who discontinued the drug)<br>• Discontinuation because of side effects (number of participants)<br>• Dizziness<br>• Emotional functioning/mood<br>• Fatigue<br>• Harm: benefit analysis<br>• Health-related quality of life<br>• Memory impairment<br>• Number of participants with self-reported improvement<br>• Pain affect (Short Form-McGill Pain Questionnaire)<br>• Participants experiencing one or more side effects (number of participants)<br>• Nausea<br>• Pain interference<br>• Physical activity<br>• Quality of life (daily pain score measured on an 11-point Likert scale)<br>• Serious adverse events (number of each event)<br>• Sexual dysfunction (patient write in)<br>• Somnolence<br>• Sleep	• Mean total SF-MPQ pain Scores<br>• Number of participants with clinician-reported improvement<br>• Number of responders (participants reporting clinically important reduction in daily pain intensity)<br>• Pain severity score	

Gabapentin outcomes

Definitely analyze, very important to patients	Possibly analyze, sometimes important to patients or important to some patients	Do not understand or know what this is
• Sleep interferences (daily rating)<br>• Sleep interference scores/sleep difficulties<br>• Vertigo<br>• Vomiting		

Quetiapine outcomes

Definitely analyze, very important to patients	Possibly analyze, sometimes important to patients or important to some patients	Do not understand or know what this is
• Change in anxiety (mean difference from baseline on a validated scale)<br>• Change in depression (mean difference from baseline)<br>• Cardiovascular effects (change in QTc interval duration, incidence of orthostatic hypotension)<br>• Change in weight<br>• Diabetes<br>• Discontinuation for any reason (number of participants who discontinued the drug)<br>• Discontinuation because of side effects (number of participants)<br>• Extrapyramidal symptoms (tardive dyskinesia, dystonia, akathisia)<br>• Functioning (mean score on the Global Assessment of Functioning scale)<br>• Health-related quality of life<br>• Hospitalization (number of participants hospitalized)<br>• Inability to concentrate<br>• Loss of energy<br>• Number of responders (participants reporting	• Number of remitters (participants scoring below the cut-off for a clinical episode)	• Change in fasting glucose level<br>• Change in triglycerides<br>• Hematologic effects (incidence of absolute neutrophil count (ANC) < 100/ml (count data/rate)<br>• Mean change in serum prolactin levels<br>• Mean score for measures of extrapyramidal symptoms (e.g. Abnormal Involuntary Movement Scale, Condensed User's Scale, Simpson-Angus Scale, Barnes Akathesia Rating Scale, or similar scale)

**Quetiapine outcomes**		
**Definitely analyze, very important to patients**	**Possibly analyze, sometimes important to patients or important to some patients**	**Do not understand or know what this is**
clinically important reduction in depression rating)<br>• Participants experiencing any extrapyramidal symptoms<br>• Participants experiencing one or more side effects (number of participants)<br>• Serious adverse events (number of each event)<br>• Specific side effects (number of each side effect organized using standard classifications)<br>• Sexual side-effects (patient write-in)<br>• Suicide<br>• Symptoms related to daytime drowsiness<br>• Weight gain		
