# Supplementary material for: Methods to identify and prioritize patient-centered outcomes for use in comparative effectiveness research
Source: Pilot Feasibility Stud. 2018 Jun 12;4:95. doi: 10.1186/s40814-018-0284-6 (PMC6047482; doi:10.1186/s40814-018-0284-6)
Supplement: Supplementary file 5 — TMJ survey participant characteristics. (PDF 1546 kb) [file 40814_2018_284_MOESM5_ESM.pdf]

Additional file 5: TMJ survey participant characteristics

Additional file 5a: Current and past treatments taken for pain (N=388)

	Current[1]		Past[2]	
	N	(%)	N	(%)
Ibuprofen (Motrin, Advil)	190	(48.97)	196	(50.52)
Acetaminophen (Paracetamol, Tylenol, Panadol)	118	(30.41)	160	(41.24)
Massage	104	(26.8)	193	(49.74)
Occlusal (Bite) Adjustment	101	(26.03)	159	(40.98)
Splints	100	(25.77)	148	(38.14)
Naproxen (Aleve)	90	(23.2)	153	(39.43)
Cyclobenzaprine (Flexeril)	67	(17.27)	132	(34.02)
Aspirin (Bayer, Bufferin, Excedrin)	60	(15.46)	120	(30.93)
Hydrocodone with acetaminophen (Vicodin, Lortab)	44	(11.34)	123	(31.7)
Gabapentin (Neurontin)	43	(11.08)	103	(26.55)
Tramadol (Ultram, ConZip, Ryzolt)	35	(9.02)	111	(28.61)
Diazepam (Valium)	32	(8.25)	76	(19.59)
Clonazepam (Klonopin)	31	(7.99)	52	(13.4)
Injections of corticosteroids	28	(7.22)	120	(30.93)
Oxycodone (Oxycontin, Roxicodone, Oxecta)	25	(6.44)	73	(18.81)
Acupuncture	25	(6.44)	123	(31.7)
Oxycodone with acetaminophen (Percocet)	25	(6.44)	102	(26.29)
Alprazolam (Xanax)	24	(6.19)	65	(16.75)
Hydrocodone	23	(5.93)	87	(22.42)
Carisoprodol (Soma)	14	(3.61)	44	(11.34)
Surgery	14	(3.61)	115	(29.64)
TMJ implant	14	(3.61)	56	(14.43)
Injections of Botox	13	(3.35)	58	(14.95)
Ketorolac (Toradol)	7	(1.8)	55	(14.18)
None	7	(1.8)	2	(0.52)

[1]Current: Treatments used within the last four weeks before completing the survey
[2]Past: Treatments used more than four weeks prior to the survey

Additional file 5b: Reported side effects from treatments taken for pain (N=388)

	Current[1]		Past[2]	
	N	(%)	N	(%)
Daytime sleepiness, feeling tired	120	(30.93)	132	(34.02)
Gastrointestinal problems (diarrhea, constipation, pain, bloating, indigestion, nausea, vomiting)	106	(27.32)	168	(43.3)
Headache	103	(26.55)	162	(41.75)
Pain in the joints or muscles	101	(26.03)	59	(15.21)
Insomnia (problems getting to sleep or staying asleep)	96	(24.74)	93	(23.97)
Memory loss or difficulty thinking clearly	90	(23.2)	131	(33.76)
Depression or low mood	77	(19.85)	118	(30.41)
Loss of sex drive	71	(18.3)	97	(25)
Involuntary muscle movements (e.g., twitching, trembling, rigid muscles, muscle spasms)	65	(16.75)	83	(21.39)
Feeling nervous, anxious or on edge	65	(16.75)	117	(30.15)
Weight gain	64	(16.49)	116	(29.9)
Fainting, difficulty balancing, feeling unsteady	60	(15.46)	109	(28.09)
Itching, tingling, or burning sensation on the skin	51	(13.14)	93	(23.97)
Skin problems (e.g., dry skin, acne, rash)	35	(9.02)	59	(15.21)
Swelling (e.g., in the hands, legs, or face)	34	(8.76)	133	(34.28)
Hair or nail loss or discoloration	30	(7.73)	41	(10.57)
Nightmares	28	(7.22)	70	(18.04)
Feeling unusually angry or aggressive	26	(6.7)	75	(19.33)
Never had a side effect from a drug, device, or other treatment for your pain	24	(6.19)	26	(6.7)
Decreased sense of touch	19	(4.9)	32	(8.25)
Coughing	14	(3.61)	32	(8.25)
Abnormal results from a blood test	13	(3.35)	43	(11.08)

[1]Current: Side effects experienced within the last four weeks before completing the survey

[2]Past: Side effects experienced more than four weeks prior to the survey
