# Supplementary material for: Methods to identify and prioritize patient-centered outcomes for use in comparative effectiveness research
Source: Pilot Feasibility Stud. 2018 Jun 12;4:95. doi: 10.1186/s40814-018-0284-6 (PMC6047482; doi:10.1186/s40814-018-0284-6)
Supplement: Supplementary file 6 — All “other” potential benefits and harms reported by survey participants in open-ended questions. (PDF 3420 kb) [file 40814_2018_284_MOESM6_ESM.pdf]

Additional file 6: All "other" potential benefits and harms reported by survey participants in open-ended questions

Note: In Additional file 6a and Additional file 6b, we corrected obvious spelling and capitalization errors. We also capitalized the first word of each line. We did not edit responses for word choice or grammar.

Additional file 6a: Responses to the question "Are there other ways you might want a medication for pain to improve your health or your life (that is, other benefits you're seeking from treatment)?"

A medication that eliminates or even reduces chronic pain without horrible side effects -- and that a doctor isn't afraid to prescribe!
A pain medication that didn't require me to take another medication for constipation. It is difficult to have a decent quality of life if you are tied to a bathroom and cant leave the house!
A reduction in pain that allows me to continue to do the normal activities of my day.
Ability to concentrate while taking; less ups and downs in terms of the medication wearing off.
Ability to eat. Improve sex life.
Ability to enjoy a meal without jaw muscle spasms or pain
Ability to take medication during the day that doesn't affect sleepiness, lethargy, or my ability to do my job, so that I may take something that will help when my TMJ has an extreme jump in pain levels.
Able to socialize better because not in so much pain.
Added energy. I noticed while on my pain medication regimen, when I started taking Adipex to lose weight it did not interfere with my pain meds and gave me some energy even though I was still hurting a good bit (normal for my pain regimen)
Antidepressant. Anxiety
Anything that doesn't make it worse
As many options as possible in case a med doesn't work
Be more effective without danger to body work for a longer period of time so I don't have to remember to take it three times a day take less medications
Be part of a long term treatment plan to NOT be dependent or need medication permanently or long-term – to be part of a treatment program with proven efficacy combining other modalities such as physical therapy, perhaps mindfulness training and other self-help pain management techniques etc. Pain medication that is used ONLY on a short-term, as needed basis, rather than daily/permanently.
Benefits to my general health state and related medical conditions
Better function
Better pain control - It's horrible having stabbing pains in your face and neck and constant muscle spasms
By not providing a financial burden! Cost is almost highest priority for me in deciding whether or not I can take a med
Chronic migraines fibro IBS chronic muscle tension and more, id just like to have a life migraines are being treated but I think some of the underline cause is TMJ. Some is genetic.
Cost, ease of getting, reduced social stigma
Decrease the impulse or instinct to constantly clench my jaw
Enable the ability to exercise and spend higher quality of time with family
Energy to do what is needed to get things done
Financial responsibility for treatment
For the medicine to cure or fix or minimize the condition itself
Freedom to eat what I want
Help relax the muscles so face is less twisted
I have already had surgery, but am in pain every day. All prescription medication causes constipation, which is a MAJOR problem for me. I live on medication to fight the constipation, and had to have many surgeries because of this.
I have severe muscle spasms now and need relief, tried many but after a while they don't help. After many years of TMJ, I am now also diagnosed with Oral Mandibular Dystonia and was told it could be related to years of dentures or Medications used.

I like one that would stop the nerve tingling and burning sensation and also for it to control swelling
I sleep fine... But when I wake up I am sore or my jaw and neck feel tired
I want a pain medication that does not cause stomach/intestinal discomfort and can be used frequently without risk. NSAIDS all carry the risk of ulcers or bleeding.
I want the quality of life to improve. This could mean making me feel happier, relieving stress, reducing inflammation, and making it easier for me to have an active life.
I want to be able to chew my food. I am having to blend EVERYTHING because it is hard to chew. It is also hard to talk on the phone, talk generally and swallow.
I would like a medication that I would at least be able to work a job to afford to live a little life outside of work.
I would like medication in the more general area o TMJ as it relates to and now causes severe burning facial pain as well as jaw pain. IT IS HARD TO SEPARATE IF THE PAIN IS FROM MUSCLE, JOINT DEGENERATION, i.e. bone one bone, and wearing away of joint, AND NERVE PAIN. My pain is so much more than I can explain. Trying a sphenopalatine ganglion block soon, and have Botox for migraines now. Also, physical therapists are not really trained in this area and can cause more hard than good! I have a tried every non- narcotic pain reliever AND anti-depressants, etc. AND narcotics. Only on meloxicam and 3500 mg of Tylenol a day and it is not enough
I would like to not feel depressed due to pain every day.
I'd like to be able to relieve pain without fearing that taking medication over lengthy periods will damage other aspects of my health.
I'm sure there are other ways, but at the moment I can't think of anything other than what was said in question 1
Ideally I don't want to end the pain, but solve the problem causing the pain
If medication can relieve pain, it may make it easier to eat, to talk, to laugh. All of these things will improve daily quality of life which can improve mood and satisfaction with life circumstances.
Improved jaw movement, Improved jaw usage
Improvement in hearing loss.
Improvement in sleep.
Improving overall health and say to day ability
Increased range of motion of the jaw
It can relieve pain in other areas aside from TMJ and soddi patients are able to take them.
It would be nice if there was a cure instead of treating whatever the problem is.
Joint pain
Less dependence on the drug; less interactions with other drugs.
Less fatigue
Less pain in my jaw would allow me to eat more types of foods. I haven't been able to eat steak in 7years for example.
Less swelling
Long term affects
Make it easier to talk or make facial expressions, to help work and social life.
Medication isn't a cure it's a Band-Aid
Medication that will help more than one health challenge at a time. Example: TMJ, Fibromyalgia & muscular / skeletal challenges. Help for mental confusion due to drugs (Clearness of mind).
More normal function of my TMJ. Less difficulty eating, less spasm of joint, improved sensation to mouth area (post op nerve damage).
More positive outlook for the future would be direct benefit of successful pain management.
More sleep since I can not take a RX sleep med because of side effects. Also I would want more pain relief
My ability to eat normally again
Nerve pain
NEW PAIN MEDS THAT ACTUALLY WORK AROUND THE CLOCK FOR THOSE OF US WHO HAVE SEVERE PAIN...I BANDAID CANNOT FIX AN UNDERDEVELOPED JAW. BESIDES SLEEPING SEDATIVES THERE ARE NO MEDS FOR SEVERE TMD PEOPLE THAT DECREASE PAIN SO ONE HAVE SOME QUALITY OF LIFE. THE DOCTORS NEED TO GIVE PAIN MEDS & MEDS THAT INSURANCE COVERS SO PARENTS ARE NOT OUT $60,000 AND THINGS GET WORSE AS NO ONE IN SMALLER

AREAS WORK WITH JAW ISSUES AS IT IS NOT COVERED BY MEDICAL OR DENTAL UNLESS CAUSED BY AN AUTO ACCIDENT. WILLING TO TRY ANYTHING.
Not add additional side affects
Not affect my mental focus and give me my energy back
Not feeling medicated and dependent upon taking pain medication to life my life
Not make me gain weight
Not make me nauseous or cause damage to my GI tract
Not sure
Not to become dependent
Overall feeling better
Pain and inflammation are the worst.
Pain free with no side effects
Pain management clinic
Pain reduction without affecting my level of energy/concentration.
Pain relief without harming my weak heart
Preferably the less harmful medications, the better. I don't want it messing up my body in exchange for less jaw pain.
Quality of life
Reduce grinding and discomfort from misalignment
Reduce muscle tightness and inflammation, on the one side of my face that hurts
Reduce pain induced stress
Reduced frequency; 1-2x/week vs. 4-5x/week
Reduced need for more invasive procedures (e.g. surgery, injections)
Reduction of pain with less side effects. Less fatigue and gastro intestinal problems. The many other treatments available non invasive but not covered under most insurance plans. Too many patients are very restricted in coverage to get any relief with too high a copay eligibility to receive any benefit at all.
Relief from the extreme muscle tightness in my mouth, neck and shoulder area.
Restore energy and ambition I have lost through years of dealing with chronic pain with its resulting lowering the overall quality of my life
Similar to ability to do "normal activities"...having improved function of the body part which pain impairs.
Smaller doses that would last longer
Take my migraines away
Take the TMJ pain away so I can chew food instead of always looking for soft food. Also take the pain away so I don't eat for comfort and gain weight. Take the pain away so I feel like talking to people, since it hurts to talk.
That I have control, depending on level of pain of how much medication I need. That the script is written in such a way I can take what I need----less or more (I understand there has to be some limitations) but it is always individual. My decision of how much medication I take also depends on what I'm doing at the time i.e. just resting at home or if I have to drive.
The long acting OxyContin and Dilaudid and another time gabapentin, put immediate rapid weight gain larger than 10 pounds per month. The Dr. did not seem to care that the medication he gave me was a rapid fat factory of 70+ pounds in 7 months! 7 months and I have to carry around the equivalent to 70 lb. bags of dog food! (could not eat a lot due to closed jawjoint etc. So it was definitely the medication that caused this.) which caused other severe health problems due to this. When I was put on normal hydrocodone, the weight gain stopped immediately. The Doctors just have to listen to their patients! My husband is a physician and the doctors refused to listen to him! Those doctors not listening to the problems the medication cause was unethical!
The medications that currently work for me now also help reduce my migraines.
TMJ relief
To be able to feel & think like a normal person again. To not wake up all hours of the night with pain.
To be able to fly in a airplane. Do be able to do activities.
To be able to have a longer acting medication without that foggy feeling or feeling as though you could just fall asleep

on the spot
To be able to open my mouth wider
To be able to wake up in the morning and not have chronic jaw pain and migraines. I also want to have my life back somewhat as possible without pain.
To be out of pain is my one goal
To eventually stop meds - in other words, a medication that will cure!
To free up brain capacity that was being used by being in pain such that my attention span might be improved and I could be more 'present' as opposed to common side effects of pain medication that leave you feeling sort of zombie-like
To have one day of no pain when I wake up!
To not feel drugged or confused.
To relieve the systemic issues (i.e., GI, Dry Eyes, Fevers...
To treat only flare ups when they become severe, but don't have to take medication constantly.
Use of antipsychotic tranquilizers for pain relief from reflex sympathetic dystrophy order. Causing clenching of the teeth uncontrolled pain
Want to be alert and not impaired on pain meds.
With decreased pain, improvement in muscle skeletal pain and spasm decrease on collateral areas related to the TMJ.

Additional file 6b: Responses to the question "Are there other potential side effects you want to know about before starting a medication?"

1.might the medication cause cancer. 2. Might the medication cause synergism? I am taking medication for Hypertension, and Diabetes.
1. Is the medication addictive? 2. More serious side effects e.g. liver damage etc.
1) Digestive issues such as stomach pain, diarrhea, constipation, etc. I often have. 2) Nightmares, hallucinations (I had with codeine)
Absolutely. The worst problem is nausea from pain medications
Addiction
Addiction
addictive qualities
affect on other conditions
Affects on thyroid function. Weakness. Muscle fatigue.
all of them!
All of them!
All side effects
All side effects!
All.
Any
Any adverse affects on my well being.
Any alteration in mental mood, clarity, cloudy thinking (the last question made it hard to indicate how important this is because the other listed side effects were so severe and frankly unusual sounding)
any and all
any and all side effects
Any and all side effects to include "rare" side effects. I am allergic to many medications and many rare side effects pertain to my situation.
ANY and ALL....I found it difficult to rate them in the question above because all of the side effects would be very unpleasant
Any effect on mood, increase in anxiety or depression.
Any interactions with other medications
Any interference with my other medications
Any long term side affects that would affect my future health
Any of them.
Any of them.
Any other side effects
Any possible side effects
Any, and all, that exist
Any!
Anything and everything that might affect your health.
Anything else that might affect my quality of like or could be life-threatening.
Anything! Stupid question...vertigo...rapid heart beat, etc.
Appetite stimulant, grogginess or mental acuity
Blurred vision, altered perception, how it effects the heart and other organs, interactions with other drugs and herbs
Bone loss
Breathing , mood, and alertness
Can I absorb it after my gastric bypass

Can you become addicted to the medication
Cause cancer
Causes cancer
Causes other medical problems
Cognitive changes, perception, and anything that might be undesirably permanent.
Cognitive issues
Cognitive-memory
Constipation and if/how it interacts with other medications I'm on.
Constipation or diarrhea
Constipation/diarrhoea; headaches or migraines; interaction with other medication.
Contraindications with my other medications.
Could the medication be addictive? Might the medication cause anxiety, tension, increased blood pressure?
Damage to heart, lungs, kidneys or bone loss, etc.
Damage to my internal organs with long term use
Damage to organs
Damage to organs (e.g., liver, kidney)
Danger to body organs such as kidneys of heart
Dependency on the new medication. Is it worse than the old medications.
Depression, anxiety, sleepiness, interactions
Difficulty breathing, severe allergic reaction, cancer potential
Difficulty in the respiratory, or cardiac systems. Also contraindications with other medications. Also additive effects with other medications
Digestive system side effects
Dizziness and/ or nausea, risk of stomach bleeding or ulcers, risk of liver or kidney damage, racing heart
Dizziness, getting other diseases, death
Does it damage internal organs. Does it affect other prescription drugs that I take.
Does the drug cause damage to the liver or kidneys. Can the drug cause seizures.
Don't know until I see them
Drowsiness
Drowsiness
Drowsiness, Depression
Drowsiness, seizures, depression.
Drug interactions
Effect on blood pressure and pancreas
Effect on diet (increased/decreased) weight, digestion (gastric reflux, constipation, respiratory system, drowsiness, behavioral changes, addiction possibilities, interaction with other drugs, light headedness/fainting
Effect on internal organs e.g. the liver
Effect on organ functions. Costs. Addictions. Effectiveness.
Effects a medication may have on the liver or kidneys with long term use.
Effects of digestive system, gastrointestinal effects; constipation, diarrhea, circulatory system; blood pressure, clotting, endocrine system; effects toward hormones. Whether pain medication will effect your body creating situations that leads to disease.
Effects on heart, breathing, etc.
Everything about how this solves the problem vs just masking the pain. I'm not in favor of medication unless it heals.
Exacerbating my cardiomyopathy
Excessive weight gain

Feeling of being dizzy or sleepy. Upset stomach and vomiting.
G.I. Effects
Gastro intestinal
Gastrointestinal problems, edema, potential life threats, vision disturbances
Gastrointestinal side effects
Gastrointestinal, interaction with other medications
GI Bleed, cancer, paralysis, stroke, kidney failure.
GI symptoms
Grinding or clenching teeth and muscle tightness
Habit forming. Affect thinking
Habit-forming? Kidney/liver toxicity?
Hallucination, staying awake
Hard to consider taking drugs with many or any adverse side affects. Would likely skip.
Headaches and nausea and weight gain
Headaches, memory loss, sensitivity to sunlight, itching, water retention, seizures, difficulty breathing
Heart palpitations
Higher chance of heart attack or stroke?   Bad withdrawal. Affect asthma?
How affects blood pressure, weight gain. I like to know all side effects and how likely it is to see these side effects .
How it affects my mind. Foggy brain.
How it effects eye sight. Although not a side effect, is it approved for this medical problem or is in trial or helped with some but not approved.
How it effects my day to day functionality (does it turn me into a zombie?) - and interactions with other medications.
How it effects the digestive system.
how it reacts with other drugs
How would it affect my mood/depression/anxiety.
I do my best to do without pain pills. All side effects are too great a risk if they impair my ability to preform my job
I have difficulty focusing on things. I don’t know if that is the medication or fibromyalgia causing that. I also have ringing in my ears and chronic sinus infections. Also, I have no energy. I don't know what is causing these symptoms.
I have IBS and numerous other pain issues. Is this medicine compatible with what I currently use?
I like to know about all possibilities just in case
I like to know all side effects. I hate meds that make me feel like a zombie.
I want a baby and I can't get pregnant with that meds (Lyrica and Dilaudid)
I want to know about all side effects.
I want to know if the medication has any potential internal side effects (kidney/liver function, GERD, ulcers, etc.), I also would want to know if there is potential for joint deterioration over time of use, especially if it's an injectable medication directly to the joint. I want to know if my joint, which is where my pain is, is going to hold up to the medication, or if in the long run, the joint is going to weaken because of the medication. For that matter, I'd want to know if any of my other bones or joints would weaken because of a medication. I would also want to know if it would change things like my sugar levels, iron levels etc. over a long term period. I use these medications long term, so I am always looking at long term side effects, and rarely care about the short term side effects. (Except maybe death, as my ranking shows...)
I want to know of all potential side effects (so just would read patient leaflet included with drug)
I want to know them all...
I would like a list of all possible side effects.
I would like to know about any possible side effects - providers have skipped over that in the past.
I would want to know all potential side effects

I would want to know if a medication would affect my ability to sleep, as TMJ make sleep difficult. I would not want a medication that made me feel foggy or unable to do normal activities. I would not want a medication that upset my stomach or increased my blood sugar.
I'd want to any and all side effects that would affect my body negatively in ANY way no matter how long after I've taken the medication. It needs to be TRANSPARENT of what I'm allowing in order to have less pain.
I'm allergic to over 50 medications, so it's hard list for me. I'd have to literally compare all medications to be able to say.
If it can be taken during pregnancy.
If it can effect sleep
If it effects blood pressure.
If it is addictive.
If it might kill me would probably be #1
If there are any concerns relative to long term use such as damage to other organ sites or heart failure.
Increased chance of blood clots
Increased depression, affects on heart and brain
Increased heart rate
Increased tolerance and long term effects
Insomnia, tremors or racing heart
Insomnia
Interaction with other drugs
Interactions between all of my medications.
Interactions with medications I take on a daily basis.
Interactions with other medications
Interactions with other medications   Can I drive?
Interactions with other meds
Interactions with other meds and clinical trails outcomes relating to side effects and long term negative effects of the drugs.
Interactions with other RX meds I am taking for other illnesses/conditions.
Interactions with other medications
Intestinal problems
Is it addictive
Is the medicine addicting? Does it affect blood pressure?
Is there a possibility of becoming dependent
Issues with long term use.
Itching/rash or anxiety.
Joint damage
Lethargy, respiratory
Libido, nighttime sleepiness, weight gain, skin discoloration, interaction with other medications
Liver and kidney function changes; noticeable weight gain from medication.
Liver damage, stomach problems, loss of appetite
Liver kidney toxicity, chances of heart attack stroke
Liver or kidney toxicity
Liver, kidneys,
Long term affects rashes ability to function in daily routine does it make you too tired
Long term affects especially to liver, kidney and heart function.
Long term effects
Long term effects

Long term effects
Long term effects, carcinogenic in particular, bone loss
long term health effects
Long term relief
Long term side affects or how the medication impacts my internal organs.
Long term side effects, other effects to the body like IBS.
Long term use risks
Long term effects
Loss of ability to focus.
Loss of appetite, depressed mood, fatigue, affecting sleep (either sleeping too much or insomnia)
Loud, hard sneezing.
Lowered immunity, stomach upset, stroke and seizure increase,
Migraines
Memory loss and lack of focus
Mental fog, cognitive effects, confusion Constipation Poor judgment
Mental ones - Can it cause depression and/or suicidal thoughts? Will it increase my anxiety and give me panic attacks? Can it cause a dissociative mental state?
Migraines, seizures, cancer, bone loss
Muscle weakness loss of co ordination balance dizziness. The other serious side effects like passing out. Any severe brain issues like loss of memory or confusion.
Nausea
Nausea
Nausea, constipation, weight gain, loss of appetite, impact on libido
Nausea, heartburn, cancer
Nausea, potential effect on other bodily functions such as digestion, bad breath
Nausea, safe for breastfeeding/pregnancy
Nausea/vomiting
Nausea
Neuromuscular
Not being able to function to do normal things-- groggy
Pain meds have messed up my bowels for the rest of my life. They have caused other problems with my stomach like an ulcer and chronic constipation. Also, with TMJ there are times eating is a major problem which can lead to different vitamin deficiencies and I have found some medications have made this worse or it has caused other problems because one of the few foods I could eat/drink I was not supposed to. It also has been a problem with low energy or being too tired to go outside or do much of anything. That also makes vitamin deficiencies. Many pain meds aren't made for people that can not eat "normal" diet. Constipation is made worse because we can't chew many of the good veggies we need, pain meds also make it worse.
Permanent damage to my body
Possible cardiovascular, genitourinary, hepatic/renal or respiratory side-effects.
Potential for bone fracture or osteoporosis. The hobbies that make my life worth living include rock climbing, extreme cycling endurance events, and skiing, so the chance of a season ending fracture is high up on my list.
Potential long term damage
Raising cholesterol and triglyceride, sex drive, sleepiness
Rapid Weight Gain!!!! That you can eat 3 yogurts per day and still gain a pound!
Reproductive consequences
Respiratory issues i.e., Increase asthmatic problems. Sunlight sensitivity resulting rashes, eye sensitivity Migraine triggers

Risk of dependence
Risk of intestinal bleeding. I was prescribed Cymbalta when they wouldn't give me anything else, which led to more Advil & Aleve which caused a huge upper GI bleed in which I almost died (hemoglobin was down to 6!!)
Seizure possibilities
Seizures
Seizures, tiredness, mood swings.
Sexual dysfunction
Sexual side effects Addictive potential
Sleepiness
Sleepiness, lack of cognitive awareness
Stomach issues
Stomach upset, constipation, diarrhea, memory issues, concentration loss
Stomach/intestinal bleeding risk, ulcer risk. Interactions with other drugs I am taking. Interactions with alcohol.
Stomachaches
Sweating
Swelling, headaches
Tendon issues; joint issues - not just TMJ; carcinogenic issues
Unwanted gastrointestinal effects
Upset stomach, nausea, confusion, unable to concentrate
Vertigo, blurry vision, upset stomach
Vertigo, skin peeling, hyper mania
Vision and bladder control
Weight gain
Weight gain
Weight gain
Weight gain
Weight gain
Weight gain or loss
Weight gain or loss.
Weight gain or weight loss, vision problems, stomach issues
Weight gain or weight loss??
Weight gain, addiction
Weight gain, drowsiness.
Weight gain, nausea, difficulty sleeping, exhaustion
Weight gain, need for additional medications to counter side effects of new drug, loss of memory or cognitive function
Weight gain.
Weight gain.
Weight gain...
Weight gain/loss
Weight gain/loss
Weight loss or gain, heart rate changes,
What organs or interactions with prescriptions it might have on these issues
What all side effects are and what scale will they affect me
What are long term impacts such as stomach issues
What are the most common side effects, especially in women. How medications combined with thyroid replacement medication. I have hypothyroidism, extremely common condition. In many instances I have seen warnings along the

lines of use caution when combining with thyroid replacement, but generally practitioners/pharmacists were not aware of any specific details.
Whether it might affect my thyroid negatively, or make my thyroid medication less effective.
Will it give me dry mouth.
Would want to know all common side effects.
Yes, I'm the type of person who reads any and all side effects on the drug listing from the pharmacy and also do research on line
