# Supplementary material for: Methods to identify and prioritize patient-centered outcomes for use in comparative effectiveness research
Source: Pilot Feasibility Stud. 2018 Jun 12;4:95. doi: 10.1186/s40814-018-0284-6 (PMC6047482; doi:10.1186/s40814-018-0284-6)
Supplement: Supplementary file 7 — Baseline characteristics of survey participants included in the final analysis by the reported month of birth. (PDF 1966 kb) [file 40814_2018_284_MOESM7_ESM.pdf]

Additional file 7: Baseline characteristics of survey participants included in the final analysis by the reported month of birth (N=385)[1]

Additional file 7a: January to June

	January (N=23)		February (N=25)		March (N=25)		April (N=36)		May (N=27)		June (N=31)	
**Median years of age (IQR)**	42	(36,58)	47	(36,54)	55	(45,61)	53	(47,59)	52	(46,62)	50	(38,57)
**Number of women (%)**	22	(95.7)	24	(96.0)	23	(92)	34	(94.4)	25	(92.6)	28	(90.0)
**Median age diagnosed with a pain disorder (IQR)[2]**	26	(19, 37)	29	(20,36)	32	(22,36)	35	(24,45)	29	(19,34)	27	(18,36)
**Median present pain intensity (IQR)**	4	(2, 5)	5	(2, 7)	5	(2, 8)	4	(3, 7)	6	(3, 7)	5	(3, 6)
**Median number of comorbid pain conditions (IQR)**	2	(2, 4)	3	(2, 4)	2	(1, 3)	3	(1, 4)	3	(2, 4)	3	(2, 4)
**Median number of current pain medications (IQR)**	3	(1, 5)	2	(1, 5)	3	(2, 4)	3	(1, 4)	3	(2, 4)	4	(2, 4)
**Median number of past pain medications (IQR)**	8	(3, 12)	6	(3, 10)	6	(3, 13)	8	(4, 12)	6	(3, 12)	6	(3, 11)

Additional file 7b: July to December

	July (N=35)		August (N=33)		September (N=40)		October (N=39)		November (N=30)		December (N=41)	
**Median years of age (IQR)**	53	(46,60)	53	(47,62)	50	(39,59)	57	(46,64)	48	(40,57)	54	(44,62)
**Number of women (%)**	32	(91.4)	27	(81.8)	37	(92.5)	36	(92.3)	28	(93.3)	38	(92.7)
**Median age diagnosed with a pain disorder (IQR)[2]**	34	(24,45)	32	(20,42)	26	(21,35)	31	(21,37)	30	(23,40)	30	(22,35)
**Median present pain intensity (IQR)**	4	(3, 6)	5	(3, 7)	4	(2, 7)	5	(3, 7)	4	(2, 5)	4	(3, 7)
**Median number of comorbid pain conditions (IQR)**	2	(1, 3)	3	(2, 5)	2	(1, 5)	3	(2, 4)	2	(1, 3)	2	(2, 4)
**Median number of current pain medications (IQR)**	3	(1, 4)	3	(2, 4)	3	(1, 4)	4	(2, 5)	3	(1, 4)	2	(1, 5)
**Median number of past pain medications (IQR)**	5	(2, 7)	7	(3, 9)	5	(3, 9)	4	(2, 11)	6.5	(3, 10)	5	(3, 8)

Legend: IQR = inter-quartile range

[1] Characteristics are included for 385 participants. Three participants indicated that they preferred not to give their months of birth.

[2] 26 participants did not indicate the age they were diagnosed with a pain disorder: four were born in Feb, three in March, five in April, one in May, one in June, two in July, four in September, one in October, one in November and four in December
